# Supplementary material for: High-dimensional phenotyping to define the genetic basis of cellular morphology
Source: Nat Commun. 2024 Jan 6;15:347. doi: 10.1038/s41467-023-44045-w (PMC10771466; doi:10.1038/s41467-023-44045-w)
Supplement: Supplementary file 1 — Supplementary Information [file 41467_2023_44045_MOESM1_ESM.pdf]

Supplementary Information

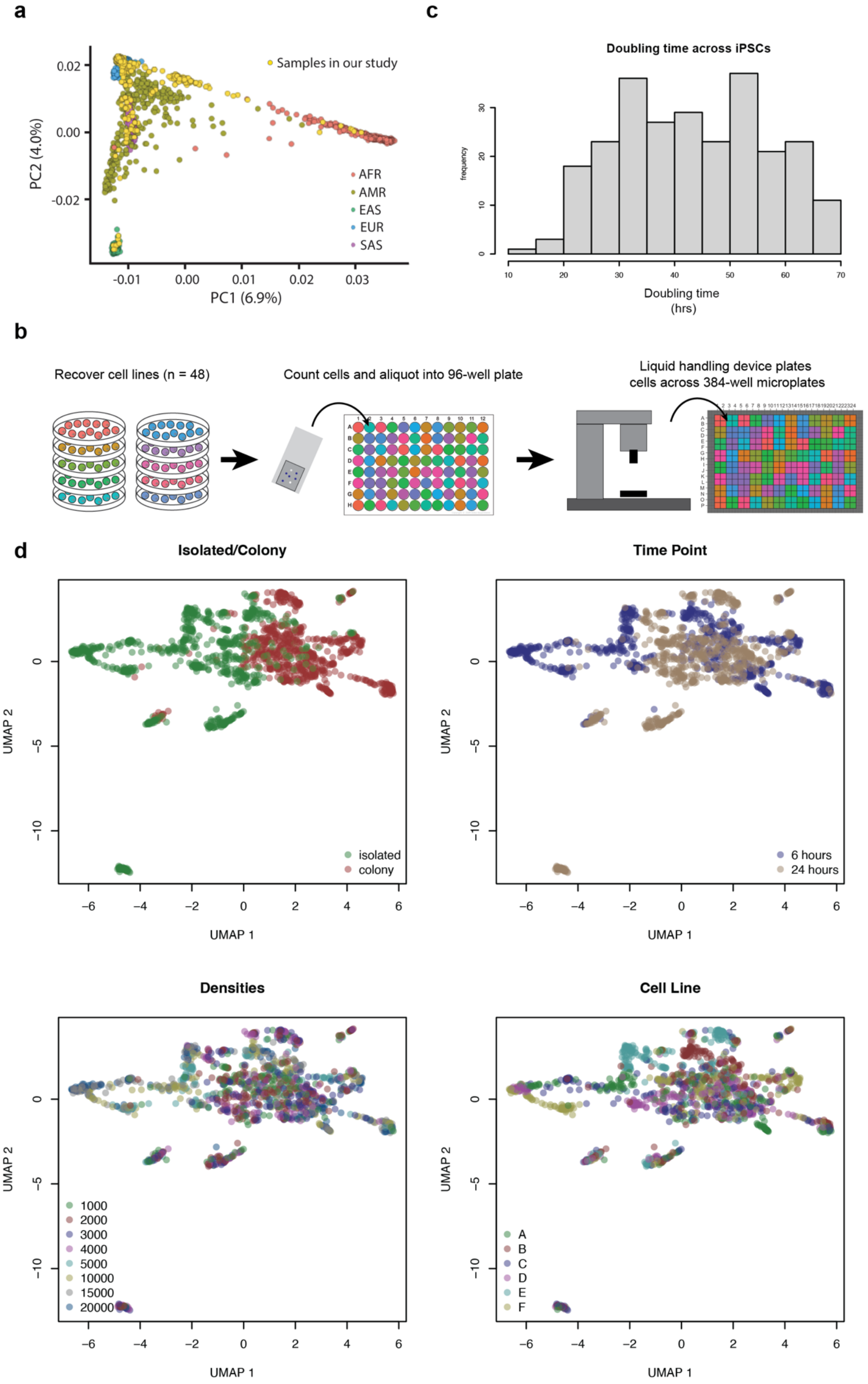

Figure S1. Cell line collection and pilot study. a Distribution of 297 donors (yellow dots) laid over individuals from 1K genomes on PC1 and PC2 calculated from common variants (maf > 5%). Of 297 donors, 207 self-reported their ancestry as European. **b** Cells are thawed in batches of 48 and grown until they reach ~70% confluency. Then cells are dissociated and counted before being aliquoted into deep 96-well plates. A liquid handling device is then used to transfer the cell suspensions from the deep well plates into 384 well high-content screening plates. **c** Doubling time (in hrs) for all 297 cell lines used in this study. Cell growth was calculated during their standard expansion process in house after acquiring them from CIRM. **d** We tested several pilot conditions to identify the ideal parameters for our discovery cohort. We profiled cell lines derived from 6 donors and Cell Painted them under various densities (1000, 2000, 3000, 4000, 5000, 10000, 15000, and 20000 cells/well) and time points for fixation post-plating (6hr and 24hr). We leveraged this data to determine which conditions maximize our ability to measure cell line separation as well as reliably identify cells in both colony and isolate.

a

Data cleaning

Pre-QC dataset contained measurement of 4,300 features for 5.5 million cells imaged across 7 plates

Step 1. Remove a priori known problematic features, costes, correlations and non-numeric features (n=690)

Step 2. Remove features which are not measured in all cells (n=38) or are non-variable (n=9)

Step 3. Remove features which are missing in >5% of cells (n=145)

Step 4. Remove cells which are missing >5% of all features (~400k)

Pre-QC 5.5M cells and 4300 features ⟶ **Post-QC** 5.1M cells and 3418 features

b

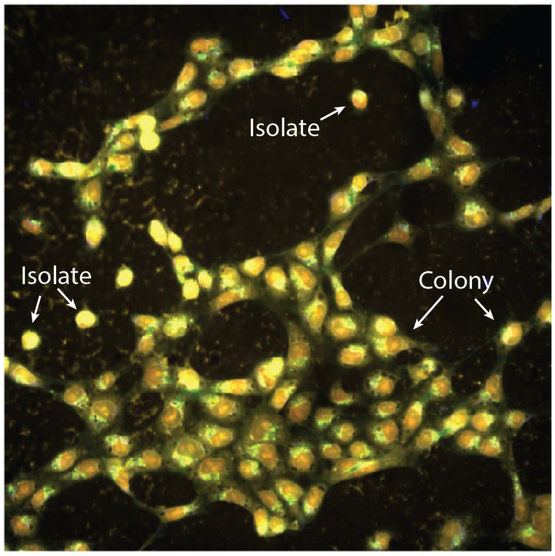

Figure S2. Data QC. **a** A total of 4318 cell morphology traits were quantified across all 5.5 million iPSCs cells from 297 donors. Morphology traits a priori known to be problematic, not measured across all cells or non-variable across cells were removed. Also, cells missing measurement for >5% of traits were removed, yielding 3418 traits across 5.1 million cells. **b** This image is to highlight the variability in cell contexts which we categorize in our study. Cells which do not come in contact with other cells are classified as isolate cells. Those which touch 1 or more cells are classified as colony.

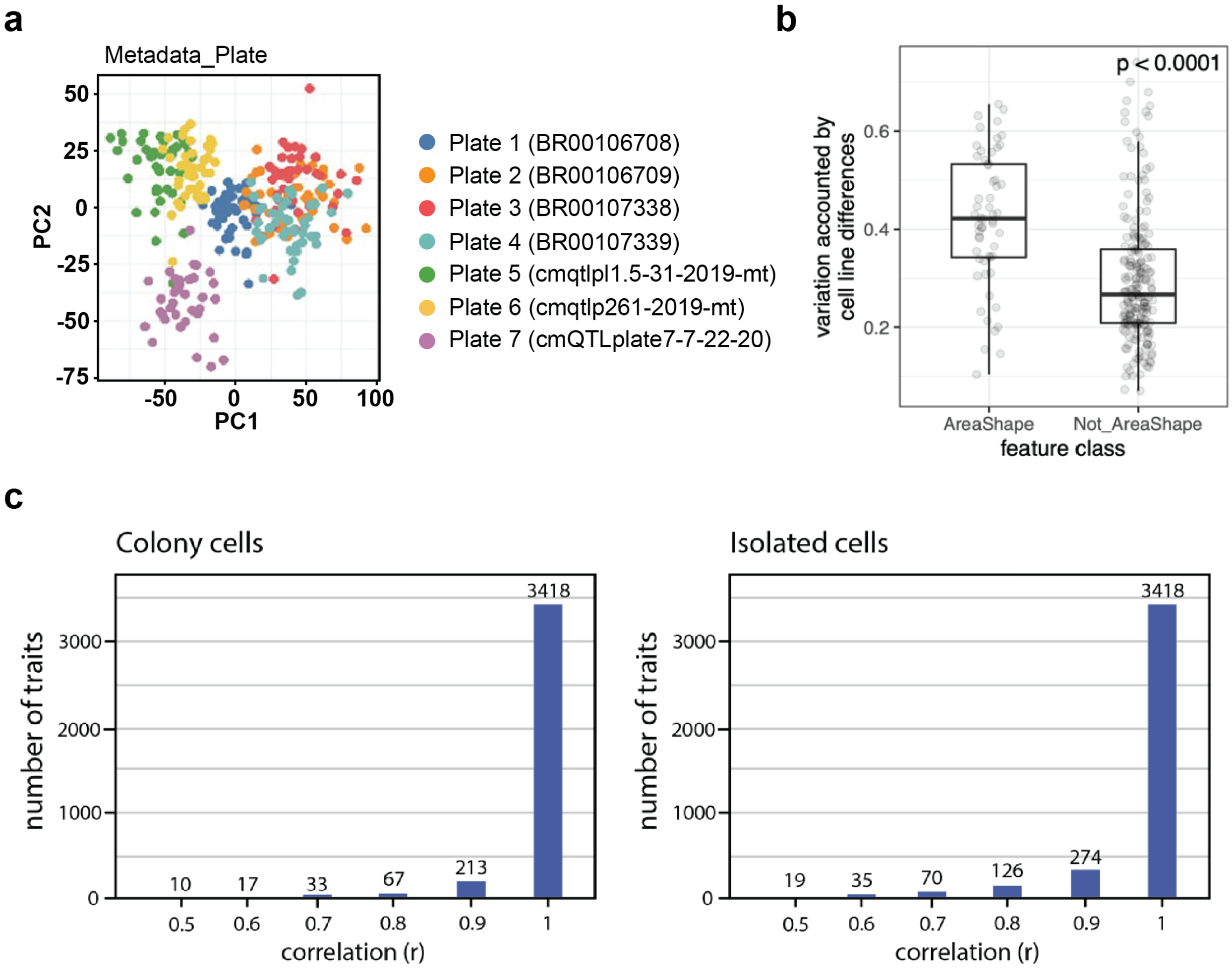

Figure S3. Variance component analysis. a Distribution of 297 donors on PC1 and PC2 calculated from morphology traits (n=3418) colored by 7 plates on which iPSCs from donors were imaged, showing the batch (plate) effect in the measurement of morphology traits. **b** The comparison of variation explained by genetic difference among donors in traits belonging to Area and Shape category and other categories. P-value from Wilcoxon rank sum test is shown. **c** The number of traits having correlation (Pearson r) of up to 0.5, 0.6, 0.7, 0.8, 0.9 and 1 (on x-axis) with at-least one other trait is shown for cells in colonies and cells which are isolated.

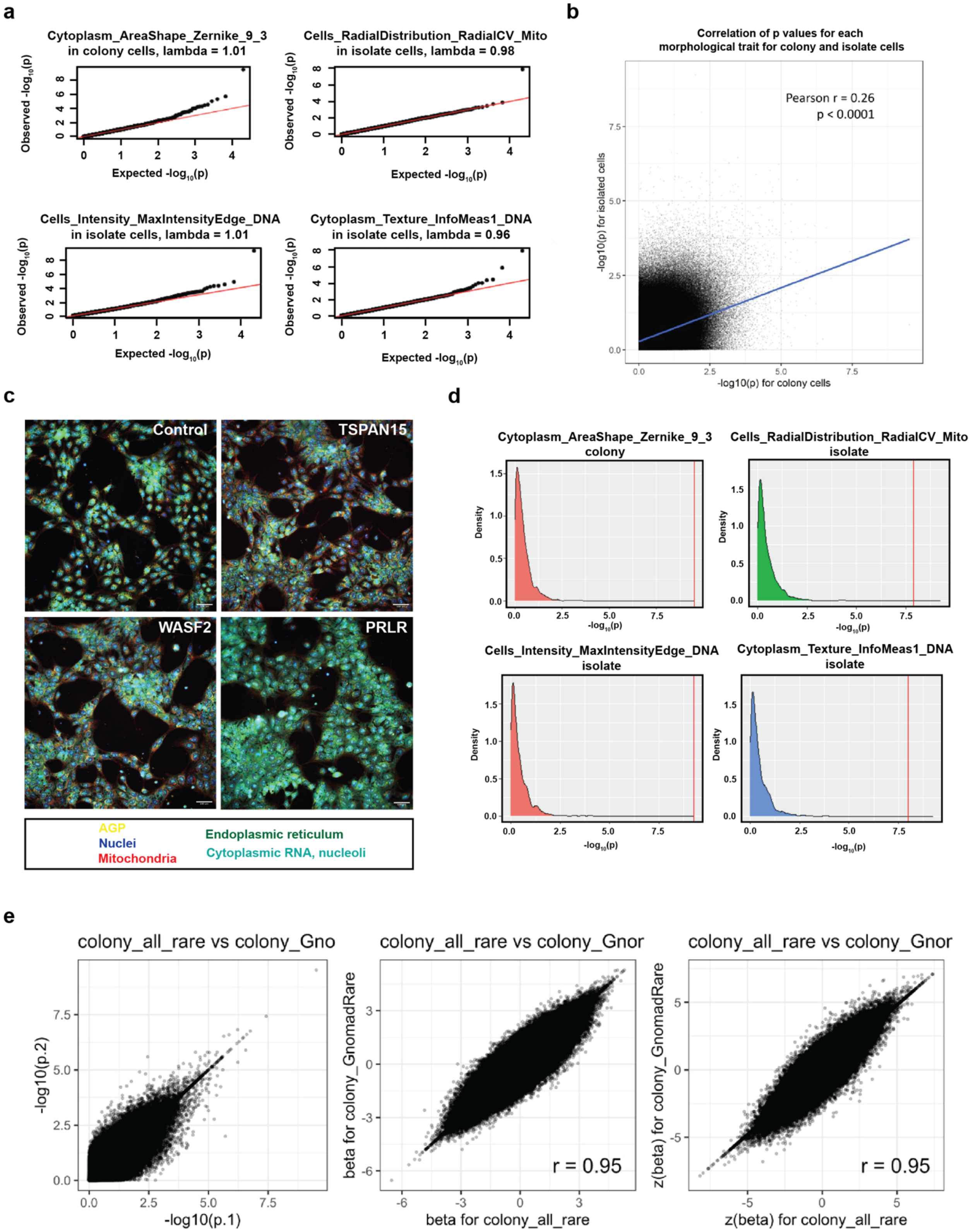

Figure S4. Analyses of rare variant associations. a QQ plots show the distribution of expected and observed p-value of association with all tested genes for 4 morphology traits. Each dot is a tested gene. Lambda statistic (λ), a measure of inflation in observed p-values, is shown. **b** This

figure shows the overall correlation between the p vals for each morphological trait and its association with a gene between cells in isolate and cells in colony. This shows there is modest overall correlation between both cell contexts. **c** Randomly selected representative images from wells containing cell lines harboring rare variants in *WASF2*, *PRLR*, and *TSPAN15* compared to reference cell lines with no detected variants. **d** The distribution of p values following permutations of our rare variant associations. The distribution of p values observed suggests that our associations are unlikely to have occurred by chance. **e** Comparison of p-value and z-score of effect size (beta) of associations between individual morphology traits and rare variant burden in individual genes using all rare variants in our dataset and those rare variants (out of all) which are also present in gnomAD dataset is shown for colony cells. Pearson r is shown.

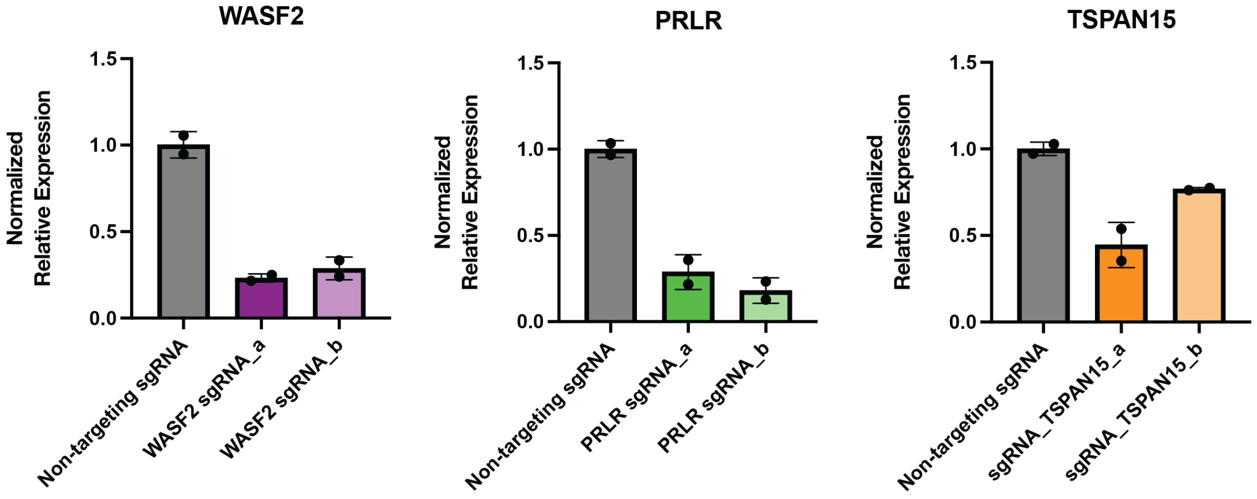

Figure S5. qPCR knockdown of rare-variant associations using CRISPR interference. Relative expression of sgRNA target genes compared to *GAPDH* and *RPL10* between iPSCs transfected with gene targeting sgRNAs and non-targeting control sgRNAs.

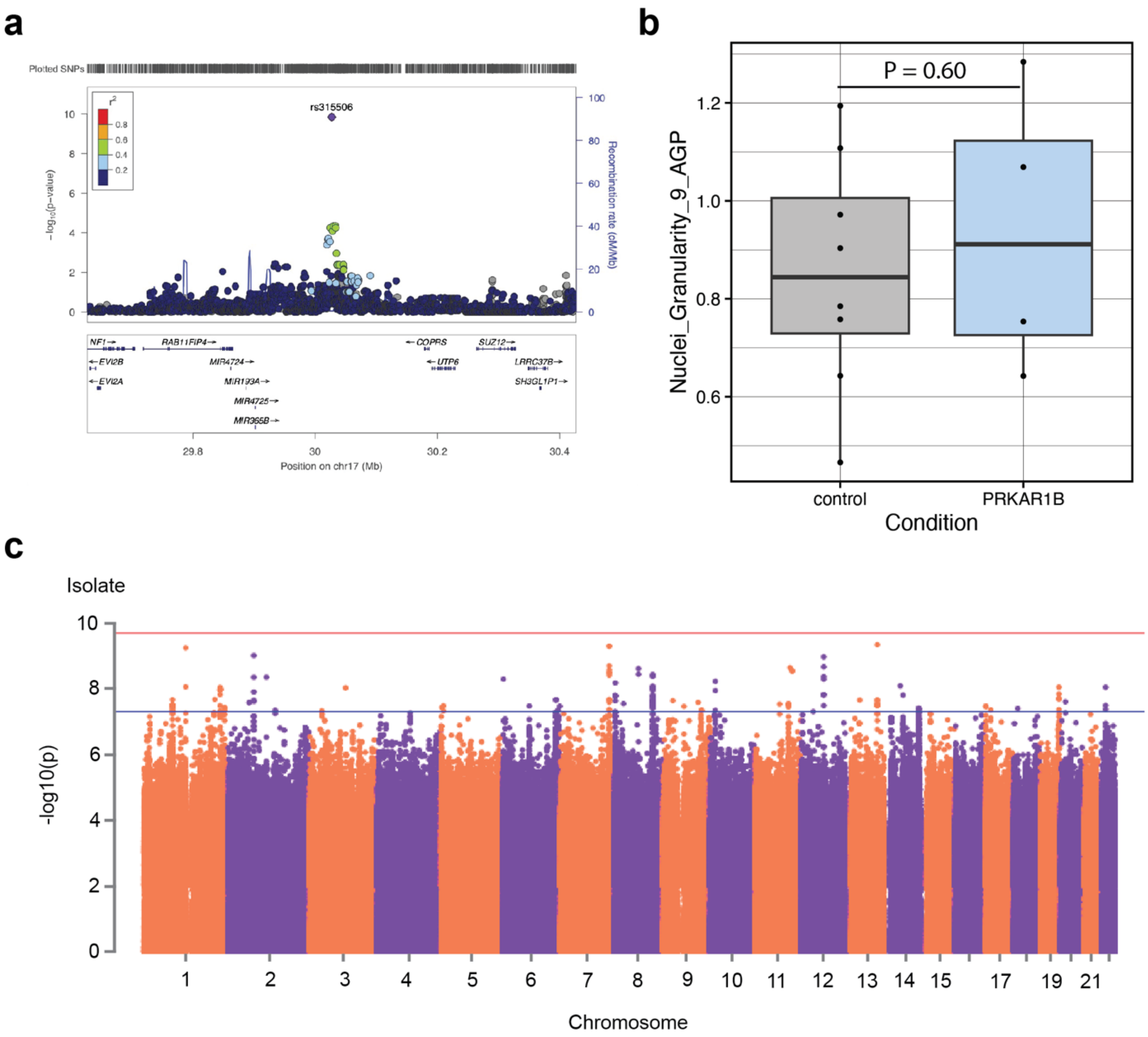

Figure S6. Analyses of common variant associations. a LocusZoom plot for the association signal at chr17q11.2. rs315506, was significantly associated with spatial distribution of cytoplasm (Cytoplasm_RadialDistribution_RadialCV_ER_3of4) in colonies (MAF = 0.08, effect size (se) = -0.52 (0.08), P = 1.4×10^{-10}). **b** Impact of knockdown of *PRKAR1B* in U2OS cells on *Nuclei_Granularity_9_AGP* (P=0.60, Welch's Two-Sample T-Test). **c** Manhattan plot for trait association test in isolate cells. Red line represents the p-value threshold after Bonferroni correction for the number of tested traits and genes ($P < 2 \times 10^{-10}$) Blue line represents the p-value threshold for hits with suggestive evidence ($P < 4.1 \times 10^{-8}$).

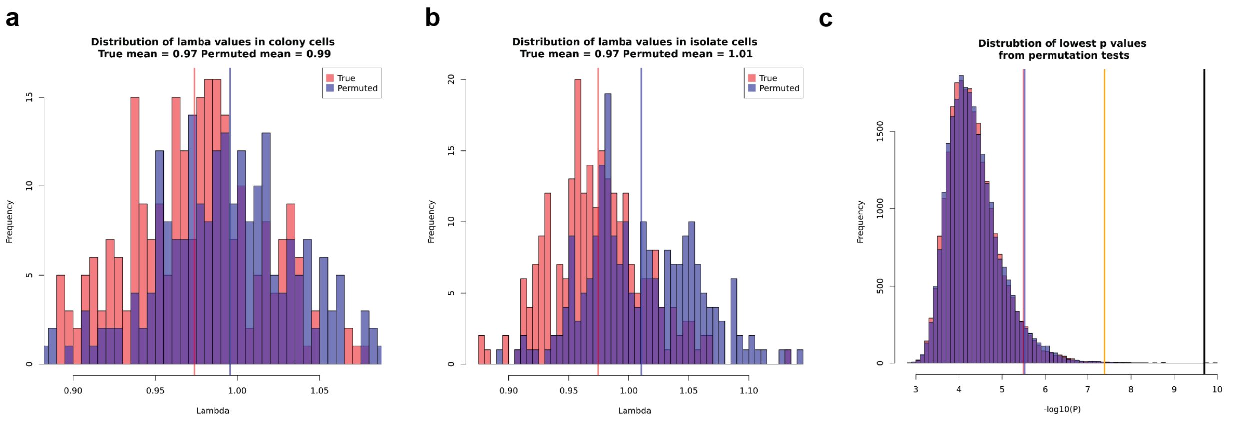

Figure S7. **Permutation of common variant analyses. a** Comparison of lambda value distributions between true and permuted genotypes in colony cells (mean lambda; true = 0.97, permuted = 0.99). **b** Comparison of lambda value distributions between true and permuted genotypes in isolate cells (mean lambda; true = 0.97, permuted = 1.01). **c** Distribution of the lowest p values and 5% FDR threshold based on our perturbation analysis (red = colony cells, blue = isolate cells). Genome-wide (black) and suggestive (orange) threshold from our discovery associations.

Table S1. Metadata for cell lines incorporated in this study.

Metadata characteristics for donors from all 297 iPSC lines included in our study. Table includes donor ID, sex, plate, stem cell source tissue, race, ethnicity, and clinical diagnosis.

DONOR	ALIAS	PLATE	PLATE_ID	AFFECTED_WITH_DISEASE	PRODUCT	SOURCE	SEX	RACE	ETHNICITY	DIAGNOSIS	AGE
CW10152	1	Plate 6	cmqtlpl261-2019-mt	Yes	iPSC	PBMC	Male	Caucasian	Hispanic or Latino	LIVER DISEASE	33
CW20002	2	Plate 5	cmqtlpl1.5-31-2019-mt	Yes	iPSC	PBMC	Female	Caucasian	Not Hispanic or Latino	ASD	6
CW60085	3	Plate 5	cmqtlpl1.5-31-2019-mt	No	iPSC	Fibroblast	Male	Other	Not Hispanic or Latino	CONTROL	46
CW20058	4	Plate 5	cmqtlpl1.5-31-2019-mt	No	iPSC	PBMC	Male	Caucasian	Not Hispanic or Latino	CONTROL	25
CW20063	5	Plate 5	cmqtlpl1.5-31-2019-mt	No	iPSC	PBMC	Female	Caucasian	Not Hispanic or Latino	CONTROL	22
CW20069	6	Plate 5	cmqtlpl1.5-31-2019-mt	No	iPSC	PBMC	Male	Caucasian	Not Hispanic or Latino	CONTROL	5
CW20104	7	Plate 5	cmqtlpl1.5-31-2019-mt	No	iPSC	PBMC	Female	Caucasian	Not Hispanic or Latino	CONTROL	10
CW20245	8	Plate 5	cmqtlpl1.5-31-2019-mt	Yes	iPSC	PBMC	Male	Caucasian	Not Hispanic or Latino	ASD	24
CW20249	9	Plate 5	cmqtlpl1.5-31-2019-mt	Yes	iPSC	PBMC	Male	Caucasian	Not Hispanic or Latino	ASD	25
CW20311	10	Plate 5	cmqtlpl1.5-31-2019-mt	Yes	iPSC	PBMC	Female	Caucasian	Not Hispanic or Latino	ASD	13
CW20320	11	Plate 5	cmqtlpl1.5-31-2019-mt	No	iPSC	PBMC	Female	Caucasian	Not Hispanic or Latino	CONTROL	7
CW20324	12	Plate 5	cmqtlpl1.5-31-2019-mt	No	iPSC	PBMC	Male	Caucasian	Not Hispanic or Latino	CONTROL	4
CW30053	13	Plate 5	cmqtlpl1.5-31-2019-mt	Yes	iPSC	PBMC	Male	Caucasian	Not Hispanic or Latino	HEART DISEASE	78
CW30095	14	Plate 5	cmqtlpl1.5-31-2019-mt	Yes	iPSC	PBMC	Male	Caucasian	Not Hispanic or Latino	HEART DISEASE	44
CW30171	15	Plate 5	cmqtlpl1.5-31-2019-mt	No	iPSC	PBMC	Female	Caucasian	Not Hispanic or Latino	CONTROL	37
CW30438	16	Plate 5	cmqtlpl1.5-31-2019-mt	Yes	iPSC	PBMC	Female	Caucasian	Not Hispanic or Latino	HEART DISEASE	69
CW30453	17	Plate 5	cmqtlpl1.5-31-2019-mt	Yes	iPSC	PBMC	Male	Caucasian	Not Hispanic or Latino	HEART DISEASE	65

CW30463	18	Plate 5	cmqtlpl1.5-31-2019-mt	Yes	iPSC	PBMC	Male	Caucasian	Not Hispanic or Latino	HEART DISEASE	40
CW60039	19	Plate 5	cmqtlpl1.5-31-2019-mt	Yes	iPSC	PBMC	Male	Caucasian	Not Hispanic or Latino	ASD	5
CW60051	20	Plate 5	cmqtlpl1.5-31-2019-mt	Yes	iPSC	Fibroblast	Male	Other	Not Hispanic or Latino	ID	16
CW60053	21	Plate 5	cmqtlpl1.5-31-2019-mt	No	iPSC	Fibroblast	Male	Other	Not Hispanic or Latino	CONTROL	25
CW60054	22	Plate 5	cmqtlpl1.5-31-2019-mt	No	iPSC	Fibroblast	Male	Other	Not Hispanic or Latino	CONTROL	54
CW60057	23	Plate 5	cmqtlpl1.5-31-2019-mt	No	iPSC	Fibroblast	Male	Other	Not Hispanic or Latino	CONTROL	41
CW60084	24	Plate 5	cmqtlpl1.5-31-2019-mt	Yes	iPSC	Fibroblast	Male	Other	Not Hispanic or Latino	ID	14
CW60086	25	Plate 5	cmqtlpl1.5-31-2019-mt	Yes	iPSC	Fibroblast	Male	Other	Not Hispanic or Latino	EPILEPSY	2
CW60087	26	Plate 5	cmqtlpl1.5-31-2019-mt	Yes	iPSC	Fibroblast	Male	Other	Not Hispanic or Latino	EPILEPSY	2
CW60094	27	Plate 5	cmqtlpl1.5-31-2019-mt	Yes	iPSC	PBMC	Male	Other	Not Hispanic or Latino	ID	11
CW60095	28	Plate 5	cmqtlpl1.5-31-2019-mt	No	iPSC	PBMC	Male	Other	Not Hispanic or Latino	CONTROL	?
CW60134	29	Plate 5	cmqtlpl1.5-31-2019-mt	No	iPSC	PBMC	Female	Caucasian	Not Hispanic or Latino	CONTROL	44
CW60135	30	Plate 5	cmqtlpl1.5-31-2019-mt	Yes	iPSC	PBMC	Male	Caucasian	Not Hispanic or Latino	ASD	11
CW60144	31	Plate 5	cmqtlpl1.5-31-2019-mt	Yes	iPSC	Fibroblast	Female	Other	Not Hispanic or Latino	ID	15
CW60163	32	Plate 5	cmqtlpl1.5-31-2019-mt	No	iPSC	Fibroblast	Female	Other	Not Hispanic or Latino	CONTROL	28
CW60164	33	Plate 5	cmqtlpl1.5-31-2019-mt	Yes	iPSC	Fibroblast	Female	Other	Not Hispanic or Latino	ID	4
CW60166	34	Plate 5	cmqtlpl1.5-31-2019-mt	No	iPSC	Fibroblast	Male	Other	Not Hispanic or Latino	CONTROL	26
CW60167	35	Plate 5	cmqtlpl1.5-31-2019-mt	Yes	iPSC	Fibroblast	Male	Other	Not Hispanic or Latino	ID	2
CW60181	36	Plate 5	cmqtlpl1.5-31-2019-mt	Yes	iPSC	Fibroblast	Female	Other	Not Hispanic or Latino	EPILEPSY	4
CW60182	37	Plate 5	cmqtlpl1.5-31-2019-mt	Yes	iPSC	Fibroblast	Female	Other	Not Hispanic or Latino	EPILEPSY	7
CW60184	38	Plate 5	cmqtlpl1.5-31-2019-mt	No	iPSC	Fibroblast	Female	Other	Not Hispanic or Latino	CONTROL	26
CW60186	39	Plate 5	cmqtlpl1.5-31-2019-mt	Yes	iPSC	Fibroblast	Female	Other	Not Hispanic or Latino	ID	2
CW60188	40	Plate 5	cmqtlpl1.5-31-2019-mt	Yes	iPSC	Fibroblast	Male	Other	Not Hispanic or Latino	CEREBRAL PALSY	?
CW60193	41	Plate 5	cmqtlpl1.5-31-2019-mt	Yes	iPSC	Fibroblast	Male	Other	Not Hispanic or Latino	EPILEPSY	5
CW60195	42	Plate 5	cmqtlpl1.5-31-2019-mt	No	iPSC	Fibroblast	Female	Other	Not Hispanic or Latino	CONTROL	28
CW60207	43	Plate 5	cmqtlpl1.5-31-2019-mt	No	iPSC	Fibroblast	Male	Other	Not Hispanic or Latino	CONTROL	27
CW60218	44	Plate 5	cmqtlpl1.5-31-2019-mt	Yes	iPSC	PBMC	Male	Caucasian	Not Hispanic or Latino	ASD	7
CW60228	45	Plate 5	cmqtlpl1.5-31-2019-mt	Yes	iPSC	PBMC	Female	Caucasian	Not Hispanic or Latino	ASD	10
CW60234	46	Plate 5	cmqtlpl1.5-31-2019-mt	Yes	iPSC	PBMC	Male	Caucasian	Hispanic or Latino	EPILEPSY	?
CW60236	47	Plate 5	cmqtlpl1.5-31-2019-mt	Yes	iPSC	PBMC	Female	Caucasian	Hispanic or Latino	EPILEPSY	11
CW60297	48	Plate 5	cmqtlpl1.5-31-2019-mt	Yes	iPSC	Fibroblast	Female	Caucasian	Not Hispanic or Latino	EPILEPSY	5
CW60298	49	Plate 6	cmqtlpl261-2019-mt	Yes	iPSC	Fibroblast	Female	Caucasian	Not Hispanic or Latino	EPILEPSY	2
CW60308	50	Plate 2	BR00106709	No	iPSC	Fibroblast	Female	Caucasian	Not Hispanic or Latino	CONTROL	30
CW60309	51	Plate 6	cmqtlpl261-2019-mt	Yes	iPSC	Fibroblast	Female	Caucasian	Not Hispanic or Latino	ID	10
CW60333	52	Plate 6	cmqtlpl261-2019-mt	Yes	iPSC	Fibroblast	Male	Other	Not Hispanic or Latino	CEREBRAL PALSY	4
CW60334	53	Plate 6	cmqtlpl261-2019-mt	No	iPSC	Fibroblast	Female	Other	Not Hispanic or Latino	CONTROL	38
CW60335	54	Plate 6	cmqtlpl261-2019-mt	Yes	iPSC	Fibroblast	Female	Other	Not Hispanic or Latino	ID	1
CW60353	55	Plate 6	cmqtlpl261-2019-mt	No	iPSC	Fibroblast	Female	Other	Not Hispanic or Latino	CONTROL	23
CW60354	56	Plate 6	cmqtlpl261-2019-mt	Yes	iPSC	Fibroblast	Female	Other	Not Hispanic or Latino	ID	1

CW60362	57	Plate 6	cmqtlpl261-2019-mt	No	iPSC	Fibroblast	Female	Caucasian	Not Hispanic or Latino	CONTROL	29
CW60365	58	Plate 6	cmqtlpl261-2019-mt	No	iPSC	Fibroblast	Male	Caucasian	Not Hispanic or Latino	CONTROL	32
CW60389	59	Plate 6	cmqtlpl261-2019-mt	No	iPSC	Fibroblast	Female	Caucasian	Not Hispanic or Latino	CONTROL	38
CW60391	60	Plate 6	cmqtlpl261-2019-mt	Yes	iPSC	Fibroblast	Female	Caucasian	Not Hispanic or Latino	ID	12
CW60405	61	Plate 6	cmqtlpl261-2019-mt	Yes	iPSC	Fibroblast	Female	Caucasian	Not Hispanic or Latino	ID	7
CW60417	62	Plate 6	cmqtlpl261-2019-mt	Yes	iPSC	Fibroblast	Male	Caucasian	Not Hispanic or Latino	EPILEPSY	5
CW60418	63	Plate 6	cmqtlpl261-2019-mt	No	iPSC	Fibroblast	Female	Caucasian	Not Hispanic or Latino	CONTROL	29
CW60427	64	Plate 6	cmqtlpl261-2019-mt	Yes	iPSC	Fibroblast	Male	Caucasian	Not Hispanic or Latino	CEREBRAL PALSY	10
CW60428	65	Plate 6	cmqtlpl261-2019-mt	No	iPSC	Fibroblast	Male	Caucasian	Not Hispanic or Latino	CONTROL	22
CW60469	66	Plate 6	cmqtlpl261-2019-mt	Yes	iPSC	Fibroblast	Male	Caucasian	Not Hispanic or Latino	EPILEPSY	6
CW60470	67	Plate 6	cmqtlpl261-2019-mt	No	iPSC	Fibroblast	Male	Caucasian	Not Hispanic or Latino	CONTROL	31
CW60477	68	Plate 6	cmqtlpl261-2019-mt	Yes	iPSC	Fibroblast	Male	Caucasian	Not Hispanic or Latino	CEREBRAL PALSY	9
CW60509	69	Plate 6	cmqtlpl261-2019-mt	No	iPSC	Fibroblast	Female	Caucasian	Not Hispanic or Latino	CONTROL	28
CW60510	70	Plate 6	cmqtlpl261-2019-mt	Yes	iPSC	Fibroblast	Female	Caucasian	Not Hispanic or Latino	EPILEPSY	6
CW70190	71	Plate 6	cmqtlpl261-2019-mt	Yes	iPSC	PBMC	Male	Caucasian	Not Hispanic or Latino	GLAUCOMA	79
CW70219	72	Plate 6	cmqtlpl261-2019-mt	Yes	iPSC	PBMC	Male	Caucasian	Not Hispanic or Latino	AMD	77
CW60403	73	Plate 6	cmqtlpl261-2019-mt	Yes	iPSC	Fibroblast	Female	Caucasian	Not Hispanic or Latino	ID	5
CW20012	74	Plate 6	cmqtlpl261-2019-mt	Yes	iPSC	PBMC	Female	Caucasian	Not Hispanic or Latino	ASD	5
CW20025	75	Plate 6	cmqtlpl261-2019-mt	Yes	iPSC	PBMC	Female	Caucasian	Hispanic or Latino	ASD	8
CW20047	76	Plate 6	cmqtlpl261-2019-mt	No	iPSC	PBMC	Male	Caucasian	Not Hispanic or Latino	CONTROL	11
CW20051	77	Plate 6	cmqtlpl261-2019-mt	No	iPSC	PBMC	Female	Caucasian	Not Hispanic or Latino	CONTROL	17
CW20073	78	Plate 6	cmqtlpl261-2019-mt	No	iPSC	PBMC	Male	Caucasian	Not Hispanic or Latino	CONTROL	19
CW20031	79	Plate 6	cmqtlpl261-2019-mt	Yes	iPSC	PBMC	Female	Caucasian	Hispanic or Latino	ASD	7
CW20050	80	Plate 6	cmqtlpl261-2019-mt	Yes	iPSC	PBMC	Male	Caucasian	Not Hispanic or Latino	ASD	6
CW20074	81	Plate 6	cmqtlpl261-2019-mt	No	iPSC	PBMC	Female	Asian	Not Hispanic or Latino	CONTROL	20
CW60092	82	Plate 6	cmqtlpl261-2019-mt	Yes	iPSC	Fibroblast	Female	Other	Not Hispanic or Latino	EPILEPSY	4
CW60138	83	Plate 6	cmqtlpl261-2019-mt	Yes	iPSC	Fibroblast	Female	Caucasian	Not Hispanic or Latino	ID	16
CW60139	84	Plate 6	cmqtlpl261-2019-mt	No	iPSC	Fibroblast	Female	Caucasian	Not Hispanic or Latino	CONTROL	51
CW60303	85	Plate 6	cmqtlpl261-2019-mt	No	iPSC	Fibroblast	Female	Caucasian	Not Hispanic or Latino	CONTROL	51
CW60331	86	Plate 6	cmqtlpl261-2019-mt	Yes	iPSC	Fibroblast	Female	Other	Not Hispanic or Latino	ID	8
CW60383	87	Plate 6	cmqtlpl261-2019-mt	No	iPSC	PBMC	Female	Caucasian	Unknown	CONTROL	40
CW60390	88	Plate 6	cmqtlpl261-2019-mt	Yes	iPSC	Fibroblast	Female	Caucasian	Not Hispanic or Latino	ID	14
CW20041	89	Plate 6	cmqtlpl261-2019-mt	No	iPSC	PBMC	Female	Caucasian	Not Hispanic or Latino	CONTROL	9
CW20075	90	Plate 6	cmqtlpl261-2019-mt	No	iPSC	PBMC	Female	Caucasian	Not Hispanic or Latino	CONTROL	20
CW20077	91	Plate 6	cmqtlpl261-2019-mt	No	iPSC	PBMC	Female	Caucasian	Not Hispanic or Latino	CONTROL	22
CW20081	92	Plate 6	cmqtlpl261-2019-mt	Yes	iPSC	PBMC	Male	Caucasian	Not Hispanic or Latino	ASD	15
CW20084	93	Plate 6	cmqtlpl261-2019-mt	No	iPSC	PBMC	Male	Asiatic Indian	Not Hispanic or Latino	CONTROL	17
CW20087	94	Plate 6	cmqtlpl261-2019-mt	No	iPSC	PBMC	Male	Caucasian	Not Hispanic or Latino	CONTROL	23
CW20091	95	Plate 1	BR00106708	No	iPSC	PBMC	Female	Caucasian	Not Hispanic or Latino	CONTROL	21

CW20094	96	Plate 1	BR00106708	No	iPSC	PBMC	Female	Caucasian	Not Hispanic or Latino	CONTROL	20
CW20103	97	Plate 2	BR00106709	No	iPSC	PBMC	Female	Caucasian	Not Hispanic or Latino	CONTROL	20
CW20105	98	Plate 2	BR00106709	No	iPSC	PBMC	Male	Caucasian	Not Hispanic or Latino	CONTROL	18
CW20107	99	Plate 2	BR00106709	No	iPSC	PBMC	Female	Caucasian	Not Hispanic or Latino	CONTROL	14
CW20108	100	Plate 2	BR00106709	No	iPSC	PBMC	Male	Caucasian	Hispanic or Latino	CONTROL	9
CW20115	101	Plate 2	BR00106709	No	iPSC	PBMC	Female	Caucasian	Hispanic or Latino	CONTROL	13
CW20132	102	Plate 2	BR00106709	No	iPSC	PBMC	Female	Caucasian	Not Hispanic or Latino	CONTROL	14
CW20144	103	Plate 2	BR00106709	No	iPSC	PBMC	Female	Caucasian	Not Hispanic or Latino	CONTROL	22
CW20146	104	Plate 2	BR00106709	No	iPSC	PBMC	Female	Caucasian	Not Hispanic or Latino	CONTROL	12
CW20149	105	Plate 3	BR00107338	No	iPSC	PBMC	Male	Caucasian	Hispanic or Latino	CONTROL	18
CW20166	106	Plate 2	BR00106709	Yes	iPSC	PBMC	Male	Hispanic/Latino	Hispanic or Latino	ASD	13
CW20167	107	Plate 2	BR00106709	No	iPSC	PBMC	Male	Hispanic/Latino	Hispanic or Latino	CONTROL	14
CW20183	108	Plate 2	BR00106709	No	iPSC	PBMC	Male	Caucasian	Not Hispanic or Latino	CONTROL	12
CW20192	109	Plate 2	BR00106709	Yes	iPSC	PBMC	Male	Asian, Other	Not Hispanic or Latino	ASD	6
CW20193	110	Plate 3	BR00107338	No	iPSC	PBMC	Female	Hispanic/Latino	Hispanic or Latino	CONTROL	7
CW20196	111	Plate 2	BR00106709	No	iPSC	PBMC	Male	Caucasian	Not Hispanic or Latino	CONTROL	15
CW20200	112	Plate 3	BR00107338	No	iPSC	PBMC	Female	Hispanic/Latino	Hispanic or Latino	CONTROL	16
CW20203	113	Plate 2	BR00106709	Yes	iPSC	PBMC	Male	Asiatic Indian	Not Hispanic or Latino	ASD	13
CW20210	114	Plate 2	BR00106709	No	iPSC	PBMC	Male	Asiatic Indian	Not Hispanic or Latino	CONTROL	11
CW20213	115	Plate 2	BR00106709	No	iPSC	PBMC	Female	Caucasian	Not Hispanic or Latino	CONTROL	9
CW20214	116	Plate 2	BR00106709	Yes	iPSC	PBMC	Female	Filipino	Not Hispanic or Latino	ASD	15
CW20225	117	Plate 2	BR00106709	Yes	iPSC	PBMC	Male	Filipino	Not Hispanic or Latino	ASD	13
CW20239	118	Plate 2	BR00106709	No	iPSC	PBMC	Female	Caucasian	Not Hispanic or Latino	CONTROL	15
CW20242	119	Plate 2	BR00106709	No	iPSC	PBMC	Male	Hispanic/Latino	Hispanic or Latino	CONTROL	8
CW20243	120	Plate 2	BR00106709	No	iPSC	PBMC	Male	Asian, Other	Not Hispanic or Latino	CONTROL	4
CW50036	121	Plate 2	BR00106709	No	iPSC	PBMC	Female	Caucasian	Not Hispanic or Latino	CONTROL	73
CW50037	122	Plate 2	BR00106709	No	iPSC	PBMC	Female	Caucasian	Not Hispanic or Latino	CONTROL	70
CW60014	123	Plate 2	BR00106709	No	iPSC	Fibroblast	Male	Caucasian	Not Hispanic or Latino	CONTROL	39
CW60015	124	Plate 2	BR00106709	No	iPSC	Fibroblast	Female	Caucasian	Not Hispanic or Latino	CONTROL	39
CW60018	125	Plate 2	BR00106709	Yes	iPSC	Fibroblast	Male	Caucasian	Not Hispanic or Latino	EPILEPSY	3
CW60026	126	Plate 2	BR00106709	Yes	iPSC	PBMC	Female	Caucasian	Not Hispanic or Latino	CEREBRAL PALSY	17

CW60027	127	Plate 2	BR00106709	No	iPSC	Fibroblast	Male	Other	Not Hispanic or Latino	CONTROL	35
CW60048	128	Plate 2	BR00106709	Yes	iPSC	Fibroblast	Female	Other	Not Hispanic or Latino	ID	4
CW60049	129	Plate 2	BR00106709	Yes	iPSC	Fibroblast	Female	Other	Not Hispanic or Latino	ID	6
CW60055	130	Plate 2	BR00106709	Yes	iPSC	PBMC	Male	Caucasian	Not Hispanic or Latino	ASD	8
CW60060	131	Plate 2	BR00106709	No	iPSC	PBMC	Female	Caucasian	Not Hispanic or Latino	CONTROL	37
CW60070	132	Plate 2	BR00106709	Yes	iPSC	Fibroblast	Male	Other	Not Hispanic or Latino	EPILEPSY	9
CW60089	133	Plate 2	BR00106709	Yes	iPSC	PBMC	Female	Caucasian	Not Hispanic or Latino	CEREBRAL PALSY	9
CW60097	134	Plate 2	BR00106709	No	iPSC	PBMC	Female	Caucasian	Not Hispanic or Latino	CONTROL	48
CW60098	135	Plate 2	BR00106709	No	iPSC	Fibroblast	Male	Other	Not Hispanic or Latino	CONTROL	48
CW60108	136	Plate 2	BR00106709	Yes	iPSC	PBMC	Male	Caucasian	Not Hispanic or Latino	ASD	5
CW60109	137	Plate 2	BR00106709	No	iPSC	PBMC	Male	Caucasian	Not Hispanic or Latino	CONTROL	38
CW60122	138	Plate 2	BR00106709	Yes	iPSC	Fibroblast	Male	Other	Not Hispanic or Latino	EPILEPSY	5
CW60123	139	Plate 2	BR00106709	Yes	iPSC	Fibroblast	Female	Other	Not Hispanic or Latino	EPILEPSY	9
CW60124	140	Plate 2	BR00106709	No	iPSC	Fibroblast	Female	Other	Not Hispanic or Latino	CONTROL	29
CW60132	141	Plate 1	BR00106708	No	iPSC	PBMC	Female	Other	Not Hispanic or Latino	CONTROL	32
CW60133	142	Plate 1	BR00106708	Yes	iPSC	PBMC	Female	Other	Not Hispanic or Latino	CEREBRAL PALSY	4
CW60141	143	Plate 1	BR00106708	No	iPSC	Fibroblast	Male	Other	Not Hispanic or Latino	CONTROL	21
CW60142	144	Plate 1	BR00106708	Yes	iPSC	Fibroblast	Male	Other	Not Hispanic or Latino	EPILEPSY	6
CW60143	145	Plate 1	BR00106708	No	iPSC	Fibroblast	Female	Other	Not Hispanic or Latino	CONTROL	17
CW60145	146	Plate 1	BR00106708	Yes	iPSC	Fibroblast	Female	Other	Not Hispanic or Latino	ID	14
CW60171	147	Plate 1	BR00106708	Yes	iPSC	PBMC	Male	Caucasian	Not Hispanic or Latino	ASD	7
CW60172	148	Plate 1	BR00106708	Yes	iPSC	PBMC	Male	Caucasian	Not Hispanic or Latino	ASD	10
CW60174	149	Plate 1	BR00106708	Yes	iPSC	Fibroblast	Male	Other	Not Hispanic or Latino	ID	17
CW60175	150	Plate 1	BR00106708	Yes	iPSC	Fibroblast	Female	Other	Not Hispanic or Latino	ID	7
CW60208	151	Plate 1	BR00106708	Yes	iPSC	Fibroblast	Female	Other	Not Hispanic or Latino	EPILEPSY	6
CW60209	152	Plate 1	BR00106708	Yes	iPSC	Fibroblast	Male	Other	Not Hispanic or Latino	EPILEPSY	5
CW60219	153	Plate 1	BR00106708	Yes	iPSC	PBMC	Male	Caucasian	Not Hispanic or Latino	ASD	13
CW60220	154	Plate 1	BR00106708	No	iPSC	PBMC	Male	Caucasian	Not Hispanic or Latino	CONTROL	9
CW60221	155	Plate 1	BR00106708	Yes	iPSC	PBMC	Female	Caucasian	Not Hispanic or Latino	ID	17
CW60222	156	Plate 1	BR00106708	Yes	iPSC	PBMC	Female	Caucasian	Not Hispanic or Latino	ID	15

CW60223	157	Plate 1	BR00106708	No	iPSC	PBMC	Male	Caucasian	Not Hispanic or Latino	CONTROL	13
CW60242	158	Plate 1	BR00106708	Yes	iPSC	Fibroblast	Female	Other	Not Hispanic or Latino	ID	16
CW60242	159	Plate 1	BR00106708	Yes	iPSC	Fibroblast	Female	Other	Not Hispanic or Latino	ID	16
CW60277	160	Plate 1	BR00106708	Yes	iPSC	Fibroblast	Male	Caucasian	Not Hispanic or Latino	ID	17
CW60278	161	Plate 1	BR00106708	No	iPSC	Fibroblast	Male	Caucasian	Not Hispanic or Latino	CONTROL	39
CW60279	162	Plate 1	BR00106708	No	iPSC	Fibroblast	Female	Caucasian	Not Hispanic or Latino	CONTROL	32
CW60280	163	Plate 1	BR00106708	Yes	iPSC	Fibroblast	Male	Caucasian	Not Hispanic or Latino	ID	2
CW60289	164	Plate 1	BR00106708	No	iPSC	Fibroblast	Female	Caucasian	Not Hispanic or Latino	CONTROL	39
CW60290	165	Plate 1	BR00106708	Yes	iPSC	Fibroblast	Female	Caucasian	Not Hispanic or Latino	ID	18
CW60302	166	Plate 1	BR00106708	Yes	iPSC	Fibroblast	Male	Caucasian	Not Hispanic or Latino	CEREBRAL PALSY	24
CW60304	167	Plate 1	BR00106708	No	iPSC	Fibroblast	Male	Caucasian	Not Hispanic or Latino	CONTROL	40
CW60330	168	Plate 1	BR00106708	No	iPSC	Fibroblast	Female	Other	Not Hispanic or Latino	CONTROL	27
CW60384	169	Plate 1	BR00106708	Yes	iPSC	PBMC	Female	Caucasian	Unknown	ID	5
CW60404	170	Plate 1	BR00106708	No	iPSC	Fibroblast	Female	Caucasian	Not Hispanic or Latino	CONTROL	28
CW60408	171	Plate 1	BR00106708	Yes	iPSC	Fibroblast	Male	Caucasian	Not Hispanic or Latino	ID	18
CW60410	172	Plate 1	BR00106708	No	iPSC	Fibroblast	Female	Caucasian	Not Hispanic or Latino	CONTROL	26
CW60412	173	Plate 1	BR00106708	No	iPSC	Fibroblast	Female	Caucasian	Not Hispanic or Latino	CONTROL	32
CW60456	174	Plate 1	BR00106708	Yes	iPSC	Fibroblast	Female	Caucasian	Not Hispanic or Latino	CEREBRAL PALSY	7
CW60457	175	Plate 1	BR00106708	No	iPSC	Fibroblast	Male	Caucasian	Not Hispanic or Latino	CONTROL	36
CW60503	176	Plate 1	BR00106708	Yes	iPSC	Fibroblast	Female	Other	Not Hispanic or Latino	ID	10
CW60504	177	Plate 1	BR00106708	Yes	iPSC	Fibroblast	Male	Other	Not Hispanic or Latino	ID	7
CW60520	178	Plate 1	BR00106708	No	iPSC	Fibroblast	Male	Caucasian	Not Hispanic or Latino	CONTROL	38
CW60521	179	Plate 1	BR00106708	Yes	iPSC	Fibroblast	Male	Caucasian	Not Hispanic or Latino	ID	7
CW70347	180	Plate 1	BR00106708	No	iPSC	PBMC	Female	Caucasian	Not Hispanic or Latino	CONTROL	74
CW70348	181	Plate 1	BR00106708	Yes	iPSC	PBMC	Female	Caucasian	Not Hispanic or Latino	AMD	44
CW20053	182	Plate 1	BR00106708	Yes	iPSC	PBMC	Male	Caucasian	Not Hispanic or Latino	ASD	5
CW60079	183	Plate 1	BR00106708	Yes	iPSC	Fibroblast	Male	Caucasian	Not Hispanic or Latino	ID	14
CW10054	184	Plate 1	BR00106708	No	iPSC	PBMC	Female	African American	Not Hispanic or Latino	CONTROL	40
CW10058	185	Plate 1	BR00106708	Yes	iPSC	Fibroblast	Female	Caucasian	Not Hispanic or Latino	LIVER DISEASE	56
CW10077	186	Plate 1	BR00106708	Yes	iPSC	PBMC	Female	Caucasian	Hispanic or Latino	FATTY LIVER DISEASE	58

CW10086	187	Plate 3	BR00107338	Yes	iPSC	Fibroblast	Male	Caucasian	Hispanic or Latino	LIVER DISEASE	55
CW10100	188	Plate 3	BR00107338	Yes	iPSC	Fibroblast	Male	African American	Not Hispanic or Latino	LIVER DISEASE	51
CW10101	189	Plate 4	BR00107339	Yes	iPSC	Fibroblast	Male	More than one race	Hispanic or Latino	LIVER DISEASE	41
CW10162	190	Plate 4	BR00107339	Yes	iPSC	Fibroblast	Male	African American	Not Hispanic or Latino	LIVER DISEASE	63
CW10192	191	Plate 4	BR00107339	No	iPSC	PBMC	Female	Caucasian	Hispanic or Latino	CONTROL	31
CW11072	192	Plate 4	BR00107339	Yes	iPSC	PBMC	Female	Asian	Not Hispanic or Latino	MDD	68
CW20055	193	Plate 4	BR00107339	Yes	iPSC	PBMC	Male	Caucasian	Hispanic or Latino	ASD	10
CW20178	194	Plate 4	BR00107339	No	iPSC	PBMC	Male	Hispanic/Latino	Hispanic or Latino	CONTROL	15
CW20182	195	Plate 4	BR00107339	NA	iPSC	PBMC	Male	Hispanic/Latino	Hispanic or Latino	ASD	8
CW20252	196	Plate 4	BR00107339	No	iPSC	PBMC	Female	African American	Not Hispanic or Latino	CONTROL	13
CW30324	197	Plate 4	BR00107339	Yes	iPSC	PBMC	Male	Caucasian	Not Hispanic or Latino	HEART DISEASE	71
CW30369	198	Plate 4	BR00107339	Yes	iPSC	PBMC	Female	Caucasian	Not Hispanic or Latino	HEART DISEASE	26
CW30382	199	Plate 4	BR00107339	No	iPSC	PBMC	Male	Asian	Not Hispanic or Latino	CONTROL	57
CW30420	200	Plate 4	BR00107339	Yes	iPSC	PBMC	Male	Asian	Not Hispanic or Latino	HEART DISEASE	54
CW30428	201	Plate 4	BR00107339	Yes	iPSC	PBMC	Male	African American	Not Hispanic or Latino	HEART DISEASE	28
CW30429	202	Plate 4	BR00107339	Yes	iPSC	PBMC	Female	Caucasian	Not Hispanic or Latino	HEART DISEASE	75
CW30483	203	Plate 4	BR00107339	Yes	iPSC	PBMC	Male	Hispanic/Latino	Hispanic or Latino	HEART DISEASE	54
CW30485	204	Plate 4	BR00107339	Yes	iPSC	PBMC	Male	Caucasian	Not Hispanic or Latino	HEART DISEASE	61
CW40011	205	Plate 4	BR00107339	Yes	iPSC	PBMC	Male	Caucasian	Not Hispanic or Latino	IPF	68
CW40106	206	Plate 4	BR00107339	Yes	iPSC	PBMC	Male	Caucasian	Not Hispanic or Latino	IPF	68
CW40144	207	Plate 4	BR00107339	Yes	iPSC	PBMC	Male	Caucasian	Not Hispanic or Latino	IPF	72
CW40214	208	Plate 4	BR00107339	Yes	iPSC	PBMC	Male	Unknown	Not Hispanic or Latino	IPF	70
CW40219	209	Plate 4	BR00107339	Yes	iPSC	PBMC	Male	Caucasian	Not Hispanic or Latino	IPF	72
CW50147	210	Plate 4	BR00107339	Yes	iPSC	PBMC	Male	Caucasian	Not Hispanic or Latino	ALZHEIMER DISEASE	71
CW60056	211	Plate 4	BR00107339	Yes	iPSC	Fibroblast	Female	Other	Not Hispanic or Latino	ID	11
CW60131	212	Plate 4	BR00107339	Yes	iPSC	Fibroblast	Male	Caucasian	Not Hispanic or Latino	ID	12
CW60441	213	Plate 4	BR00107339	No	iPSC	Fibroblast	Male	Caucasian	Not Hispanic or Latino	CONTROL	30
CW70117	214	Plate 4	BR00107339	Yes	iPSC	PBMC	Female	Caucasian	Not Hispanic or Latino	AMD	90
CW70255	215	Plate 4	BR00107339	Yes	iPSC	PBMC	Male	Caucasian	Not Hispanic or Latino	DIABETIC	63
CW70261	216	Plate 4	BR00107339	Yes	iPSC	PBMC	Female	Filipino	Not Hispanic or Latino	DIABETIC	68

CW70283	217	Plate 4	BR00107339	Yes	iPSC	PBMC	Male	Caucasian	Not Hispanic or Latino	DIABETIC	74
CW70303	218	Plate 4	BR00107339	Yes	iPSC	PBMC	Male	Caucasian	Not Hispanic or Latino	DIABETIC	67
CW80027	219	Plate 4	BR00107339	NA	iPSC	Fibroblast	Female	Caucasian	Not Hispanic or Latino	CONTROL	13
CW90034	220	Plate 4	BR00107339	NA	iPSC	PBMC	Female	Caucasian	Not Hispanic or Latino	ASD	19
CW70031	221	Plate 4	BR00107339	Yes	iPSC	PBMC	Male	Caucasian	Not Hispanic or Latino	DIABETIC	64
CW70140	222	Plate 4	BR00107339	Yes	iPSC	PBMC	Female	Caucasian	Not Hispanic or Latino	AMD	58
CW20020	223	Plate 4	BR00107339	No	iPSC	PBMC	Female	Caucasian	Not Hispanic or Latino	CONTROL	14
CW20042	224	Plate 4	BR00107339	Yes	iPSC	PBMC	Female	Caucasian	Not Hispanic or Latino	ASD	6
CW20106	225	Plate 4	BR00107339	No	iPSC	PBMC	Female	Caucasian	Not Hispanic or Latino	CONTROL	20
CW20133	226	Plate 4	BR00107339	No	iPSC	PBMC	Male	Caucasian	Not Hispanic or Latino	CONTROL	18
CW30212	227	Plate 4	BR00107339	Yes	iPSC	PBMC	Male	Caucasian	Not Hispanic or Latino	HEART DISEASE	49
CW60040	228	Plate 4	BR00107339	No	iPSC	PBMC	Male	Caucasian	Not Hispanic or Latino	CONTROL	5
CW60045	229	Plate 4	BR00107339	No	iPSC	Fibroblast	Female	Other	Not Hispanic or Latino	CONTROL	23
CW60058	230	Plate 4	BR00107339	Yes	iPSC	Fibroblast	Female	Other	Not Hispanic or Latino	ID	37
CW60243	231	Plate 4	BR00107339	Yes	iPSC	Fibroblast	Male	Other	Not Hispanic or Latino	ID	14
CW70356	232	Plate 4	BR00107339	Yes	iPSC	PBMC	Male	Hispanic/Latino	Hispanic or Latino	AMD	31
CW40001	233	Plate 3	BR00107338	Yes	iPSC	PBMC	Male	Caucasian	Not Hispanic or Latino	IPF	68
CW20184	234	Plate 3	BR00107338	Yes	iPSC	PBMC	Male	Caucasian	Not Hispanic or Latino	ASD	24
CW20195	235	Plate 3	BR00107338	Yes	iPSC	PBMC	Female	Caucasian	Not Hispanic or Latino	ASD	24
CW30108	236	Plate 3	BR00107338	Yes	iPSC	PBMC	Male	Caucasian	Not Hispanic or Latino	HEART DISEASE	53
CW30154	237	Plate 3	BR00107338	Yes	iPSC	PBMC	Female	Caucasian	Not Hispanic or Latino	HEART DISEASE	66
CW30190	238	Plate 3	BR00107338	Yes	iPSC	PBMC	Male	Caucasian	Not Hispanic or Latino	HEART DISEASE	63
CW30265	239	Plate 3	BR00107338	Yes	iPSC	PBMC	Female	Caucasian	Not Hispanic or Latino	HEART DISEASE	55
CW30274	240	Plate 3	BR00107338	Yes	iPSC	PBMC	Male	Caucasian	Not Hispanic or Latino	HEART DISEASE	63
CW30350	241	Plate 3	BR00107338	Yes	iPSC	PBMC	Female	Caucasian	Not Hispanic or Latino	HEART DISEASE	12
CW30421	242	Plate 3	BR00107338	Yes	iPSC	PBMC	Female	Caucasian	Not Hispanic or Latino	HEART DISEASE	66
CW30454	243	Plate 3	BR00107338	Yes	iPSC	PBMC	Male	Caucasian	Not Hispanic or Latino	HEART DISEASE	67
CW30484	244	Plate 3	BR00107338	Yes	iPSC	PBMC	Female	Caucasian	Not Hispanic or Latino	HEART DISEASE	23
CW30525	245	Plate 3	BR00107338	Yes	iPSC	PBMC	Female	Caucasian	Not Hispanic or Latino	HEART DISEASE	56
CW40067	246	Plate 3	BR00107338	Yes	iPSC	PBMC	Female	Caucasian	Not Hispanic or Latino	IPF	76

CW40187	247	Plate 3	BR00107338	Yes	iPSC	PBMC	Female	Caucasian	Not Hispanic or Latino	IPF	71
CW40201	248	Plate 3	BR00107338	Yes	iPSC	PBMC	Female	Caucasian	Not Hispanic or Latino	IPF	76
CW50101	249	Plate 3	BR00107338	No	iPSC	PBMC	Female	Caucasian	Not Hispanic or Latino	CONTROL	78
CW50106	250	Plate 3	BR00107338	Yes	iPSC	PBMC	Female	Caucasian	Not Hispanic or Latino	ALZHEIMER DISEASE	67
CW60130	251	Plate 3	BR00107338	Yes	iPSC	PBMC	Female	Caucasian	Not Hispanic or Latino	EPILEPSY	6
CW70004	252	Plate 3	BR00107338	Yes	iPSC	PBMC	Male	Caucasian	Not Hispanic or Latino	DIABETIC	65
CW70016	253	Plate 3	BR00107338	Yes	iPSC	PBMC	Female	Caucasian	Not Hispanic or Latino	DIABETIC	49
CW70142	254	Plate 3	BR00107338	Yes	iPSC	PBMC	Female	Caucasian	Not Hispanic or Latino	AMD	82
CW70151	255	Plate 3	BR00107338	Yes	iPSC	PBMC	Female	Caucasian	Not Hispanic or Latino	AMD	78
CW70164	256	Plate 3	BR00107338	Yes	iPSC	PBMC	Female	Caucasian	Not Hispanic or Latino	AMD	83
CW70191	257	Plate 3	BR00107338	Yes	iPSC	PBMC	Female	Caucasian	Not Hispanic or Latino	AMD	61
CW70280	258	Plate 3	BR00107338	Yes	iPSC	PBMC	Male	Caucasian	Not Hispanic or Latino	DIABETIC	59
CW70372	259	Plate 3	BR00107338	Yes	iPSC	PBMC	Male	Caucasian	Not Hispanic or Latino	AMD	54
CW50094	260	Plate 3	BR00107338	No	iPSC	PBMC	Male	Caucasian	Not Hispanic or Latino	CONTROL	70
CW70179	261	Plate 3	BR00107338	Yes	iPSC	PBMC	Female	Caucasian	Not Hispanic or Latino	AMD	54
CW50032	262	Plate 3	BR00107338	No	iPSC	PBMC	Male	Caucasian	Not Hispanic or Latino	CONTROL	86
CW70001	263	Plate 3	BR00107338	No	iPSC	PBMC	Male	Caucasian	Not Hispanic or Latino	CONTROL	56
CW60242	264	Plate 3	BR00107338	Yes	iPSC	Fibroblast	Female	Other	Not Hispanic or Latino	ID	16
CW70196	265	Plate 3	BR00107338	Yes	iPSC	PBMC	Female	Caucasian	Not Hispanic or Latino	AMD	79
CW70211	266	Plate 3	BR00107338	Yes	iPSC	PBMC	Female	Caucasian	Not Hispanic or Latino	DIABETIC	62
CW40121	267	Plate 3	BR00107338	Yes	iPSC	PBMC	Male	Caucasian	Not Hispanic or Latino	IPF	68
CW10064	268	Plate 7	cmQTLplate7-7-22-20	Yes	iPSC	Fibroblast	Female	African American	Not Hispanic or Latino	LIVER DISEASE	23
CW10163	269	Plate 7	cmQTLplate7-7-22-20	Yes	iPSC	Fibroblast	Male	African American	Not Hispanic or Latino	LIVER DISEASE	60
CW10202	270	Plate 7	cmQTLplate7-7-22-20	Yes	iPSC	PBMC	Male	Caucasian	Hispanic or Latino	LIVER DISEASE	65
CW20009	271	Plate 7	cmQTLplate7-7-22-20	No	iPSC	PBMC	Female	Caucasian	Not Hispanic or Latino	CONTROL	21
CW20026	272	Plate 7	cmQTLplate7-7-22-20	Yes	iPSC	PBMC	Male	Caucasian	Not Hispanic or Latino	ASD	10
CW20032	273	Plate 7	cmQTLplate7-7-22-20	No	iPSC	PBMC	Male	Asian	Not Hispanic or Latino	CONTROL	12
CW20033	274	Plate 7	cmQTLplate7-7-22-20	No	iPSC	PBMC	Female	Caucasian	Hispanic or Latino	CONTROL	18
CW20049	275	Plate 7	cmQTLplate7-7-22-20	No	iPSC	PBMC	Male	Asian	Not Hispanic or Latino	CONTROL	11
CW30178	276	Plate 7	cmQTLplate7-7-22-20	Yes	iPSC	PBMC	Male	Caucasian	Not Hispanic or Latino	HEART DISEASE	50

CW30196	277	Plate 7	cmQTLplate7-7-22-20	Yes	iPSC	PBMC	Female	Caucasian	Not Hispanic or Latino	HEART DISEASE	48
CW30280	278	Plate 7	cmQTLplate7-7-22-20	Yes	iPSC	PBMC	Male	Caucasian	Not Hispanic or Latino	HEART DISEASE	28
CW30291	279	Plate 7	cmQTLplate7-7-22-20	No	iPSC	PBMC	Female	Caucasian	Not Hispanic or Latino	CONTROL	47
CW30292	280	Plate 7	cmQTLplate7-7-22-20	Yes	iPSC	PBMC	Male	Caucasian	Not Hispanic or Latino	HEART DISEASE	17
CW30293	281	Plate 7	cmQTLplate7-7-22-20	Yes	iPSC	PBMC	Male	Caucasian	Not Hispanic or Latino	HEART DISEASE	19
CW30299	282	Plate 7	cmQTLplate7-7-22-20	Yes	iPSC	PBMC	Female	Caucasian	Not Hispanic or Latino	HEART DISEASE	66
CW30306	283	Plate 7	cmQTLplate7-7-22-20	Yes	iPSC	PBMC	Female	Asian	Not Hispanic or Latino	HEART DISEASE	61
CW30307	284	Plate 7	cmQTLplate7-7-22-20	Yes	iPSC	PBMC	Male	Caucasian	Not Hispanic or Latino	HEART DISEASE	18
CW30358	285	Plate 7	cmQTLplate7-7-22-20	No	iPSC	PBMC	Female	Caucasian	Not Hispanic or Latino	CONTROL	48
CW30383	286	Plate 7	cmQTLplate7-7-22-20	Yes	iPSC	PBMC	Male	Caucasian	Not Hispanic or Latino	HEART DISEASE	17
CW30390	287	Plate 7	cmQTLplate7-7-22-20	Yes	iPSC	PBMC	Male	Caucasian	Not Hispanic or Latino	HEART DISEASE	16
CW30391	288	Plate 7	cmQTLplate7-7-22-20	No	iPSC	PBMC	Female	Caucasian	Not Hispanic or Latino	CONTROL	51
CW40013	289	Plate 7	cmQTLplate7-7-22-20	Yes	iPSC	PBMC	Male	African American	Not Hispanic or Latino	IPF	74
CW40197	290	Plate 7	cmQTLplate7-7-22-20	Yes	iPSC	PBMC	Male	Caucasian	Not Hispanic or Latino	IPF	67
CW40220	291	Plate 7	cmQTLplate7-7-22-20	Yes	iPSC	PBMC	Female	African American	Not Hispanic or Latino	IPF	76
CW40228	292	Plate 7	cmQTLplate7-7-22-20	Yes	iPSC	PBMC	Male	Caucasian	Not Hispanic or Latino	IPF	72
CW50058	293	Plate 7	cmQTLplate7-7-22-20	No	iPSC	PBMC	Male	Caucasian	Not Hispanic or Latino	CONTROL	78
CW60029	294	Plate 7	cmQTLplate7-7-22-20	Yes	iPSC	PBMC	Female	Other	Not Hispanic or Latino	ASD	7
CW60286	295	Plate 7	cmQTLplate7-7-22-20	No	iPSC	Fibroblast	Female	Caucasian	Not Hispanic or Latino	CONTROL	26
CW60288	296	Plate 7	cmQTLplate7-7-22-20	Yes	iPSC	Fibroblast	Female	Caucasian	Not Hispanic or Latino	EPILEPSY	3
CW60291	297	Plate 7	cmQTLplate7-7-22-20	Yes	iPSC	Fibroblast	Male	Caucasian	Not Hispanic or Latino	ID	8
CW60305	298	Plate 7	cmQTLplate7-7-22-20	Yes	iPSC	Fibroblast	Male	Caucasian	Not Hispanic or Latino	ID	5
CW60359	299	Plate 7	cmQTLplate7-7-22-20	No	iPSC	Fibroblast	Female	Caucasian	Not Hispanic or Latino	CONTROL	1
CW60420	300	Plate 7	cmQTLplate7-7-22-20	Yes	iPSC	Fibroblast	Male	Caucasian	Not Hispanic or Latino	ID	2
CW60421	301	Plate 7	cmQTLplate7-7-22-20	No	iPSC	Fibroblast	Male	Caucasian	Not Hispanic or Latino	CONTROL	47
CW60480	302	Plate 7	cmQTLplate7-7-22-20	Yes	iPSC	Fibroblast	Male	Caucasian	Not Hispanic or Latino	ID	1
CW60481	303	Plate 7	cmQTLplate7-7-22-20	No	iPSC	Fibroblast	Male	Caucasian	Not Hispanic or Latino	CONTROL	26
CW70235	304	Plate 7	cmQTLplate7-7-22-20	Yes	iPSC	PBMC	Female	Caucasian	Not Hispanic or Latino	DIABETIC	69
CW70272	305	Plate 7	cmQTLplate7-7-22-20	Yes	iPSC	PBMC	Male	Caucasian	Not Hispanic or Latino	DIABETIC	48
CW20140	306	Plate 7	cmQTLplate7-7-22-20	Yes	iPSC	PBMC	Male	Caucasian	Not Hispanic or Latino	ASD	20

CW10095	307	Plate 7	cmQTLplate7-7-22-20	Yes	iPSC	Fibroblast	Male	African American	Not Hispanic or Latino	LIVER DISEASE	56
CW60026	308	Plate 7	cmQTLplate7-7-22-20	Yes	iPSC	PBMC	Female	Caucasian	Not Hispanic or Latino	CEREBRAL PALSY	17

Table S2. All morphological traits measured in our study.

List of all morphological traits measured in our imaging data which passed QC (n=3418).

Trait
Cells_AreaShape_Area
Cells_AreaShape_Compactness
Cells_AreaShape_Eccentricity
Cells_AreaShape_Extent
Cells_AreaShape_FormFactor
Cells_AreaShape_MajorAxisLength
Cells_AreaShape_MaxFeretDiameter
Cells_AreaShape_MaximumRadius
Cells_AreaShape_MeanRadius
Cells_AreaShape_MedianRadius
Cells_AreaShape_MinFeretDiameter
Cells_AreaShape_MinorAxisLength
Cells_AreaShape_Perimeter
Cells_AreaShape_Solidity
Cells_AreaShape_Zernike_0_0
Cells_AreaShape_Zernike_1_1
Cells_AreaShape_Zernike_2_0
Cells_AreaShape_Zernike_2_2
Cells_AreaShape_Zernike_3_1
Cells_AreaShape_Zernike_3_3
Cells_AreaShape_Zernike_4_0
Cells_AreaShape_Zernike_4_2
Cells_AreaShape_Zernike_4_4
Cells_AreaShape_Zernike_5_1
Cells_AreaShape_Zernike_5_3
Cells_AreaShape_Zernike_5_5
Cells_AreaShape_Zernike_6_0
Cells_AreaShape_Zernike_6_2
Cells_AreaShape_Zernike_6_4
Cells_AreaShape_Zernike_6_6
Cells_AreaShape_Zernike_7_1

Cells_AreaShape_Zernike_7_3
Cells_AreaShape_Zernike_7_5
Cells_AreaShape_Zernike_7_7
Cells_AreaShape_Zernike_8_0
Cells_AreaShape_Zernike_8_2
Cells_AreaShape_Zernike_8_4
Cells_AreaShape_Zernike_8_6
Cells_AreaShape_Zernike_8_8
Cells_AreaShape_Zernike_9_1
Cells_AreaShape_Zernike_9_3
Cells_AreaShape_Zernike_9_5
Cells_AreaShape_Zernike_9_7
Cells_AreaShape_Zernike_9_9
Cells_Granularity_10_AGP
Cells_Granularity_10_Brightfield
Cells_Granularity_10_ER
Cells_Granularity_10_Mito
Cells_Granularity_10_RNA
Cells_Granularity_11_AGP
Cells_Granularity_11_Brightfield
Cells_Granularity_11_ER
Cells_Granularity_11_Mito
Cells_Granularity_11_RNA
Cells_Granularity_12_AGP
Cells_Granularity_12_Brightfield
Cells_Granularity_12_ER
Cells_Granularity_12_Mito
Cells_Granularity_12_RNA
Cells_Granularity_13_AGP
Cells_Granularity_13_Brightfield
Cells_Granularity_13_ER
Cells_Granularity_13_Mito
Cells_Granularity_13_RNA
Cells_Granularity_1_AGP
Cells_Granularity_1_Brightfield
Cells_Granularity_1_ER
Cells_Granularity_1_Mito
Cells_Granularity_1_RNA
Cells_Granularity_2_AGP

Cells_Granularity_2_Brightfield
Cells_Granularity_2_ER
Cells_Granularity_2_Mito
Cells_Granularity_2_RNA
Cells_Granularity_3_AGP
Cells_Granularity_3_Brightfield
Cells_Granularity_3_ER
Cells_Granularity_3_Mito
Cells_Granularity_3_RNA
Cells_Granularity_4_AGP
Cells_Granularity_4_Brightfield
Cells_Granularity_4_ER
Cells_Granularity_4_Mito
Cells_Granularity_4_RNA
Cells_Granularity_5_AGP
Cells_Granularity_5_Brightfield
Cells_Granularity_5_ER
Cells_Granularity_5_Mito
Cells_Granularity_5_RNA
Cells_Granularity_6_AGP
Cells_Granularity_6_Brightfield
Cells_Granularity_6_ER
Cells_Granularity_6_Mito
Cells_Granularity_6_RNA
Cells_Granularity_7_AGP
Cells_Granularity_7_Brightfield
Cells_Granularity_7_ER
Cells_Granularity_7_Mito
Cells_Granularity_7_RNA
Cells_Granularity_8_AGP
Cells_Granularity_8_Brightfield
Cells_Granularity_8_ER
Cells_Granularity_8_Mito
Cells_Granularity_8_RNA
Cells_Granularity_9_AGP
Cells_Granularity_9_Brightfield
Cells_Granularity_9_ER
Cells_Granularity_9_Mito
Cells_Granularity_9_RNA

Cells_Intensity_IntegratedIntensityEdge_AGP
Cells_Intensity_IntegratedIntensityEdge_Brightfield
Cells_Intensity_IntegratedIntensityEdge_DNA
Cells_Intensity_IntegratedIntensityEdge_ER
Cells_Intensity_IntegratedIntensityEdge_Mito
Cells_Intensity_IntegratedIntensityEdge_RNA
Cells_Intensity_IntegratedIntensity_AGP
Cells_Intensity_IntegratedIntensity_Brightfield
Cells_Intensity_IntegratedIntensity_DNA
Cells_Intensity_IntegratedIntensity_ER
Cells_Intensity_IntegratedIntensity_Mito
Cells_Intensity_IntegratedIntensity_RNA
Cells_Intensity_LowerQuartileIntensity_AGP
Cells_Intensity_LowerQuartileIntensity_Brightfield
Cells_Intensity_LowerQuartileIntensity_DNA
Cells_Intensity_LowerQuartileIntensity_ER
Cells_Intensity_LowerQuartileIntensity_Mito
Cells_Intensity_LowerQuartileIntensity_RNA
Cells_Intensity_MADIntensity_AGP
Cells_Intensity_MADIntensity_Brightfield
Cells_Intensity_MADIntensity_DNA
Cells_Intensity_MADIntensity_ER
Cells_Intensity_MADIntensity_Mito
Cells_Intensity_MADIntensity_RNA
Cells_Intensity_MassDisplacement_AGP
Cells_Intensity_MassDisplacement_Brightfield
Cells_Intensity_MassDisplacement_DNA
Cells_Intensity_MassDisplacement_ER
Cells_Intensity_MassDisplacement_Mito
Cells_Intensity_MassDisplacement_RNA
Cells_Intensity_MaxIntensityEdge_AGP
Cells_Intensity_MaxIntensityEdge_Brightfield
Cells_Intensity_MaxIntensityEdge_DNA
Cells_Intensity_MaxIntensityEdge_ER
Cells_Intensity_MaxIntensityEdge_Mito
Cells_Intensity_MaxIntensityEdge_RNA
Cells_Intensity_MaxIntensity_AGP
Cells_Intensity_MaxIntensity_Brightfield
Cells_Intensity_MaxIntensity_DNA

Cells_Intensity_MaxIntensity_ER
Cells_Intensity_MaxIntensity_Mito
Cells_Intensity_MaxIntensity_RNA
Cells_Intensity_MeanIntensityEdge_AGP
Cells_Intensity_MeanIntensityEdge_Brightfield
Cells_Intensity_MeanIntensityEdge_DNA
Cells_Intensity_MeanIntensityEdge_ER
Cells_Intensity_MeanIntensityEdge_Mito
Cells_Intensity_MeanIntensityEdge_RNA
Cells_Intensity_MeanIntensity_AGP
Cells_Intensity_MeanIntensity_Brightfield
Cells_Intensity_MeanIntensity_DNA
Cells_Intensity_MeanIntensity_ER
Cells_Intensity_MeanIntensity_Mito
Cells_Intensity_MeanIntensity_RNA
Cells_Intensity_MedianIntensity_AGP
Cells_Intensity_MedianIntensity_Brightfield
Cells_Intensity_MedianIntensity_DNA
Cells_Intensity_MedianIntensity_ER
Cells_Intensity_MedianIntensity_Mito
Cells_Intensity_MedianIntensity_RNA
Cells_Intensity_MinIntensityEdge_AGP
Cells_Intensity_MinIntensityEdge_Brightfield
Cells_Intensity_MinIntensityEdge_DNA
Cells_Intensity_MinIntensityEdge_ER
Cells_Intensity_MinIntensityEdge_Mito
Cells_Intensity_MinIntensityEdge_RNA
Cells_Intensity_MinIntensity_AGP
Cells_Intensity_MinIntensity_Brightfield
Cells_Intensity_MinIntensity_DNA
Cells_Intensity_MinIntensity_ER
Cells_Intensity_MinIntensity_Mito
Cells_Intensity_MinIntensity_RNA
Cells_Intensity_StdIntensityEdge_AGP
Cells_Intensity_StdIntensityEdge_Brightfield
Cells_Intensity_StdIntensityEdge_DNA
Cells_Intensity_StdIntensityEdge_ER
Cells_Intensity_StdIntensityEdge_Mito
Cells_Intensity_StdIntensityEdge_RNA

Cells_Intensity_StdIntensity_AGP
Cells_Intensity_StdIntensity_Brightfield
Cells_Intensity_StdIntensity_DNA
Cells_Intensity_StdIntensity_ER
Cells_Intensity_StdIntensity_Mito
Cells_Intensity_StdIntensity_RNA
Cells_Intensity_UpperQuartileIntensity_AGP
Cells_Intensity_UpperQuartileIntensity_Brightfield
Cells_Intensity_UpperQuartileIntensity_DNA
Cells_Intensity_UpperQuartileIntensity_ER
Cells_Intensity_UpperQuartileIntensity_Mito
Cells_Intensity_UpperQuartileIntensity_RNA
Cells_RadialDistribution_FracAtD_AGP_1of4
Cells_RadialDistribution_FracAtD_AGP_2of4
Cells_RadialDistribution_FracAtD_AGP_3of4
Cells_RadialDistribution_FracAtD_AGP_4of4
Cells_RadialDistribution_FracAtD_Brightfield_1of4
Cells_RadialDistribution_FracAtD_Brightfield_2of4
Cells_RadialDistribution_FracAtD_Brightfield_3of4
Cells_RadialDistribution_FracAtD_Brightfield_4of4
Cells_RadialDistribution_FracAtD_DNA_1of4
Cells_RadialDistribution_FracAtD_DNA_2of4
Cells_RadialDistribution_FracAtD_DNA_3of4
Cells_RadialDistribution_FracAtD_DNA_4of4
Cells_RadialDistribution_FracAtD_ER_1of4
Cells_RadialDistribution_FracAtD_ER_2of4
Cells_RadialDistribution_FracAtD_ER_3of4
Cells_RadialDistribution_FracAtD_ER_4of4
Cells_RadialDistribution_FracAtD_Mito_1of4
Cells_RadialDistribution_FracAtD_Mito_2of4
Cells_RadialDistribution_FracAtD_Mito_3of4
Cells_RadialDistribution_FracAtD_Mito_4of4
Cells_RadialDistribution_FracAtD_RNA_1of4
Cells_RadialDistribution_FracAtD_RNA_2of4
Cells_RadialDistribution_FracAtD_RNA_3of4
Cells_RadialDistribution_FracAtD_RNA_4of4
Cells_RadialDistribution_MeanFrac_AGP_1of4
Cells_RadialDistribution_MeanFrac_AGP_2of4
Cells_RadialDistribution_MeanFrac_AGP_3of4

Cells_RadialDistribution_MeanFrac_AGP_4of4
Cells_RadialDistribution_MeanFrac_Brightfield_1of4
Cells_RadialDistribution_MeanFrac_Brightfield_2of4
Cells_RadialDistribution_MeanFrac_Brightfield_3of4
Cells_RadialDistribution_MeanFrac_Brightfield_4of4
Cells_RadialDistribution_MeanFrac_DNA_1of4
Cells_RadialDistribution_MeanFrac_DNA_2of4
Cells_RadialDistribution_MeanFrac_DNA_3of4
Cells_RadialDistribution_MeanFrac_DNA_4of4
Cells_RadialDistribution_MeanFrac_ER_1of4
Cells_RadialDistribution_MeanFrac_ER_2of4
Cells_RadialDistribution_MeanFrac_ER_3of4
Cells_RadialDistribution_MeanFrac_ER_4of4
Cells_RadialDistribution_MeanFrac_Mito_1of4
Cells_RadialDistribution_MeanFrac_Mito_2of4
Cells_RadialDistribution_MeanFrac_Mito_3of4
Cells_RadialDistribution_MeanFrac_Mito_4of4
Cells_RadialDistribution_MeanFrac_RNA_1of4
Cells_RadialDistribution_MeanFrac_RNA_2of4
Cells_RadialDistribution_MeanFrac_RNA_3of4
Cells_RadialDistribution_MeanFrac_RNA_4of4
Cells_RadialDistribution_RadialCV_AGP_1of4
Cells_RadialDistribution_RadialCV_AGP_2of4
Cells_RadialDistribution_RadialCV_AGP_3of4
Cells_RadialDistribution_RadialCV_AGP_4of4
Cells_RadialDistribution_RadialCV_Brightfield_1of4
Cells_RadialDistribution_RadialCV_Brightfield_2of4
Cells_RadialDistribution_RadialCV_Brightfield_3of4
Cells_RadialDistribution_RadialCV_Brightfield_4of4
Cells_RadialDistribution_RadialCV_DNA_1of4
Cells_RadialDistribution_RadialCV_DNA_2of4
Cells_RadialDistribution_RadialCV_DNA_3of4
Cells_RadialDistribution_RadialCV_DNA_4of4
Cells_RadialDistribution_RadialCV_ER_1of4
Cells_RadialDistribution_RadialCV_ER_2of4
Cells_RadialDistribution_RadialCV_ER_3of4
Cells_RadialDistribution_RadialCV_ER_4of4
Cells_RadialDistribution_RadialCV_Mito_1of4
Cells_RadialDistribution_RadialCV_Mito_2of4

Cells_RadialDistribution_RadialCV_Mito_3of4
Cells_RadialDistribution_RadialCV_Mito_4of4
Cells_RadialDistribution_RadialCV_RNA_1of4
Cells_RadialDistribution_RadialCV_RNA_2of4
Cells_RadialDistribution_RadialCV_RNA_3of4
Cells_RadialDistribution_RadialCV_RNA_4of4
Cells_Texture_AngularSecondMoment_AGP_10_00
Cells_Texture_AngularSecondMoment_AGP_10_01
Cells_Texture_AngularSecondMoment_AGP_10_02
Cells_Texture_AngularSecondMoment_AGP_10_03
Cells_Texture_AngularSecondMoment_AGP_20_00
Cells_Texture_AngularSecondMoment_AGP_20_01
Cells_Texture_AngularSecondMoment_AGP_20_02
Cells_Texture_AngularSecondMoment_AGP_20_03
Cells_Texture_AngularSecondMoment_AGP_5_00
Cells_Texture_AngularSecondMoment_AGP_5_01
Cells_Texture_AngularSecondMoment_AGP_5_02
Cells_Texture_AngularSecondMoment_AGP_5_03
Cells_Texture_AngularSecondMoment_Brightfield_10_00
Cells_Texture_AngularSecondMoment_Brightfield_10_01
Cells_Texture_AngularSecondMoment_Brightfield_10_02
Cells_Texture_AngularSecondMoment_Brightfield_10_03
Cells_Texture_AngularSecondMoment_Brightfield_20_00
Cells_Texture_AngularSecondMoment_Brightfield_20_01
Cells_Texture_AngularSecondMoment_Brightfield_20_02
Cells_Texture_AngularSecondMoment_Brightfield_20_03
Cells_Texture_AngularSecondMoment_Brightfield_5_00
Cells_Texture_AngularSecondMoment_Brightfield_5_01
Cells_Texture_AngularSecondMoment_Brightfield_5_02
Cells_Texture_AngularSecondMoment_Brightfield_5_03
Cells_Texture_AngularSecondMoment_DNA_10_00
Cells_Texture_AngularSecondMoment_DNA_10_01
Cells_Texture_AngularSecondMoment_DNA_10_02
Cells_Texture_AngularSecondMoment_DNA_10_03
Cells_Texture_AngularSecondMoment_DNA_20_00
Cells_Texture_AngularSecondMoment_DNA_20_01
Cells_Texture_AngularSecondMoment_DNA_20_02
Cells_Texture_AngularSecondMoment_DNA_20_03
Cells_Texture_AngularSecondMoment_DNA_5_00

Cells_Texture_AngularSecondMoment_DNA_5_01
Cells_Texture_AngularSecondMoment_DNA_5_02
Cells_Texture_AngularSecondMoment_DNA_5_03
Cells_Texture_AngularSecondMoment_ER_10_00
Cells_Texture_AngularSecondMoment_ER_10_01
Cells_Texture_AngularSecondMoment_ER_10_02
Cells_Texture_AngularSecondMoment_ER_10_03
Cells_Texture_AngularSecondMoment_ER_20_00
Cells_Texture_AngularSecondMoment_ER_20_01
Cells_Texture_AngularSecondMoment_ER_20_02
Cells_Texture_AngularSecondMoment_ER_20_03
Cells_Texture_AngularSecondMoment_ER_5_00
Cells_Texture_AngularSecondMoment_ER_5_01
Cells_Texture_AngularSecondMoment_ER_5_02
Cells_Texture_AngularSecondMoment_ER_5_03
Cells_Texture_AngularSecondMoment_Mito_10_00
Cells_Texture_AngularSecondMoment_Mito_10_01
Cells_Texture_AngularSecondMoment_Mito_10_02
Cells_Texture_AngularSecondMoment_Mito_10_03
Cells_Texture_AngularSecondMoment_Mito_20_00
Cells_Texture_AngularSecondMoment_Mito_20_01
Cells_Texture_AngularSecondMoment_Mito_20_02
Cells_Texture_AngularSecondMoment_Mito_20_03
Cells_Texture_AngularSecondMoment_Mito_5_00
Cells_Texture_AngularSecondMoment_Mito_5_01
Cells_Texture_AngularSecondMoment_Mito_5_02
Cells_Texture_AngularSecondMoment_Mito_5_03
Cells_Texture_AngularSecondMoment_RNA_10_00
Cells_Texture_AngularSecondMoment_RNA_10_01
Cells_Texture_AngularSecondMoment_RNA_10_02
Cells_Texture_AngularSecondMoment_RNA_10_03
Cells_Texture_AngularSecondMoment_RNA_20_00
Cells_Texture_AngularSecondMoment_RNA_20_01
Cells_Texture_AngularSecondMoment_RNA_20_02
Cells_Texture_AngularSecondMoment_RNA_20_03
Cells_Texture_AngularSecondMoment_RNA_5_00
Cells_Texture_AngularSecondMoment_RNA_5_01
Cells_Texture_AngularSecondMoment_RNA_5_02
Cells_Texture_AngularSecondMoment_RNA_5_03

Cells_Texture_Contrast_AGP_10_00
Cells_Texture_Contrast_AGP_10_01
Cells_Texture_Contrast_AGP_10_02
Cells_Texture_Contrast_AGP_10_03
Cells_Texture_Contrast_AGP_20_00
Cells_Texture_Contrast_AGP_20_01
Cells_Texture_Contrast_AGP_20_02
Cells_Texture_Contrast_AGP_20_03
Cells_Texture_Contrast_AGP_5_00
Cells_Texture_Contrast_AGP_5_01
Cells_Texture_Contrast_AGP_5_02
Cells_Texture_Contrast_AGP_5_03
Cells_Texture_Contrast_Brightfield_10_00
Cells_Texture_Contrast_Brightfield_10_01
Cells_Texture_Contrast_Brightfield_10_02
Cells_Texture_Contrast_Brightfield_10_03
Cells_Texture_Contrast_Brightfield_20_00
Cells_Texture_Contrast_Brightfield_20_01
Cells_Texture_Contrast_Brightfield_20_02
Cells_Texture_Contrast_Brightfield_20_03
Cells_Texture_Contrast_Brightfield_5_00
Cells_Texture_Contrast_Brightfield_5_01
Cells_Texture_Contrast_Brightfield_5_02
Cells_Texture_Contrast_Brightfield_5_03
Cells_Texture_Contrast_DNA_10_00
Cells_Texture_Contrast_DNA_10_01
Cells_Texture_Contrast_DNA_10_02
Cells_Texture_Contrast_DNA_10_03
Cells_Texture_Contrast_DNA_20_00
Cells_Texture_Contrast_DNA_20_01
Cells_Texture_Contrast_DNA_20_02
Cells_Texture_Contrast_DNA_20_03
Cells_Texture_Contrast_DNA_5_00
Cells_Texture_Contrast_DNA_5_01
Cells_Texture_Contrast_DNA_5_02
Cells_Texture_Contrast_DNA_5_03
Cells_Texture_Contrast_ER_10_00
Cells_Texture_Contrast_ER_10_01
Cells_Texture_Contrast_ER_10_02

Cells_Texture_Contrast_ER_10_03
Cells_Texture_Contrast_ER_20_00
Cells_Texture_Contrast_ER_20_01
Cells_Texture_Contrast_ER_20_02
Cells_Texture_Contrast_ER_20_03
Cells_Texture_Contrast_ER_5_00
Cells_Texture_Contrast_ER_5_01
Cells_Texture_Contrast_ER_5_02
Cells_Texture_Contrast_ER_5_03
Cells_Texture_Contrast_Mito_10_00
Cells_Texture_Contrast_Mito_10_01
Cells_Texture_Contrast_Mito_10_02
Cells_Texture_Contrast_Mito_10_03
Cells_Texture_Contrast_Mito_20_00
Cells_Texture_Contrast_Mito_20_01
Cells_Texture_Contrast_Mito_20_02
Cells_Texture_Contrast_Mito_20_03
Cells_Texture_Contrast_Mito_5_00
Cells_Texture_Contrast_Mito_5_01
Cells_Texture_Contrast_Mito_5_02
Cells_Texture_Contrast_Mito_5_03
Cells_Texture_Contrast_RNA_10_00
Cells_Texture_Contrast_RNA_10_01
Cells_Texture_Contrast_RNA_10_02
Cells_Texture_Contrast_RNA_10_03
Cells_Texture_Contrast_RNA_20_00
Cells_Texture_Contrast_RNA_20_01
Cells_Texture_Contrast_RNA_20_02
Cells_Texture_Contrast_RNA_20_03
Cells_Texture_Contrast_RNA_5_00
Cells_Texture_Contrast_RNA_5_01
Cells_Texture_Contrast_RNA_5_02
Cells_Texture_Contrast_RNA_5_03
Cells_Texture_DifferenceEntropy_AGP_10_00
Cells_Texture_DifferenceEntropy_AGP_10_01
Cells_Texture_DifferenceEntropy_AGP_10_02
Cells_Texture_DifferenceEntropy_AGP_10_03
Cells_Texture_DifferenceEntropy_AGP_20_00
Cells_Texture_DifferenceEntropy_AGP_20_01

Cells_Texture_DifferenceEntropy_AGP_20_02
Cells_Texture_DifferenceEntropy_AGP_20_03
Cells_Texture_DifferenceEntropy_AGP_5_00
Cells_Texture_DifferenceEntropy_AGP_5_01
Cells_Texture_DifferenceEntropy_AGP_5_02
Cells_Texture_DifferenceEntropy_AGP_5_03
Cells_Texture_DifferenceEntropy_Brightfield_10_00
Cells_Texture_DifferenceEntropy_Brightfield_10_01
Cells_Texture_DifferenceEntropy_Brightfield_10_02
Cells_Texture_DifferenceEntropy_Brightfield_10_03
Cells_Texture_DifferenceEntropy_Brightfield_20_00
Cells_Texture_DifferenceEntropy_Brightfield_20_01
Cells_Texture_DifferenceEntropy_Brightfield_20_02
Cells_Texture_DifferenceEntropy_Brightfield_20_03
Cells_Texture_DifferenceEntropy_Brightfield_5_00
Cells_Texture_DifferenceEntropy_Brightfield_5_01
Cells_Texture_DifferenceEntropy_Brightfield_5_02
Cells_Texture_DifferenceEntropy_Brightfield_5_03
Cells_Texture_DifferenceEntropy_DNA_10_00
Cells_Texture_DifferenceEntropy_DNA_10_01
Cells_Texture_DifferenceEntropy_DNA_10_02
Cells_Texture_DifferenceEntropy_DNA_10_03
Cells_Texture_DifferenceEntropy_DNA_20_00
Cells_Texture_DifferenceEntropy_DNA_20_01
Cells_Texture_DifferenceEntropy_DNA_20_02
Cells_Texture_DifferenceEntropy_DNA_20_03
Cells_Texture_DifferenceEntropy_DNA_5_00
Cells_Texture_DifferenceEntropy_DNA_5_01
Cells_Texture_DifferenceEntropy_DNA_5_02
Cells_Texture_DifferenceEntropy_DNA_5_03
Cells_Texture_DifferenceEntropy_ER_10_00
Cells_Texture_DifferenceEntropy_ER_10_01
Cells_Texture_DifferenceEntropy_ER_10_02
Cells_Texture_DifferenceEntropy_ER_10_03
Cells_Texture_DifferenceEntropy_ER_20_00
Cells_Texture_DifferenceEntropy_ER_20_01
Cells_Texture_DifferenceEntropy_ER_20_02
Cells_Texture_DifferenceEntropy_ER_20_03
Cells_Texture_DifferenceEntropy_ER_5_00

Cells_Texture_DifferenceEntropy_ER_5_01
Cells_Texture_DifferenceEntropy_ER_5_02
Cells_Texture_DifferenceEntropy_ER_5_03
Cells_Texture_DifferenceEntropy_Mito_10_00
Cells_Texture_DifferenceEntropy_Mito_10_01
Cells_Texture_DifferenceEntropy_Mito_10_02
Cells_Texture_DifferenceEntropy_Mito_10_03
Cells_Texture_DifferenceEntropy_Mito_20_00
Cells_Texture_DifferenceEntropy_Mito_20_01
Cells_Texture_DifferenceEntropy_Mito_20_02
Cells_Texture_DifferenceEntropy_Mito_20_03
Cells_Texture_DifferenceEntropy_Mito_5_00
Cells_Texture_DifferenceEntropy_Mito_5_01
Cells_Texture_DifferenceEntropy_Mito_5_02
Cells_Texture_DifferenceEntropy_Mito_5_03
Cells_Texture_DifferenceEntropy_RNA_10_00
Cells_Texture_DifferenceEntropy_RNA_10_01
Cells_Texture_DifferenceEntropy_RNA_10_02
Cells_Texture_DifferenceEntropy_RNA_10_03
Cells_Texture_DifferenceEntropy_RNA_20_00
Cells_Texture_DifferenceEntropy_RNA_20_01
Cells_Texture_DifferenceEntropy_RNA_20_02
Cells_Texture_DifferenceEntropy_RNA_20_03
Cells_Texture_DifferenceEntropy_RNA_5_00
Cells_Texture_DifferenceEntropy_RNA_5_01
Cells_Texture_DifferenceEntropy_RNA_5_02
Cells_Texture_DifferenceEntropy_RNA_5_03
Cells_Texture_DifferenceVariance_AGP_10_00
Cells_Texture_DifferenceVariance_AGP_10_01
Cells_Texture_DifferenceVariance_AGP_10_02
Cells_Texture_DifferenceVariance_AGP_10_03
Cells_Texture_DifferenceVariance_AGP_20_00
Cells_Texture_DifferenceVariance_AGP_20_01
Cells_Texture_DifferenceVariance_AGP_20_02
Cells_Texture_DifferenceVariance_AGP_20_03
Cells_Texture_DifferenceVariance_AGP_5_00
Cells_Texture_DifferenceVariance_AGP_5_01
Cells_Texture_DifferenceVariance_AGP_5_02
Cells_Texture_DifferenceVariance_AGP_5_03

Cells_Texture_DifferenceVariance_Brightfield_10_00
Cells_Texture_DifferenceVariance_Brightfield_10_01
Cells_Texture_DifferenceVariance_Brightfield_10_02
Cells_Texture_DifferenceVariance_Brightfield_10_03
Cells_Texture_DifferenceVariance_Brightfield_20_00
Cells_Texture_DifferenceVariance_Brightfield_20_01
Cells_Texture_DifferenceVariance_Brightfield_20_02
Cells_Texture_DifferenceVariance_Brightfield_20_03
Cells_Texture_DifferenceVariance_Brightfield_5_00
Cells_Texture_DifferenceVariance_Brightfield_5_01
Cells_Texture_DifferenceVariance_Brightfield_5_02
Cells_Texture_DifferenceVariance_Brightfield_5_03
Cells_Texture_DifferenceVariance_DNA_10_00
Cells_Texture_DifferenceVariance_DNA_10_01
Cells_Texture_DifferenceVariance_DNA_10_02
Cells_Texture_DifferenceVariance_DNA_10_03
Cells_Texture_DifferenceVariance_DNA_20_00
Cells_Texture_DifferenceVariance_DNA_20_01
Cells_Texture_DifferenceVariance_DNA_20_02
Cells_Texture_DifferenceVariance_DNA_20_03
Cells_Texture_DifferenceVariance_DNA_5_00
Cells_Texture_DifferenceVariance_DNA_5_01
Cells_Texture_DifferenceVariance_DNA_5_02
Cells_Texture_DifferenceVariance_DNA_5_03
Cells_Texture_DifferenceVariance_ER_10_00
Cells_Texture_DifferenceVariance_ER_10_01
Cells_Texture_DifferenceVariance_ER_10_02
Cells_Texture_DifferenceVariance_ER_10_03
Cells_Texture_DifferenceVariance_ER_20_00
Cells_Texture_DifferenceVariance_ER_20_01
Cells_Texture_DifferenceVariance_ER_20_02
Cells_Texture_DifferenceVariance_ER_20_03
Cells_Texture_DifferenceVariance_ER_5_00
Cells_Texture_DifferenceVariance_ER_5_01
Cells_Texture_DifferenceVariance_ER_5_02
Cells_Texture_DifferenceVariance_ER_5_03
Cells_Texture_DifferenceVariance_Mito_10_00
Cells_Texture_DifferenceVariance_Mito_10_01
Cells_Texture_DifferenceVariance_Mito_10_02

Cells_Texture_DifferenceVariance_Mito_10_03
Cells_Texture_DifferenceVariance_Mito_20_00
Cells_Texture_DifferenceVariance_Mito_20_01
Cells_Texture_DifferenceVariance_Mito_20_02
Cells_Texture_DifferenceVariance_Mito_20_03
Cells_Texture_DifferenceVariance_Mito_5_00
Cells_Texture_DifferenceVariance_Mito_5_01
Cells_Texture_DifferenceVariance_Mito_5_02
Cells_Texture_DifferenceVariance_Mito_5_03
Cells_Texture_DifferenceVariance_RNA_10_00
Cells_Texture_DifferenceVariance_RNA_10_01
Cells_Texture_DifferenceVariance_RNA_10_02
Cells_Texture_DifferenceVariance_RNA_10_03
Cells_Texture_DifferenceVariance_RNA_20_00
Cells_Texture_DifferenceVariance_RNA_20_01
Cells_Texture_DifferenceVariance_RNA_20_02
Cells_Texture_DifferenceVariance_RNA_20_03
Cells_Texture_DifferenceVariance_RNA_5_00
Cells_Texture_DifferenceVariance_RNA_5_01
Cells_Texture_DifferenceVariance_RNA_5_02
Cells_Texture_DifferenceVariance_RNA_5_03
Cells_Texture_Entropy_AGP_10_00
Cells_Texture_Entropy_AGP_10_01
Cells_Texture_Entropy_AGP_10_02
Cells_Texture_Entropy_AGP_10_03
Cells_Texture_Entropy_AGP_20_00
Cells_Texture_Entropy_AGP_20_01
Cells_Texture_Entropy_AGP_20_02
Cells_Texture_Entropy_AGP_20_03
Cells_Texture_Entropy_AGP_5_00
Cells_Texture_Entropy_AGP_5_01
Cells_Texture_Entropy_AGP_5_02
Cells_Texture_Entropy_AGP_5_03
Cells_Texture_Entropy_Brightfield_10_00
Cells_Texture_Entropy_Brightfield_10_01
Cells_Texture_Entropy_Brightfield_10_02
Cells_Texture_Entropy_Brightfield_10_03
Cells_Texture_Entropy_Brightfield_20_00
Cells_Texture_Entropy_Brightfield_20_01

Cells_Texture_Entropy_Brightfield_20_02
Cells_Texture_Entropy_Brightfield_20_03
Cells_Texture_Entropy_Brightfield_5_00
Cells_Texture_Entropy_Brightfield_5_01
Cells_Texture_Entropy_Brightfield_5_02
Cells_Texture_Entropy_Brightfield_5_03
Cells_Texture_Entropy_DNA_10_00
Cells_Texture_Entropy_DNA_10_01
Cells_Texture_Entropy_DNA_10_02
Cells_Texture_Entropy_DNA_10_03
Cells_Texture_Entropy_DNA_20_00
Cells_Texture_Entropy_DNA_20_01
Cells_Texture_Entropy_DNA_20_02
Cells_Texture_Entropy_DNA_20_03
Cells_Texture_Entropy_DNA_5_00
Cells_Texture_Entropy_DNA_5_01
Cells_Texture_Entropy_DNA_5_02
Cells_Texture_Entropy_DNA_5_03
Cells_Texture_Entropy_ER_10_00
Cells_Texture_Entropy_ER_10_01
Cells_Texture_Entropy_ER_10_02
Cells_Texture_Entropy_ER_10_03
Cells_Texture_Entropy_ER_20_00
Cells_Texture_Entropy_ER_20_01
Cells_Texture_Entropy_ER_20_02
Cells_Texture_Entropy_ER_20_03
Cells_Texture_Entropy_ER_5_00
Cells_Texture_Entropy_ER_5_01
Cells_Texture_Entropy_ER_5_02
Cells_Texture_Entropy_ER_5_03
Cells_Texture_Entropy_Mito_10_00
Cells_Texture_Entropy_Mito_10_01
Cells_Texture_Entropy_Mito_10_02
Cells_Texture_Entropy_Mito_10_03
Cells_Texture_Entropy_Mito_20_00
Cells_Texture_Entropy_Mito_20_01
Cells_Texture_Entropy_Mito_20_02
Cells_Texture_Entropy_Mito_20_03
Cells_Texture_Entropy_Mito_5_00

Cells_Texture_Entropy_Mito_5_01
Cells_Texture_Entropy_Mito_5_02
Cells_Texture_Entropy_Mito_5_03
Cells_Texture_Entropy_RNA_10_00
Cells_Texture_Entropy_RNA_10_01
Cells_Texture_Entropy_RNA_10_02
Cells_Texture_Entropy_RNA_10_03
Cells_Texture_Entropy_RNA_20_00
Cells_Texture_Entropy_RNA_20_01
Cells_Texture_Entropy_RNA_20_02
Cells_Texture_Entropy_RNA_20_03
Cells_Texture_Entropy_RNA_5_00
Cells_Texture_Entropy_RNA_5_01
Cells_Texture_Entropy_RNA_5_02
Cells_Texture_Entropy_RNA_5_03
Cells_Texture_InfoMeas1_AGP_10_00
Cells_Texture_InfoMeas1_AGP_10_01
Cells_Texture_InfoMeas1_AGP_10_02
Cells_Texture_InfoMeas1_AGP_10_03
Cells_Texture_InfoMeas1_AGP_20_00
Cells_Texture_InfoMeas1_AGP_20_01
Cells_Texture_InfoMeas1_AGP_20_02
Cells_Texture_InfoMeas1_AGP_20_03
Cells_Texture_InfoMeas1_AGP_5_00
Cells_Texture_InfoMeas1_AGP_5_01
Cells_Texture_InfoMeas1_AGP_5_02
Cells_Texture_InfoMeas1_AGP_5_03
Cells_Texture_InfoMeas1_Brightfield_10_00
Cells_Texture_InfoMeas1_Brightfield_10_01
Cells_Texture_InfoMeas1_Brightfield_10_02
Cells_Texture_InfoMeas1_Brightfield_10_03
Cells_Texture_InfoMeas1_Brightfield_20_00
Cells_Texture_InfoMeas1_Brightfield_20_01
Cells_Texture_InfoMeas1_Brightfield_20_02
Cells_Texture_InfoMeas1_Brightfield_20_03
Cells_Texture_InfoMeas1_Brightfield_5_00
Cells_Texture_InfoMeas1_Brightfield_5_01
Cells_Texture_InfoMeas1_Brightfield_5_02
Cells_Texture_InfoMeas1_Brightfield_5_03

Cells_Texture_InfoMeas1_DNA_10_00
Cells_Texture_InfoMeas1_DNA_10_01
Cells_Texture_InfoMeas1_DNA_10_02
Cells_Texture_InfoMeas1_DNA_10_03
Cells_Texture_InfoMeas1_DNA_20_00
Cells_Texture_InfoMeas1_DNA_20_01
Cells_Texture_InfoMeas1_DNA_20_02
Cells_Texture_InfoMeas1_DNA_20_03
Cells_Texture_InfoMeas1_DNA_5_00
Cells_Texture_InfoMeas1_DNA_5_01
Cells_Texture_InfoMeas1_DNA_5_02
Cells_Texture_InfoMeas1_DNA_5_03
Cells_Texture_InfoMeas1_ER_10_00
Cells_Texture_InfoMeas1_ER_10_01
Cells_Texture_InfoMeas1_ER_10_02
Cells_Texture_InfoMeas1_ER_10_03
Cells_Texture_InfoMeas1_ER_20_00
Cells_Texture_InfoMeas1_ER_20_01
Cells_Texture_InfoMeas1_ER_20_02
Cells_Texture_InfoMeas1_ER_20_03
Cells_Texture_InfoMeas1_ER_5_00
Cells_Texture_InfoMeas1_ER_5_01
Cells_Texture_InfoMeas1_ER_5_02
Cells_Texture_InfoMeas1_ER_5_03
Cells_Texture_InfoMeas1_Mito_10_00
Cells_Texture_InfoMeas1_Mito_10_01
Cells_Texture_InfoMeas1_Mito_10_02
Cells_Texture_InfoMeas1_Mito_10_03
Cells_Texture_InfoMeas1_Mito_20_00
Cells_Texture_InfoMeas1_Mito_20_01
Cells_Texture_InfoMeas1_Mito_20_02
Cells_Texture_InfoMeas1_Mito_20_03
Cells_Texture_InfoMeas1_Mito_5_00
Cells_Texture_InfoMeas1_Mito_5_01
Cells_Texture_InfoMeas1_Mito_5_02
Cells_Texture_InfoMeas1_Mito_5_03
Cells_Texture_InfoMeas1_RNA_10_00
Cells_Texture_InfoMeas1_RNA_10_01
Cells_Texture_InfoMeas1_RNA_10_02

Cells_Texture_InfoMeas1_RNA_10_03
Cells_Texture_InfoMeas1_RNA_20_00
Cells_Texture_InfoMeas1_RNA_20_01
Cells_Texture_InfoMeas1_RNA_20_02
Cells_Texture_InfoMeas1_RNA_20_03
Cells_Texture_InfoMeas1_RNA_5_00
Cells_Texture_InfoMeas1_RNA_5_01
Cells_Texture_InfoMeas1_RNA_5_02
Cells_Texture_InfoMeas1_RNA_5_03
Cells_Texture_InfoMeas2_AGP_10_00
Cells_Texture_InfoMeas2_AGP_10_01
Cells_Texture_InfoMeas2_AGP_10_02
Cells_Texture_InfoMeas2_AGP_10_03
Cells_Texture_InfoMeas2_AGP_20_00
Cells_Texture_InfoMeas2_AGP_20_01
Cells_Texture_InfoMeas2_AGP_20_02
Cells_Texture_InfoMeas2_AGP_20_03
Cells_Texture_InfoMeas2_AGP_5_00
Cells_Texture_InfoMeas2_AGP_5_01
Cells_Texture_InfoMeas2_AGP_5_02
Cells_Texture_InfoMeas2_AGP_5_03
Cells_Texture_InfoMeas2_Brightfield_10_00
Cells_Texture_InfoMeas2_Brightfield_10_01
Cells_Texture_InfoMeas2_Brightfield_10_02
Cells_Texture_InfoMeas2_Brightfield_10_03
Cells_Texture_InfoMeas2_Brightfield_20_00
Cells_Texture_InfoMeas2_Brightfield_20_01
Cells_Texture_InfoMeas2_Brightfield_20_02
Cells_Texture_InfoMeas2_Brightfield_20_03
Cells_Texture_InfoMeas2_Brightfield_5_00
Cells_Texture_InfoMeas2_Brightfield_5_01
Cells_Texture_InfoMeas2_Brightfield_5_02
Cells_Texture_InfoMeas2_Brightfield_5_03
Cells_Texture_InfoMeas2_DNA_10_00
Cells_Texture_InfoMeas2_DNA_10_01
Cells_Texture_InfoMeas2_DNA_10_02
Cells_Texture_InfoMeas2_DNA_10_03
Cells_Texture_InfoMeas2_DNA_20_00
Cells_Texture_InfoMeas2_DNA_20_01

Cells_Texture_InfoMeas2_DNA_20_02
Cells_Texture_InfoMeas2_DNA_20_03
Cells_Texture_InfoMeas2_DNA_5_00
Cells_Texture_InfoMeas2_DNA_5_01
Cells_Texture_InfoMeas2_DNA_5_02
Cells_Texture_InfoMeas2_DNA_5_03
Cells_Texture_InfoMeas2_ER_10_00
Cells_Texture_InfoMeas2_ER_10_01
Cells_Texture_InfoMeas2_ER_10_02
Cells_Texture_InfoMeas2_ER_10_03
Cells_Texture_InfoMeas2_ER_20_00
Cells_Texture_InfoMeas2_ER_20_01
Cells_Texture_InfoMeas2_ER_20_02
Cells_Texture_InfoMeas2_ER_20_03
Cells_Texture_InfoMeas2_ER_5_00
Cells_Texture_InfoMeas2_ER_5_01
Cells_Texture_InfoMeas2_ER_5_02
Cells_Texture_InfoMeas2_ER_5_03
Cells_Texture_InfoMeas2_Mito_10_00
Cells_Texture_InfoMeas2_Mito_10_01
Cells_Texture_InfoMeas2_Mito_10_02
Cells_Texture_InfoMeas2_Mito_10_03
Cells_Texture_InfoMeas2_Mito_20_00
Cells_Texture_InfoMeas2_Mito_20_01
Cells_Texture_InfoMeas2_Mito_20_02
Cells_Texture_InfoMeas2_Mito_20_03
Cells_Texture_InfoMeas2_Mito_5_00
Cells_Texture_InfoMeas2_Mito_5_01
Cells_Texture_InfoMeas2_Mito_5_02
Cells_Texture_InfoMeas2_Mito_5_03
Cells_Texture_InfoMeas2_RNA_10_00
Cells_Texture_InfoMeas2_RNA_10_01
Cells_Texture_InfoMeas2_RNA_10_02
Cells_Texture_InfoMeas2_RNA_10_03
Cells_Texture_InfoMeas2_RNA_20_00
Cells_Texture_InfoMeas2_RNA_20_01
Cells_Texture_InfoMeas2_RNA_20_02
Cells_Texture_InfoMeas2_RNA_20_03
Cells_Texture_InfoMeas2_RNA_5_00

Cells_Texture_InfoMeas2_RNA_5_01
Cells_Texture_InfoMeas2_RNA_5_02
Cells_Texture_InfoMeas2_RNA_5_03
Cells_Texture_InverseDifferenceMoment_AGP_10_00
Cells_Texture_InverseDifferenceMoment_AGP_10_01
Cells_Texture_InverseDifferenceMoment_AGP_10_02
Cells_Texture_InverseDifferenceMoment_AGP_10_03
Cells_Texture_InverseDifferenceMoment_AGP_20_00
Cells_Texture_InverseDifferenceMoment_AGP_20_01
Cells_Texture_InverseDifferenceMoment_AGP_20_02
Cells_Texture_InverseDifferenceMoment_AGP_20_03
Cells_Texture_InverseDifferenceMoment_AGP_5_00
Cells_Texture_InverseDifferenceMoment_AGP_5_01
Cells_Texture_InverseDifferenceMoment_AGP_5_02
Cells_Texture_InverseDifferenceMoment_AGP_5_03
Cells_Texture_InverseDifferenceMoment_Brightfield_10_00
Cells_Texture_InverseDifferenceMoment_Brightfield_10_01
Cells_Texture_InverseDifferenceMoment_Brightfield_10_02
Cells_Texture_InverseDifferenceMoment_Brightfield_10_03
Cells_Texture_InverseDifferenceMoment_Brightfield_20_00
Cells_Texture_InverseDifferenceMoment_Brightfield_20_01
Cells_Texture_InverseDifferenceMoment_Brightfield_20_02
Cells_Texture_InverseDifferenceMoment_Brightfield_20_03
Cells_Texture_InverseDifferenceMoment_Brightfield_5_00
Cells_Texture_InverseDifferenceMoment_Brightfield_5_01
Cells_Texture_InverseDifferenceMoment_Brightfield_5_02
Cells_Texture_InverseDifferenceMoment_Brightfield_5_03
Cells_Texture_InverseDifferenceMoment_DNA_10_00
Cells_Texture_InverseDifferenceMoment_DNA_10_01
Cells_Texture_InverseDifferenceMoment_DNA_10_02
Cells_Texture_InverseDifferenceMoment_DNA_10_03
Cells_Texture_InverseDifferenceMoment_DNA_20_00
Cells_Texture_InverseDifferenceMoment_DNA_20_01
Cells_Texture_InverseDifferenceMoment_DNA_20_02
Cells_Texture_InverseDifferenceMoment_DNA_20_03
Cells_Texture_InverseDifferenceMoment_DNA_5_00
Cells_Texture_InverseDifferenceMoment_DNA_5_01
Cells_Texture_InverseDifferenceMoment_DNA_5_02
Cells_Texture_InverseDifferenceMoment_DNA_5_03

Cells_Texture_InverseDifferenceMoment_ER_10_00
Cells_Texture_InverseDifferenceMoment_ER_10_01
Cells_Texture_InverseDifferenceMoment_ER_10_02
Cells_Texture_InverseDifferenceMoment_ER_10_03
Cells_Texture_InverseDifferenceMoment_ER_20_00
Cells_Texture_InverseDifferenceMoment_ER_20_01
Cells_Texture_InverseDifferenceMoment_ER_20_02
Cells_Texture_InverseDifferenceMoment_ER_20_03
Cells_Texture_InverseDifferenceMoment_ER_5_00
Cells_Texture_InverseDifferenceMoment_ER_5_01
Cells_Texture_InverseDifferenceMoment_ER_5_02
Cells_Texture_InverseDifferenceMoment_ER_5_03
Cells_Texture_InverseDifferenceMoment_Mito_10_00
Cells_Texture_InverseDifferenceMoment_Mito_10_01
Cells_Texture_InverseDifferenceMoment_Mito_10_02
Cells_Texture_InverseDifferenceMoment_Mito_10_03
Cells_Texture_InverseDifferenceMoment_Mito_20_00
Cells_Texture_InverseDifferenceMoment_Mito_20_01
Cells_Texture_InverseDifferenceMoment_Mito_20_02
Cells_Texture_InverseDifferenceMoment_Mito_20_03
Cells_Texture_InverseDifferenceMoment_Mito_5_00
Cells_Texture_InverseDifferenceMoment_Mito_5_01
Cells_Texture_InverseDifferenceMoment_Mito_5_02
Cells_Texture_InverseDifferenceMoment_Mito_5_03
Cells_Texture_InverseDifferenceMoment_RNA_10_00
Cells_Texture_InverseDifferenceMoment_RNA_10_01
Cells_Texture_InverseDifferenceMoment_RNA_10_02
Cells_Texture_InverseDifferenceMoment_RNA_10_03
Cells_Texture_InverseDifferenceMoment_RNA_20_00
Cells_Texture_InverseDifferenceMoment_RNA_20_01
Cells_Texture_InverseDifferenceMoment_RNA_20_02
Cells_Texture_InverseDifferenceMoment_RNA_20_03
Cells_Texture_InverseDifferenceMoment_RNA_5_00
Cells_Texture_InverseDifferenceMoment_RNA_5_01
Cells_Texture_InverseDifferenceMoment_RNA_5_02
Cells_Texture_InverseDifferenceMoment_RNA_5_03
Cells_Texture_SumAverage_AGP_10_00
Cells_Texture_SumAverage_AGP_10_01
Cells_Texture_SumAverage_AGP_10_02

Cells_Texture_SumAverage_AGP_10_03
Cells_Texture_SumAverage_AGP_20_00
Cells_Texture_SumAverage_AGP_20_01
Cells_Texture_SumAverage_AGP_20_02
Cells_Texture_SumAverage_AGP_20_03
Cells_Texture_SumAverage_AGP_5_00
Cells_Texture_SumAverage_AGP_5_01
Cells_Texture_SumAverage_AGP_5_02
Cells_Texture_SumAverage_AGP_5_03
Cells_Texture_SumAverage_Brightfield_10_00
Cells_Texture_SumAverage_Brightfield_10_01
Cells_Texture_SumAverage_Brightfield_10_02
Cells_Texture_SumAverage_Brightfield_10_03
Cells_Texture_SumAverage_Brightfield_20_00
Cells_Texture_SumAverage_Brightfield_20_01
Cells_Texture_SumAverage_Brightfield_20_02
Cells_Texture_SumAverage_Brightfield_20_03
Cells_Texture_SumAverage_Brightfield_5_00
Cells_Texture_SumAverage_Brightfield_5_01
Cells_Texture_SumAverage_Brightfield_5_02
Cells_Texture_SumAverage_Brightfield_5_03
Cells_Texture_SumAverage_DNA_10_00
Cells_Texture_SumAverage_DNA_10_01
Cells_Texture_SumAverage_DNA_10_02
Cells_Texture_SumAverage_DNA_10_03
Cells_Texture_SumAverage_DNA_20_00
Cells_Texture_SumAverage_DNA_20_01
Cells_Texture_SumAverage_DNA_20_02
Cells_Texture_SumAverage_DNA_20_03
Cells_Texture_SumAverage_DNA_5_00
Cells_Texture_SumAverage_DNA_5_01
Cells_Texture_SumAverage_DNA_5_02
Cells_Texture_SumAverage_DNA_5_03
Cells_Texture_SumAverage_ER_10_00
Cells_Texture_SumAverage_ER_10_01
Cells_Texture_SumAverage_ER_10_02
Cells_Texture_SumAverage_ER_10_03
Cells_Texture_SumAverage_ER_20_00
Cells_Texture_SumAverage_ER_20_01

Cells_Texture_SumAverage_ER_20_02
Cells_Texture_SumAverage_ER_20_03
Cells_Texture_SumAverage_ER_5_00
Cells_Texture_SumAverage_ER_5_01
Cells_Texture_SumAverage_ER_5_02
Cells_Texture_SumAverage_ER_5_03
Cells_Texture_SumAverage_Mito_10_00
Cells_Texture_SumAverage_Mito_10_01
Cells_Texture_SumAverage_Mito_10_02
Cells_Texture_SumAverage_Mito_10_03
Cells_Texture_SumAverage_Mito_20_00
Cells_Texture_SumAverage_Mito_20_01
Cells_Texture_SumAverage_Mito_20_02
Cells_Texture_SumAverage_Mito_20_03
Cells_Texture_SumAverage_Mito_5_00
Cells_Texture_SumAverage_Mito_5_01
Cells_Texture_SumAverage_Mito_5_02
Cells_Texture_SumAverage_Mito_5_03
Cells_Texture_SumAverage_RNA_10_00
Cells_Texture_SumAverage_RNA_10_01
Cells_Texture_SumAverage_RNA_10_02
Cells_Texture_SumAverage_RNA_10_03
Cells_Texture_SumAverage_RNA_20_00
Cells_Texture_SumAverage_RNA_20_01
Cells_Texture_SumAverage_RNA_20_02
Cells_Texture_SumAverage_RNA_20_03
Cells_Texture_SumAverage_RNA_5_00
Cells_Texture_SumAverage_RNA_5_01
Cells_Texture_SumAverage_RNA_5_02
Cells_Texture_SumAverage_RNA_5_03
Cells_Texture_SumEntropy_AGP_10_00
Cells_Texture_SumEntropy_AGP_10_01
Cells_Texture_SumEntropy_AGP_10_02
Cells_Texture_SumEntropy_AGP_10_03
Cells_Texture_SumEntropy_AGP_20_00
Cells_Texture_SumEntropy_AGP_20_01
Cells_Texture_SumEntropy_AGP_20_02
Cells_Texture_SumEntropy_AGP_20_03
Cells_Texture_SumEntropy_AGP_5_00

Cells_Texture_SumEntropy_AGP_5_01
Cells_Texture_SumEntropy_AGP_5_02
Cells_Texture_SumEntropy_AGP_5_03
Cells_Texture_SumEntropy_Brightfield_10_00
Cells_Texture_SumEntropy_Brightfield_10_01
Cells_Texture_SumEntropy_Brightfield_10_02
Cells_Texture_SumEntropy_Brightfield_10_03
Cells_Texture_SumEntropy_Brightfield_20_00
Cells_Texture_SumEntropy_Brightfield_20_01
Cells_Texture_SumEntropy_Brightfield_20_02
Cells_Texture_SumEntropy_Brightfield_20_03
Cells_Texture_SumEntropy_Brightfield_5_00
Cells_Texture_SumEntropy_Brightfield_5_01
Cells_Texture_SumEntropy_Brightfield_5_02
Cells_Texture_SumEntropy_Brightfield_5_03
Cells_Texture_SumEntropy_DNA_10_00
Cells_Texture_SumEntropy_DNA_10_01
Cells_Texture_SumEntropy_DNA_10_02
Cells_Texture_SumEntropy_DNA_10_03
Cells_Texture_SumEntropy_DNA_20_00
Cells_Texture_SumEntropy_DNA_20_01
Cells_Texture_SumEntropy_DNA_20_02
Cells_Texture_SumEntropy_DNA_20_03
Cells_Texture_SumEntropy_DNA_5_00
Cells_Texture_SumEntropy_DNA_5_01
Cells_Texture_SumEntropy_DNA_5_02
Cells_Texture_SumEntropy_DNA_5_03
Cells_Texture_SumEntropy_ER_10_00
Cells_Texture_SumEntropy_ER_10_01
Cells_Texture_SumEntropy_ER_10_02
Cells_Texture_SumEntropy_ER_10_03
Cells_Texture_SumEntropy_ER_20_00
Cells_Texture_SumEntropy_ER_20_01
Cells_Texture_SumEntropy_ER_20_02
Cells_Texture_SumEntropy_ER_20_03
Cells_Texture_SumEntropy_ER_5_00
Cells_Texture_SumEntropy_ER_5_01
Cells_Texture_SumEntropy_ER_5_02
Cells_Texture_SumEntropy_ER_5_03

Cells_Texture_SumEntropy_Mito_10_00
Cells_Texture_SumEntropy_Mito_10_01
Cells_Texture_SumEntropy_Mito_10_02
Cells_Texture_SumEntropy_Mito_10_03
Cells_Texture_SumEntropy_Mito_20_00
Cells_Texture_SumEntropy_Mito_20_01
Cells_Texture_SumEntropy_Mito_20_02
Cells_Texture_SumEntropy_Mito_20_03
Cells_Texture_SumEntropy_Mito_5_00
Cells_Texture_SumEntropy_Mito_5_01
Cells_Texture_SumEntropy_Mito_5_02
Cells_Texture_SumEntropy_Mito_5_03
Cells_Texture_SumEntropy_RNA_10_00
Cells_Texture_SumEntropy_RNA_10_01
Cells_Texture_SumEntropy_RNA_10_02
Cells_Texture_SumEntropy_RNA_10_03
Cells_Texture_SumEntropy_RNA_20_00
Cells_Texture_SumEntropy_RNA_20_01
Cells_Texture_SumEntropy_RNA_20_02
Cells_Texture_SumEntropy_RNA_20_03
Cells_Texture_SumEntropy_RNA_5_00
Cells_Texture_SumEntropy_RNA_5_01
Cells_Texture_SumEntropy_RNA_5_02
Cells_Texture_SumEntropy_RNA_5_03
Cells_Texture_SumVariance_AGP_10_00
Cells_Texture_SumVariance_AGP_10_01
Cells_Texture_SumVariance_AGP_10_02
Cells_Texture_SumVariance_AGP_10_03
Cells_Texture_SumVariance_AGP_20_00
Cells_Texture_SumVariance_AGP_20_01
Cells_Texture_SumVariance_AGP_20_02
Cells_Texture_SumVariance_AGP_20_03
Cells_Texture_SumVariance_AGP_5_00
Cells_Texture_SumVariance_AGP_5_01
Cells_Texture_SumVariance_AGP_5_02
Cells_Texture_SumVariance_AGP_5_03
Cells_Texture_SumVariance_Brightfield_10_00
Cells_Texture_SumVariance_Brightfield_10_01
Cells_Texture_SumVariance_Brightfield_10_02

Cells_Texture_SumVariance_Brightfield_10_03
Cells_Texture_SumVariance_Brightfield_20_00
Cells_Texture_SumVariance_Brightfield_20_01
Cells_Texture_SumVariance_Brightfield_20_02
Cells_Texture_SumVariance_Brightfield_20_03
Cells_Texture_SumVariance_Brightfield_5_00
Cells_Texture_SumVariance_Brightfield_5_01
Cells_Texture_SumVariance_Brightfield_5_02
Cells_Texture_SumVariance_Brightfield_5_03
Cells_Texture_SumVariance_DNA_10_00
Cells_Texture_SumVariance_DNA_10_01
Cells_Texture_SumVariance_DNA_10_02
Cells_Texture_SumVariance_DNA_10_03
Cells_Texture_SumVariance_DNA_20_00
Cells_Texture_SumVariance_DNA_20_01
Cells_Texture_SumVariance_DNA_20_02
Cells_Texture_SumVariance_DNA_20_03
Cells_Texture_SumVariance_DNA_5_00
Cells_Texture_SumVariance_DNA_5_01
Cells_Texture_SumVariance_DNA_5_02
Cells_Texture_SumVariance_DNA_5_03
Cells_Texture_SumVariance_ER_10_00
Cells_Texture_SumVariance_ER_10_01
Cells_Texture_SumVariance_ER_10_02
Cells_Texture_SumVariance_ER_10_03
Cells_Texture_SumVariance_ER_20_00
Cells_Texture_SumVariance_ER_20_01
Cells_Texture_SumVariance_ER_20_02
Cells_Texture_SumVariance_ER_20_03
Cells_Texture_SumVariance_ER_5_00
Cells_Texture_SumVariance_ER_5_01
Cells_Texture_SumVariance_ER_5_02
Cells_Texture_SumVariance_ER_5_03
Cells_Texture_SumVariance_Mito_10_00
Cells_Texture_SumVariance_Mito_10_01
Cells_Texture_SumVariance_Mito_10_02
Cells_Texture_SumVariance_Mito_10_03
Cells_Texture_SumVariance_Mito_20_00
Cells_Texture_SumVariance_Mito_20_01

Cells_Texture_SumVariance_Mito_20_02
Cells_Texture_SumVariance_Mito_20_03
Cells_Texture_SumVariance_Mito_5_00
Cells_Texture_SumVariance_Mito_5_01
Cells_Texture_SumVariance_Mito_5_02
Cells_Texture_SumVariance_Mito_5_03
Cells_Texture_SumVariance_RNA_10_00
Cells_Texture_SumVariance_RNA_10_01
Cells_Texture_SumVariance_RNA_10_02
Cells_Texture_SumVariance_RNA_10_03
Cells_Texture_SumVariance_RNA_20_00
Cells_Texture_SumVariance_RNA_20_01
Cells_Texture_SumVariance_RNA_20_02
Cells_Texture_SumVariance_RNA_20_03
Cells_Texture_SumVariance_RNA_5_00
Cells_Texture_SumVariance_RNA_5_01
Cells_Texture_SumVariance_RNA_5_02
Cells_Texture_SumVariance_RNA_5_03
Cells_Texture_Variance_AGP_10_00
Cells_Texture_Variance_AGP_10_01
Cells_Texture_Variance_AGP_10_02
Cells_Texture_Variance_AGP_10_03
Cells_Texture_Variance_AGP_20_00
Cells_Texture_Variance_AGP_20_01
Cells_Texture_Variance_AGP_20_02
Cells_Texture_Variance_AGP_20_03
Cells_Texture_Variance_AGP_5_00
Cells_Texture_Variance_AGP_5_01
Cells_Texture_Variance_AGP_5_02
Cells_Texture_Variance_AGP_5_03
Cells_Texture_Variance_Brightfield_10_00
Cells_Texture_Variance_Brightfield_10_01
Cells_Texture_Variance_Brightfield_10_02
Cells_Texture_Variance_Brightfield_10_03
Cells_Texture_Variance_Brightfield_20_00
Cells_Texture_Variance_Brightfield_20_01
Cells_Texture_Variance_Brightfield_20_02
Cells_Texture_Variance_Brightfield_20_03
Cells_Texture_Variance_Brightfield_5_00

Cells_Texture_Variance_Brightfield_5_01
Cells_Texture_Variance_Brightfield_5_02
Cells_Texture_Variance_Brightfield_5_03
Cells_Texture_Variance_DNA_10_00
Cells_Texture_Variance_DNA_10_01
Cells_Texture_Variance_DNA_10_02
Cells_Texture_Variance_DNA_10_03
Cells_Texture_Variance_DNA_20_00
Cells_Texture_Variance_DNA_20_01
Cells_Texture_Variance_DNA_20_02
Cells_Texture_Variance_DNA_20_03
Cells_Texture_Variance_DNA_5_00
Cells_Texture_Variance_DNA_5_01
Cells_Texture_Variance_DNA_5_02
Cells_Texture_Variance_DNA_5_03
Cells_Texture_Variance_ER_10_00
Cells_Texture_Variance_ER_10_01
Cells_Texture_Variance_ER_10_02
Cells_Texture_Variance_ER_10_03
Cells_Texture_Variance_ER_20_00
Cells_Texture_Variance_ER_20_01
Cells_Texture_Variance_ER_20_02
Cells_Texture_Variance_ER_20_03
Cells_Texture_Variance_ER_5_00
Cells_Texture_Variance_ER_5_01
Cells_Texture_Variance_ER_5_02
Cells_Texture_Variance_ER_5_03
Cells_Texture_Variance_Mito_10_00
Cells_Texture_Variance_Mito_10_01
Cells_Texture_Variance_Mito_10_02
Cells_Texture_Variance_Mito_10_03
Cells_Texture_Variance_Mito_20_00
Cells_Texture_Variance_Mito_20_01
Cells_Texture_Variance_Mito_20_02
Cells_Texture_Variance_Mito_20_03
Cells_Texture_Variance_Mito_5_00
Cells_Texture_Variance_Mito_5_01
Cells_Texture_Variance_Mito_5_02
Cells_Texture_Variance_Mito_5_03

Cells_Texture_Variance_RNA_10_00
Cells_Texture_Variance_RNA_10_01
Cells_Texture_Variance_RNA_10_02
Cells_Texture_Variance_RNA_10_03
Cells_Texture_Variance_RNA_20_00
Cells_Texture_Variance_RNA_20_01
Cells_Texture_Variance_RNA_20_02
Cells_Texture_Variance_RNA_20_03
Cells_Texture_Variance_RNA_5_00
Cells_Texture_Variance_RNA_5_01
Cells_Texture_Variance_RNA_5_02
Cells_Texture_Variance_RNA_5_03
Nuclei_AreaShape_Area
Nuclei_AreaShape_Compactness
Nuclei_AreaShape_Eccentricity
Nuclei_AreaShape_Extent
Nuclei_AreaShape_FormFactor
Nuclei_AreaShape_MajorAxisLength
Nuclei_AreaShape_MaxFeretDiameter
Nuclei_AreaShape_MaximumRadius
Nuclei_AreaShape_MeanRadius
Nuclei_AreaShape_MedianRadius
Nuclei_AreaShape_MinFeretDiameter
Nuclei_AreaShape_MinorAxisLength
Nuclei_AreaShape_Perimeter
Nuclei_AreaShape_Solidity
Nuclei_AreaShape_Zernike_0_0
Nuclei_AreaShape_Zernike_1_1
Nuclei_AreaShape_Zernike_2_0
Nuclei_AreaShape_Zernike_2_2
Nuclei_AreaShape_Zernike_3_1
Nuclei_AreaShape_Zernike_3_3
Nuclei_AreaShape_Zernike_4_0
Nuclei_AreaShape_Zernike_4_2
Nuclei_AreaShape_Zernike_4_4
Nuclei_AreaShape_Zernike_5_1
Nuclei_AreaShape_Zernike_5_3
Nuclei_AreaShape_Zernike_5_5
Nuclei_AreaShape_Zernike_6_0

Nuclei_AreaShape_Zernike_6_2
Nuclei_AreaShape_Zernike_6_4
Nuclei_AreaShape_Zernike_6_6
Nuclei_AreaShape_Zernike_7_1
Nuclei_AreaShape_Zernike_7_3
Nuclei_AreaShape_Zernike_7_5
Nuclei_AreaShape_Zernike_7_7
Nuclei_AreaShape_Zernike_8_0
Nuclei_AreaShape_Zernike_8_2
Nuclei_AreaShape_Zernike_8_4
Nuclei_AreaShape_Zernike_8_6
Nuclei_AreaShape_Zernike_8_8
Nuclei_AreaShape_Zernike_9_1
Nuclei_AreaShape_Zernike_9_3
Nuclei_AreaShape_Zernike_9_5
Nuclei_AreaShape_Zernike_9_7
Nuclei_AreaShape_Zernike_9_9
Nuclei_Granularity_10_AGP
Nuclei_Granularity_10_Brightfield
Nuclei_Granularity_10_DNA
Nuclei_Granularity_10_ER
Nuclei_Granularity_10_Mito
Nuclei_Granularity_10_RNA
Nuclei_Granularity_11_AGP
Nuclei_Granularity_11_Brightfield
Nuclei_Granularity_11_DNA
Nuclei_Granularity_11_ER
Nuclei_Granularity_11_Mito
Nuclei_Granularity_11_RNA
Nuclei_Granularity_12_AGP
Nuclei_Granularity_12_Brightfield
Nuclei_Granularity_12_DNA
Nuclei_Granularity_12_ER
Nuclei_Granularity_12_Mito
Nuclei_Granularity_12_RNA
Nuclei_Granularity_13_AGP
Nuclei_Granularity_13_Brightfield
Nuclei_Granularity_13_DNA
Nuclei_Granularity_13_ER

Nuclei_Granularity_13_Mito
Nuclei_Granularity_13_RNA
Nuclei_Granularity_1_AGP
Nuclei_Granularity_1_Brightfield
Nuclei_Granularity_1_DNA
Nuclei_Granularity_1_ER
Nuclei_Granularity_1_Mito
Nuclei_Granularity_1_RNA
Nuclei_Granularity_2_AGP
Nuclei_Granularity_2_Brightfield
Nuclei_Granularity_2_DNA
Nuclei_Granularity_2_ER
Nuclei_Granularity_2_Mito
Nuclei_Granularity_2_RNA
Nuclei_Granularity_3_AGP
Nuclei_Granularity_3_Brightfield
Nuclei_Granularity_3_DNA
Nuclei_Granularity_3_ER
Nuclei_Granularity_3_Mito
Nuclei_Granularity_3_RNA
Nuclei_Granularity_4_AGP
Nuclei_Granularity_4_Brightfield
Nuclei_Granularity_4_DNA
Nuclei_Granularity_4_ER
Nuclei_Granularity_4_Mito
Nuclei_Granularity_4_RNA
Nuclei_Granularity_5_AGP
Nuclei_Granularity_5_Brightfield
Nuclei_Granularity_5_DNA
Nuclei_Granularity_5_ER
Nuclei_Granularity_5_Mito
Nuclei_Granularity_5_RNA
Nuclei_Granularity_6_AGP
Nuclei_Granularity_6_Brightfield
Nuclei_Granularity_6_DNA
Nuclei_Granularity_6_ER
Nuclei_Granularity_6_Mito
Nuclei_Granularity_6_RNA
Nuclei_Granularity_7_AGP

Nuclei_Granularity_7_Brightfield
Nuclei_Granularity_7_DNA
Nuclei_Granularity_7_ER
Nuclei_Granularity_7_Mito
Nuclei_Granularity_7_RNA
Nuclei_Granularity_8_AGP
Nuclei_Granularity_8_Brightfield
Nuclei_Granularity_8_DNA
Nuclei_Granularity_8_ER
Nuclei_Granularity_8_Mito
Nuclei_Granularity_8_RNA
Nuclei_Granularity_9_AGP
Nuclei_Granularity_9_Brightfield
Nuclei_Granularity_9_DNA
Nuclei_Granularity_9_ER
Nuclei_Granularity_9_Mito
Nuclei_Granularity_9_RNA
Nuclei_Intensity_IntegratedIntensityEdge_AGP
Nuclei_Intensity_IntegratedIntensityEdge_Brightfield
Nuclei_Intensity_IntegratedIntensityEdge_DNA
Nuclei_Intensity_IntegratedIntensityEdge_ER
Nuclei_Intensity_IntegratedIntensityEdge_Mito
Nuclei_Intensity_IntegratedIntensityEdge_RNA
Nuclei_Intensity_IntegratedIntensity_AGP
Nuclei_Intensity_IntegratedIntensity_Brightfield
Nuclei_Intensity_IntegratedIntensity_DNA
Nuclei_Intensity_IntegratedIntensity_ER
Nuclei_Intensity_IntegratedIntensity_Mito
Nuclei_Intensity_IntegratedIntensity_RNA
Nuclei_Intensity_LowerQuartileIntensity_AGP
Nuclei_Intensity_LowerQuartileIntensity_Brightfield
Nuclei_Intensity_LowerQuartileIntensity_DNA
Nuclei_Intensity_LowerQuartileIntensity_ER
Nuclei_Intensity_LowerQuartileIntensity_Mito
Nuclei_Intensity_LowerQuartileIntensity_RNA
Nuclei_Intensity_MADIntensity_AGP
Nuclei_Intensity_MADIntensity_Brightfield
Nuclei_Intensity_MADIntensity_DNA
Nuclei_Intensity_MADIntensity_ER

Nuclei_Intensity_MADIntensity_Mito
Nuclei_Intensity_MADIntensity_RNA
Nuclei_Intensity_MassDisplacement_AGP
Nuclei_Intensity_MassDisplacement_Brightfield
Nuclei_Intensity_MassDisplacement_DNA
Nuclei_Intensity_MassDisplacement_ER
Nuclei_Intensity_MassDisplacement_Mito
Nuclei_Intensity_MassDisplacement_RNA
Nuclei_Intensity_MaxIntensityEdge_AGP
Nuclei_Intensity_MaxIntensityEdge_Brightfield
Nuclei_Intensity_MaxIntensityEdge_DNA
Nuclei_Intensity_MaxIntensityEdge_ER
Nuclei_Intensity_MaxIntensityEdge_Mito
Nuclei_Intensity_MaxIntensityEdge_RNA
Nuclei_Intensity_MaxIntensity_AGP
Nuclei_Intensity_MaxIntensity_Brightfield
Nuclei_Intensity_MaxIntensity_DNA
Nuclei_Intensity_MaxIntensity_ER
Nuclei_Intensity_MaxIntensity_Mito
Nuclei_Intensity_MaxIntensity_RNA
Nuclei_Intensity_MeanIntensityEdge_AGP
Nuclei_Intensity_MeanIntensityEdge_Brightfield
Nuclei_Intensity_MeanIntensityEdge_DNA
Nuclei_Intensity_MeanIntensityEdge_ER
Nuclei_Intensity_MeanIntensityEdge_Mito
Nuclei_Intensity_MeanIntensityEdge_RNA
Nuclei_Intensity_MeanIntensity_AGP
Nuclei_Intensity_MeanIntensity_Brightfield
Nuclei_Intensity_MeanIntensity_DNA
Nuclei_Intensity_MeanIntensity_ER
Nuclei_Intensity_MeanIntensity_Mito
Nuclei_Intensity_MeanIntensity_RNA
Nuclei_Intensity_MedianIntensity_AGP
Nuclei_Intensity_MedianIntensity_Brightfield
Nuclei_Intensity_MedianIntensity_DNA
Nuclei_Intensity_MedianIntensity_ER
Nuclei_Intensity_MedianIntensity_Mito
Nuclei_Intensity_MedianIntensity_RNA
Nuclei_Intensity_MinIntensityEdge_AGP

Nuclei_Intensity_MinIntensityEdge_Brightfield
Nuclei_Intensity_MinIntensityEdge_DNA
Nuclei_Intensity_MinIntensityEdge_ER
Nuclei_Intensity_MinIntensityEdge_Mito
Nuclei_Intensity_MinIntensityEdge_RNA
Nuclei_Intensity_MinIntensity_AGP
Nuclei_Intensity_MinIntensity_Brightfield
Nuclei_Intensity_MinIntensity_DNA
Nuclei_Intensity_MinIntensity_ER
Nuclei_Intensity_MinIntensity_Mito
Nuclei_Intensity_MinIntensity_RNA
Nuclei_Intensity_StdIntensityEdge_AGP
Nuclei_Intensity_StdIntensityEdge_Brightfield
Nuclei_Intensity_StdIntensityEdge_DNA
Nuclei_Intensity_StdIntensityEdge_ER
Nuclei_Intensity_StdIntensityEdge_Mito
Nuclei_Intensity_StdIntensityEdge_RNA
Nuclei_Intensity_StdIntensity_AGP
Nuclei_Intensity_StdIntensity_Brightfield
Nuclei_Intensity_StdIntensity_DNA
Nuclei_Intensity_StdIntensity_ER
Nuclei_Intensity_StdIntensity_Mito
Nuclei_Intensity_StdIntensity_RNA
Nuclei_Intensity_UpperQuartileIntensity_AGP
Nuclei_Intensity_UpperQuartileIntensity_Brightfield
Nuclei_Intensity_UpperQuartileIntensity_DNA
Nuclei_Intensity_UpperQuartileIntensity_ER
Nuclei_Intensity_UpperQuartileIntensity_Mito
Nuclei_Intensity_UpperQuartileIntensity_RNA
Nuclei_RadialDistribution_FracAtD_AGP_1of4
Nuclei_RadialDistribution_FracAtD_AGP_2of4
Nuclei_RadialDistribution_FracAtD_AGP_3of4
Nuclei_RadialDistribution_FracAtD_AGP_4of4
Nuclei_RadialDistribution_FracAtD_Brightfield_1of4
Nuclei_RadialDistribution_FracAtD_Brightfield_2of4
Nuclei_RadialDistribution_FracAtD_Brightfield_3of4
Nuclei_RadialDistribution_FracAtD_Brightfield_4of4
Nuclei_RadialDistribution_FracAtD_DNA_1of4
Nuclei_RadialDistribution_FracAtD_DNA_2of4

Nuclei_RadialDistribution_FracAtD_DNA_3of4
Nuclei_RadialDistribution_FracAtD_DNA_4of4
Nuclei_RadialDistribution_FracAtD_ER_1of4
Nuclei_RadialDistribution_FracAtD_ER_2of4
Nuclei_RadialDistribution_FracAtD_ER_3of4
Nuclei_RadialDistribution_FracAtD_ER_4of4
Nuclei_RadialDistribution_FracAtD_Mito_1of4
Nuclei_RadialDistribution_FracAtD_Mito_2of4
Nuclei_RadialDistribution_FracAtD_Mito_3of4
Nuclei_RadialDistribution_FracAtD_Mito_4of4
Nuclei_RadialDistribution_FracAtD_RNA_1of4
Nuclei_RadialDistribution_FracAtD_RNA_2of4
Nuclei_RadialDistribution_FracAtD_RNA_3of4
Nuclei_RadialDistribution_FracAtD_RNA_4of4
Nuclei_RadialDistribution_MeanFrac_AGP_1of4
Nuclei_RadialDistribution_MeanFrac_AGP_2of4
Nuclei_RadialDistribution_MeanFrac_AGP_3of4
Nuclei_RadialDistribution_MeanFrac_AGP_4of4
Nuclei_RadialDistribution_MeanFrac_Brightfield_1of4
Nuclei_RadialDistribution_MeanFrac_Brightfield_2of4
Nuclei_RadialDistribution_MeanFrac_Brightfield_3of4
Nuclei_RadialDistribution_MeanFrac_Brightfield_4of4
Nuclei_RadialDistribution_MeanFrac_DNA_1of4
Nuclei_RadialDistribution_MeanFrac_DNA_2of4
Nuclei_RadialDistribution_MeanFrac_DNA_3of4
Nuclei_RadialDistribution_MeanFrac_DNA_4of4
Nuclei_RadialDistribution_MeanFrac_ER_1of4
Nuclei_RadialDistribution_MeanFrac_ER_2of4
Nuclei_RadialDistribution_MeanFrac_ER_3of4
Nuclei_RadialDistribution_MeanFrac_ER_4of4
Nuclei_RadialDistribution_MeanFrac_Mito_1of4
Nuclei_RadialDistribution_MeanFrac_Mito_2of4
Nuclei_RadialDistribution_MeanFrac_Mito_3of4
Nuclei_RadialDistribution_MeanFrac_Mito_4of4
Nuclei_RadialDistribution_MeanFrac_RNA_1of4
Nuclei_RadialDistribution_MeanFrac_RNA_2of4
Nuclei_RadialDistribution_MeanFrac_RNA_3of4
Nuclei_RadialDistribution_MeanFrac_RNA_4of4
Nuclei_RadialDistribution_RadialCV_AGP_1of4

Nuclei_RadialDistribution_RadialCV_AGP_2of4
Nuclei_RadialDistribution_RadialCV_AGP_3of4
Nuclei_RadialDistribution_RadialCV_AGP_4of4
Nuclei_RadialDistribution_RadialCV_Brightfield_1of4
Nuclei_RadialDistribution_RadialCV_Brightfield_2of4
Nuclei_RadialDistribution_RadialCV_Brightfield_3of4
Nuclei_RadialDistribution_RadialCV_Brightfield_4of4
Nuclei_RadialDistribution_RadialCV_DNA_1of4
Nuclei_RadialDistribution_RadialCV_DNA_2of4
Nuclei_RadialDistribution_RadialCV_DNA_3of4
Nuclei_RadialDistribution_RadialCV_DNA_4of4
Nuclei_RadialDistribution_RadialCV_ER_1of4
Nuclei_RadialDistribution_RadialCV_ER_2of4
Nuclei_RadialDistribution_RadialCV_ER_3of4
Nuclei_RadialDistribution_RadialCV_ER_4of4
Nuclei_RadialDistribution_RadialCV_Mito_1of4
Nuclei_RadialDistribution_RadialCV_Mito_2of4
Nuclei_RadialDistribution_RadialCV_Mito_3of4
Nuclei_RadialDistribution_RadialCV_Mito_4of4
Nuclei_RadialDistribution_RadialCV_RNA_1of4
Nuclei_RadialDistribution_RadialCV_RNA_2of4
Nuclei_RadialDistribution_RadialCV_RNA_3of4
Nuclei_RadialDistribution_RadialCV_RNA_4of4
Nuclei_Texture_AngularSecondMoment_AGP_10_00
Nuclei_Texture_AngularSecondMoment_AGP_10_01
Nuclei_Texture_AngularSecondMoment_AGP_10_02
Nuclei_Texture_AngularSecondMoment_AGP_10_03
Nuclei_Texture_AngularSecondMoment_AGP_20_00
Nuclei_Texture_AngularSecondMoment_AGP_20_01
Nuclei_Texture_AngularSecondMoment_AGP_20_02
Nuclei_Texture_AngularSecondMoment_AGP_20_03
Nuclei_Texture_AngularSecondMoment_AGP_5_00
Nuclei_Texture_AngularSecondMoment_AGP_5_01
Nuclei_Texture_AngularSecondMoment_AGP_5_02
Nuclei_Texture_AngularSecondMoment_AGP_5_03
Nuclei_Texture_AngularSecondMoment_Brightfield_10_00
Nuclei_Texture_AngularSecondMoment_Brightfield_10_01
Nuclei_Texture_AngularSecondMoment_Brightfield_10_02
Nuclei_Texture_AngularSecondMoment_Brightfield_10_03

Nuclei_Texture_AngularSecondMoment_Brightfield_20_00
Nuclei_Texture_AngularSecondMoment_Brightfield_20_01
Nuclei_Texture_AngularSecondMoment_Brightfield_20_02
Nuclei_Texture_AngularSecondMoment_Brightfield_20_03
Nuclei_Texture_AngularSecondMoment_Brightfield_5_00
Nuclei_Texture_AngularSecondMoment_Brightfield_5_01
Nuclei_Texture_AngularSecondMoment_Brightfield_5_02
Nuclei_Texture_AngularSecondMoment_Brightfield_5_03
Nuclei_Texture_AngularSecondMoment_DNA_10_00
Nuclei_Texture_AngularSecondMoment_DNA_10_01
Nuclei_Texture_AngularSecondMoment_DNA_10_02
Nuclei_Texture_AngularSecondMoment_DNA_10_03
Nuclei_Texture_AngularSecondMoment_DNA_20_00
Nuclei_Texture_AngularSecondMoment_DNA_20_01
Nuclei_Texture_AngularSecondMoment_DNA_20_02
Nuclei_Texture_AngularSecondMoment_DNA_20_03
Nuclei_Texture_AngularSecondMoment_DNA_5_00
Nuclei_Texture_AngularSecondMoment_DNA_5_01
Nuclei_Texture_AngularSecondMoment_DNA_5_02
Nuclei_Texture_AngularSecondMoment_DNA_5_03
Nuclei_Texture_AngularSecondMoment_ER_10_00
Nuclei_Texture_AngularSecondMoment_ER_10_01
Nuclei_Texture_AngularSecondMoment_ER_10_02
Nuclei_Texture_AngularSecondMoment_ER_10_03
Nuclei_Texture_AngularSecondMoment_ER_20_00
Nuclei_Texture_AngularSecondMoment_ER_20_01
Nuclei_Texture_AngularSecondMoment_ER_20_02
Nuclei_Texture_AngularSecondMoment_ER_20_03
Nuclei_Texture_AngularSecondMoment_ER_5_00
Nuclei_Texture_AngularSecondMoment_ER_5_01
Nuclei_Texture_AngularSecondMoment_ER_5_02
Nuclei_Texture_AngularSecondMoment_ER_5_03
Nuclei_Texture_AngularSecondMoment_Mito_10_00
Nuclei_Texture_AngularSecondMoment_Mito_10_01
Nuclei_Texture_AngularSecondMoment_Mito_10_02
Nuclei_Texture_AngularSecondMoment_Mito_10_03
Nuclei_Texture_AngularSecondMoment_Mito_20_00
Nuclei_Texture_AngularSecondMoment_Mito_20_01
Nuclei_Texture_AngularSecondMoment_Mito_20_02

Nuclei_Texture_AngularSecondMoment_Mito_20_03
Nuclei_Texture_AngularSecondMoment_Mito_5_00
Nuclei_Texture_AngularSecondMoment_Mito_5_01
Nuclei_Texture_AngularSecondMoment_Mito_5_02
Nuclei_Texture_AngularSecondMoment_Mito_5_03
Nuclei_Texture_AngularSecondMoment_RNA_10_00
Nuclei_Texture_AngularSecondMoment_RNA_10_01
Nuclei_Texture_AngularSecondMoment_RNA_10_02
Nuclei_Texture_AngularSecondMoment_RNA_10_03
Nuclei_Texture_AngularSecondMoment_RNA_20_00
Nuclei_Texture_AngularSecondMoment_RNA_20_01
Nuclei_Texture_AngularSecondMoment_RNA_20_02
Nuclei_Texture_AngularSecondMoment_RNA_20_03
Nuclei_Texture_AngularSecondMoment_RNA_5_00
Nuclei_Texture_AngularSecondMoment_RNA_5_01
Nuclei_Texture_AngularSecondMoment_RNA_5_02
Nuclei_Texture_AngularSecondMoment_RNA_5_03
Nuclei_Texture_Contrast_AGP_10_00
Nuclei_Texture_Contrast_AGP_10_01
Nuclei_Texture_Contrast_AGP_10_02
Nuclei_Texture_Contrast_AGP_10_03
Nuclei_Texture_Contrast_AGP_20_00
Nuclei_Texture_Contrast_AGP_20_01
Nuclei_Texture_Contrast_AGP_20_02
Nuclei_Texture_Contrast_AGP_20_03
Nuclei_Texture_Contrast_AGP_5_00
Nuclei_Texture_Contrast_AGP_5_01
Nuclei_Texture_Contrast_AGP_5_02
Nuclei_Texture_Contrast_AGP_5_03
Nuclei_Texture_Contrast_Brightfield_10_00
Nuclei_Texture_Contrast_Brightfield_10_01
Nuclei_Texture_Contrast_Brightfield_10_02
Nuclei_Texture_Contrast_Brightfield_10_03
Nuclei_Texture_Contrast_Brightfield_20_00
Nuclei_Texture_Contrast_Brightfield_20_01
Nuclei_Texture_Contrast_Brightfield_20_02
Nuclei_Texture_Contrast_Brightfield_20_03
Nuclei_Texture_Contrast_Brightfield_5_00
Nuclei_Texture_Contrast_Brightfield_5_01

Nuclei_Texture_Contrast_Brightfield_5_02
Nuclei_Texture_Contrast_Brightfield_5_03
Nuclei_Texture_Contrast_DNA_10_00
Nuclei_Texture_Contrast_DNA_10_01
Nuclei_Texture_Contrast_DNA_10_02
Nuclei_Texture_Contrast_DNA_10_03
Nuclei_Texture_Contrast_DNA_20_00
Nuclei_Texture_Contrast_DNA_20_01
Nuclei_Texture_Contrast_DNA_20_02
Nuclei_Texture_Contrast_DNA_20_03
Nuclei_Texture_Contrast_DNA_5_00
Nuclei_Texture_Contrast_DNA_5_01
Nuclei_Texture_Contrast_DNA_5_02
Nuclei_Texture_Contrast_DNA_5_03
Nuclei_Texture_Contrast_ER_10_00
Nuclei_Texture_Contrast_ER_10_01
Nuclei_Texture_Contrast_ER_10_02
Nuclei_Texture_Contrast_ER_10_03
Nuclei_Texture_Contrast_ER_20_00
Nuclei_Texture_Contrast_ER_20_01
Nuclei_Texture_Contrast_ER_20_02
Nuclei_Texture_Contrast_ER_20_03
Nuclei_Texture_Contrast_ER_5_00
Nuclei_Texture_Contrast_ER_5_01
Nuclei_Texture_Contrast_ER_5_02
Nuclei_Texture_Contrast_ER_5_03
Nuclei_Texture_Contrast_Mito_10_00
Nuclei_Texture_Contrast_Mito_10_01
Nuclei_Texture_Contrast_Mito_10_02
Nuclei_Texture_Contrast_Mito_10_03
Nuclei_Texture_Contrast_Mito_20_00
Nuclei_Texture_Contrast_Mito_20_01
Nuclei_Texture_Contrast_Mito_20_02
Nuclei_Texture_Contrast_Mito_20_03
Nuclei_Texture_Contrast_Mito_5_00
Nuclei_Texture_Contrast_Mito_5_01
Nuclei_Texture_Contrast_Mito_5_02
Nuclei_Texture_Contrast_Mito_5_03
Nuclei_Texture_Contrast_RNA_10_00

Nuclei_Texture_Contrast_RNA_10_01
Nuclei_Texture_Contrast_RNA_10_02
Nuclei_Texture_Contrast_RNA_10_03
Nuclei_Texture_Contrast_RNA_20_00
Nuclei_Texture_Contrast_RNA_20_01
Nuclei_Texture_Contrast_RNA_20_02
Nuclei_Texture_Contrast_RNA_20_03
Nuclei_Texture_Contrast_RNA_5_00
Nuclei_Texture_Contrast_RNA_5_01
Nuclei_Texture_Contrast_RNA_5_02
Nuclei_Texture_Contrast_RNA_5_03
Nuclei_Texture_DifferenceEntropy_AGP_10_00
Nuclei_Texture_DifferenceEntropy_AGP_10_01
Nuclei_Texture_DifferenceEntropy_AGP_10_02
Nuclei_Texture_DifferenceEntropy_AGP_10_03
Nuclei_Texture_DifferenceEntropy_AGP_20_00
Nuclei_Texture_DifferenceEntropy_AGP_20_01
Nuclei_Texture_DifferenceEntropy_AGP_20_02
Nuclei_Texture_DifferenceEntropy_AGP_20_03
Nuclei_Texture_DifferenceEntropy_AGP_5_00
Nuclei_Texture_DifferenceEntropy_AGP_5_01
Nuclei_Texture_DifferenceEntropy_AGP_5_02
Nuclei_Texture_DifferenceEntropy_AGP_5_03
Nuclei_Texture_DifferenceEntropy_Brightfield_10_00
Nuclei_Texture_DifferenceEntropy_Brightfield_10_01
Nuclei_Texture_DifferenceEntropy_Brightfield_10_02
Nuclei_Texture_DifferenceEntropy_Brightfield_10_03
Nuclei_Texture_DifferenceEntropy_Brightfield_20_00
Nuclei_Texture_DifferenceEntropy_Brightfield_20_01
Nuclei_Texture_DifferenceEntropy_Brightfield_20_02
Nuclei_Texture_DifferenceEntropy_Brightfield_20_03
Nuclei_Texture_DifferenceEntropy_Brightfield_5_00
Nuclei_Texture_DifferenceEntropy_Brightfield_5_01
Nuclei_Texture_DifferenceEntropy_Brightfield_5_02
Nuclei_Texture_DifferenceEntropy_Brightfield_5_03
Nuclei_Texture_DifferenceEntropy_DNA_10_00
Nuclei_Texture_DifferenceEntropy_DNA_10_01
Nuclei_Texture_DifferenceEntropy_DNA_10_02
Nuclei_Texture_DifferenceEntropy_DNA_10_03

Nuclei_Texture_DifferenceEntropy_DNA_20_00
Nuclei_Texture_DifferenceEntropy_DNA_20_01
Nuclei_Texture_DifferenceEntropy_DNA_20_02
Nuclei_Texture_DifferenceEntropy_DNA_20_03
Nuclei_Texture_DifferenceEntropy_DNA_5_00
Nuclei_Texture_DifferenceEntropy_DNA_5_01
Nuclei_Texture_DifferenceEntropy_DNA_5_02
Nuclei_Texture_DifferenceEntropy_DNA_5_03
Nuclei_Texture_DifferenceEntropy_ER_10_00
Nuclei_Texture_DifferenceEntropy_ER_10_01
Nuclei_Texture_DifferenceEntropy_ER_10_02
Nuclei_Texture_DifferenceEntropy_ER_10_03
Nuclei_Texture_DifferenceEntropy_ER_20_00
Nuclei_Texture_DifferenceEntropy_ER_20_01
Nuclei_Texture_DifferenceEntropy_ER_20_02
Nuclei_Texture_DifferenceEntropy_ER_20_03
Nuclei_Texture_DifferenceEntropy_ER_5_00
Nuclei_Texture_DifferenceEntropy_ER_5_01
Nuclei_Texture_DifferenceEntropy_ER_5_02
Nuclei_Texture_DifferenceEntropy_ER_5_03
Nuclei_Texture_DifferenceEntropy_Mito_10_00
Nuclei_Texture_DifferenceEntropy_Mito_10_01
Nuclei_Texture_DifferenceEntropy_Mito_10_02
Nuclei_Texture_DifferenceEntropy_Mito_10_03
Nuclei_Texture_DifferenceEntropy_Mito_20_00
Nuclei_Texture_DifferenceEntropy_Mito_20_01
Nuclei_Texture_DifferenceEntropy_Mito_20_02
Nuclei_Texture_DifferenceEntropy_Mito_20_03
Nuclei_Texture_DifferenceEntropy_Mito_5_00
Nuclei_Texture_DifferenceEntropy_Mito_5_01
Nuclei_Texture_DifferenceEntropy_Mito_5_02
Nuclei_Texture_DifferenceEntropy_Mito_5_03
Nuclei_Texture_DifferenceEntropy_RNA_10_00
Nuclei_Texture_DifferenceEntropy_RNA_10_01
Nuclei_Texture_DifferenceEntropy_RNA_10_02
Nuclei_Texture_DifferenceEntropy_RNA_10_03
Nuclei_Texture_DifferenceEntropy_RNA_20_00
Nuclei_Texture_DifferenceEntropy_RNA_20_01
Nuclei_Texture_DifferenceEntropy_RNA_20_02

Nuclei_Texture_DifferenceEntropy_RNA_20_03
Nuclei_Texture_DifferenceEntropy_RNA_5_00
Nuclei_Texture_DifferenceEntropy_RNA_5_01
Nuclei_Texture_DifferenceEntropy_RNA_5_02
Nuclei_Texture_DifferenceEntropy_RNA_5_03
Nuclei_Texture_DifferenceVariance_AGP_10_00
Nuclei_Texture_DifferenceVariance_AGP_10_01
Nuclei_Texture_DifferenceVariance_AGP_10_02
Nuclei_Texture_DifferenceVariance_AGP_10_03
Nuclei_Texture_DifferenceVariance_AGP_20_00
Nuclei_Texture_DifferenceVariance_AGP_20_01
Nuclei_Texture_DifferenceVariance_AGP_20_02
Nuclei_Texture_DifferenceVariance_AGP_20_03
Nuclei_Texture_DifferenceVariance_AGP_5_00
Nuclei_Texture_DifferenceVariance_AGP_5_01
Nuclei_Texture_DifferenceVariance_AGP_5_02
Nuclei_Texture_DifferenceVariance_AGP_5_03
Nuclei_Texture_DifferenceVariance_Brightfield_10_00
Nuclei_Texture_DifferenceVariance_Brightfield_10_01
Nuclei_Texture_DifferenceVariance_Brightfield_10_02
Nuclei_Texture_DifferenceVariance_Brightfield_10_03
Nuclei_Texture_DifferenceVariance_Brightfield_20_00
Nuclei_Texture_DifferenceVariance_Brightfield_20_01
Nuclei_Texture_DifferenceVariance_Brightfield_20_02
Nuclei_Texture_DifferenceVariance_Brightfield_20_03
Nuclei_Texture_DifferenceVariance_Brightfield_5_00
Nuclei_Texture_DifferenceVariance_Brightfield_5_01
Nuclei_Texture_DifferenceVariance_Brightfield_5_02
Nuclei_Texture_DifferenceVariance_Brightfield_5_03
Nuclei_Texture_DifferenceVariance_DNA_10_00
Nuclei_Texture_DifferenceVariance_DNA_10_01
Nuclei_Texture_DifferenceVariance_DNA_10_02
Nuclei_Texture_DifferenceVariance_DNA_10_03
Nuclei_Texture_DifferenceVariance_DNA_20_00
Nuclei_Texture_DifferenceVariance_DNA_20_01
Nuclei_Texture_DifferenceVariance_DNA_20_02
Nuclei_Texture_DifferenceVariance_DNA_20_03
Nuclei_Texture_DifferenceVariance_DNA_5_00
Nuclei_Texture_DifferenceVariance_DNA_5_01

Nuclei_Texture_DifferenceVariance_DNA_5_02
Nuclei_Texture_DifferenceVariance_DNA_5_03
Nuclei_Texture_DifferenceVariance_ER_10_00
Nuclei_Texture_DifferenceVariance_ER_10_01
Nuclei_Texture_DifferenceVariance_ER_10_02
Nuclei_Texture_DifferenceVariance_ER_10_03
Nuclei_Texture_DifferenceVariance_ER_20_00
Nuclei_Texture_DifferenceVariance_ER_20_01
Nuclei_Texture_DifferenceVariance_ER_20_02
Nuclei_Texture_DifferenceVariance_ER_20_03
Nuclei_Texture_DifferenceVariance_ER_5_00
Nuclei_Texture_DifferenceVariance_ER_5_01
Nuclei_Texture_DifferenceVariance_ER_5_02
Nuclei_Texture_DifferenceVariance_ER_5_03
Nuclei_Texture_DifferenceVariance_Mito_10_00
Nuclei_Texture_DifferenceVariance_Mito_10_01
Nuclei_Texture_DifferenceVariance_Mito_10_02
Nuclei_Texture_DifferenceVariance_Mito_10_03
Nuclei_Texture_DifferenceVariance_Mito_20_00
Nuclei_Texture_DifferenceVariance_Mito_20_01
Nuclei_Texture_DifferenceVariance_Mito_20_02
Nuclei_Texture_DifferenceVariance_Mito_20_03
Nuclei_Texture_DifferenceVariance_Mito_5_00
Nuclei_Texture_DifferenceVariance_Mito_5_01
Nuclei_Texture_DifferenceVariance_Mito_5_02
Nuclei_Texture_DifferenceVariance_Mito_5_03
Nuclei_Texture_DifferenceVariance_RNA_10_00
Nuclei_Texture_DifferenceVariance_RNA_10_01
Nuclei_Texture_DifferenceVariance_RNA_10_02
Nuclei_Texture_DifferenceVariance_RNA_10_03
Nuclei_Texture_DifferenceVariance_RNA_20_00
Nuclei_Texture_DifferenceVariance_RNA_20_01
Nuclei_Texture_DifferenceVariance_RNA_20_02
Nuclei_Texture_DifferenceVariance_RNA_20_03
Nuclei_Texture_DifferenceVariance_RNA_5_00
Nuclei_Texture_DifferenceVariance_RNA_5_01
Nuclei_Texture_DifferenceVariance_RNA_5_02
Nuclei_Texture_DifferenceVariance_RNA_5_03
Nuclei_Texture_Entropy_AGP_10_00

Nuclei_Texture_Entropy_AGP_10_01
Nuclei_Texture_Entropy_AGP_10_02
Nuclei_Texture_Entropy_AGP_10_03
Nuclei_Texture_Entropy_AGP_20_00
Nuclei_Texture_Entropy_AGP_20_01
Nuclei_Texture_Entropy_AGP_20_02
Nuclei_Texture_Entropy_AGP_20_03
Nuclei_Texture_Entropy_AGP_5_00
Nuclei_Texture_Entropy_AGP_5_01
Nuclei_Texture_Entropy_AGP_5_02
Nuclei_Texture_Entropy_AGP_5_03
Nuclei_Texture_Entropy_Brightfield_10_00
Nuclei_Texture_Entropy_Brightfield_10_01
Nuclei_Texture_Entropy_Brightfield_10_02
Nuclei_Texture_Entropy_Brightfield_10_03
Nuclei_Texture_Entropy_Brightfield_20_00
Nuclei_Texture_Entropy_Brightfield_20_01
Nuclei_Texture_Entropy_Brightfield_20_02
Nuclei_Texture_Entropy_Brightfield_20_03
Nuclei_Texture_Entropy_Brightfield_5_00
Nuclei_Texture_Entropy_Brightfield_5_01
Nuclei_Texture_Entropy_Brightfield_5_02
Nuclei_Texture_Entropy_Brightfield_5_03
Nuclei_Texture_Entropy_DNA_10_00
Nuclei_Texture_Entropy_DNA_10_01
Nuclei_Texture_Entropy_DNA_10_02
Nuclei_Texture_Entropy_DNA_10_03
Nuclei_Texture_Entropy_DNA_20_00
Nuclei_Texture_Entropy_DNA_20_01
Nuclei_Texture_Entropy_DNA_20_02
Nuclei_Texture_Entropy_DNA_20_03
Nuclei_Texture_Entropy_DNA_5_00
Nuclei_Texture_Entropy_DNA_5_01
Nuclei_Texture_Entropy_DNA_5_02
Nuclei_Texture_Entropy_DNA_5_03
Nuclei_Texture_Entropy_ER_10_00
Nuclei_Texture_Entropy_ER_10_01
Nuclei_Texture_Entropy_ER_10_02
Nuclei_Texture_Entropy_ER_10_03

Nuclei_Texture_Entropy_ER_20_00
Nuclei_Texture_Entropy_ER_20_01
Nuclei_Texture_Entropy_ER_20_02
Nuclei_Texture_Entropy_ER_20_03
Nuclei_Texture_Entropy_ER_5_00
Nuclei_Texture_Entropy_ER_5_01
Nuclei_Texture_Entropy_ER_5_02
Nuclei_Texture_Entropy_ER_5_03
Nuclei_Texture_Entropy_Mito_10_00
Nuclei_Texture_Entropy_Mito_10_01
Nuclei_Texture_Entropy_Mito_10_02
Nuclei_Texture_Entropy_Mito_10_03
Nuclei_Texture_Entropy_Mito_20_00
Nuclei_Texture_Entropy_Mito_20_01
Nuclei_Texture_Entropy_Mito_20_02
Nuclei_Texture_Entropy_Mito_20_03
Nuclei_Texture_Entropy_Mito_5_00
Nuclei_Texture_Entropy_Mito_5_01
Nuclei_Texture_Entropy_Mito_5_02
Nuclei_Texture_Entropy_Mito_5_03
Nuclei_Texture_Entropy_RNA_10_00
Nuclei_Texture_Entropy_RNA_10_01
Nuclei_Texture_Entropy_RNA_10_02
Nuclei_Texture_Entropy_RNA_10_03
Nuclei_Texture_Entropy_RNA_20_00
Nuclei_Texture_Entropy_RNA_20_01
Nuclei_Texture_Entropy_RNA_20_02
Nuclei_Texture_Entropy_RNA_20_03
Nuclei_Texture_Entropy_RNA_5_00
Nuclei_Texture_Entropy_RNA_5_01
Nuclei_Texture_Entropy_RNA_5_02
Nuclei_Texture_Entropy_RNA_5_03
Nuclei_Texture_InfoMeas1_AGP_10_00
Nuclei_Texture_InfoMeas1_AGP_10_01
Nuclei_Texture_InfoMeas1_AGP_10_02
Nuclei_Texture_InfoMeas1_AGP_10_03
Nuclei_Texture_InfoMeas1_AGP_20_00
Nuclei_Texture_InfoMeas1_AGP_20_01
Nuclei_Texture_InfoMeas1_AGP_20_02

Nuclei_Texture_InfoMeas1_AGP_20_03
Nuclei_Texture_InfoMeas1_AGP_5_00
Nuclei_Texture_InfoMeas1_AGP_5_01
Nuclei_Texture_InfoMeas1_AGP_5_02
Nuclei_Texture_InfoMeas1_AGP_5_03
Nuclei_Texture_InfoMeas1_Brightfield_10_00
Nuclei_Texture_InfoMeas1_Brightfield_10_01
Nuclei_Texture_InfoMeas1_Brightfield_10_02
Nuclei_Texture_InfoMeas1_Brightfield_10_03
Nuclei_Texture_InfoMeas1_Brightfield_20_00
Nuclei_Texture_InfoMeas1_Brightfield_20_01
Nuclei_Texture_InfoMeas1_Brightfield_20_02
Nuclei_Texture_InfoMeas1_Brightfield_20_03
Nuclei_Texture_InfoMeas1_Brightfield_5_00
Nuclei_Texture_InfoMeas1_Brightfield_5_01
Nuclei_Texture_InfoMeas1_Brightfield_5_02
Nuclei_Texture_InfoMeas1_Brightfield_5_03
Nuclei_Texture_InfoMeas1_DNA_10_00
Nuclei_Texture_InfoMeas1_DNA_10_01
Nuclei_Texture_InfoMeas1_DNA_10_02
Nuclei_Texture_InfoMeas1_DNA_10_03
Nuclei_Texture_InfoMeas1_DNA_20_00
Nuclei_Texture_InfoMeas1_DNA_20_01
Nuclei_Texture_InfoMeas1_DNA_20_02
Nuclei_Texture_InfoMeas1_DNA_20_03
Nuclei_Texture_InfoMeas1_DNA_5_00
Nuclei_Texture_InfoMeas1_DNA_5_01
Nuclei_Texture_InfoMeas1_DNA_5_02
Nuclei_Texture_InfoMeas1_DNA_5_03
Nuclei_Texture_InfoMeas1_ER_10_00
Nuclei_Texture_InfoMeas1_ER_10_01
Nuclei_Texture_InfoMeas1_ER_10_02
Nuclei_Texture_InfoMeas1_ER_10_03
Nuclei_Texture_InfoMeas1_ER_20_00
Nuclei_Texture_InfoMeas1_ER_20_01
Nuclei_Texture_InfoMeas1_ER_20_02
Nuclei_Texture_InfoMeas1_ER_20_03
Nuclei_Texture_InfoMeas1_ER_5_00
Nuclei_Texture_InfoMeas1_ER_5_01

Nuclei_Texture_InfoMeas1_ER_5_02
Nuclei_Texture_InfoMeas1_ER_5_03
Nuclei_Texture_InfoMeas1_Mito_10_00
Nuclei_Texture_InfoMeas1_Mito_10_01
Nuclei_Texture_InfoMeas1_Mito_10_02
Nuclei_Texture_InfoMeas1_Mito_10_03
Nuclei_Texture_InfoMeas1_Mito_20_00
Nuclei_Texture_InfoMeas1_Mito_20_01
Nuclei_Texture_InfoMeas1_Mito_20_02
Nuclei_Texture_InfoMeas1_Mito_20_03
Nuclei_Texture_InfoMeas1_Mito_5_00
Nuclei_Texture_InfoMeas1_Mito_5_01
Nuclei_Texture_InfoMeas1_Mito_5_02
Nuclei_Texture_InfoMeas1_Mito_5_03
Nuclei_Texture_InfoMeas1_RNA_10_00
Nuclei_Texture_InfoMeas1_RNA_10_01
Nuclei_Texture_InfoMeas1_RNA_10_02
Nuclei_Texture_InfoMeas1_RNA_10_03
Nuclei_Texture_InfoMeas1_RNA_20_00
Nuclei_Texture_InfoMeas1_RNA_20_01
Nuclei_Texture_InfoMeas1_RNA_20_02
Nuclei_Texture_InfoMeas1_RNA_20_03
Nuclei_Texture_InfoMeas1_RNA_5_00
Nuclei_Texture_InfoMeas1_RNA_5_01
Nuclei_Texture_InfoMeas1_RNA_5_02
Nuclei_Texture_InfoMeas1_RNA_5_03
Nuclei_Texture_InfoMeas2_AGP_10_00
Nuclei_Texture_InfoMeas2_AGP_10_01
Nuclei_Texture_InfoMeas2_AGP_10_02
Nuclei_Texture_InfoMeas2_AGP_10_03
Nuclei_Texture_InfoMeas2_AGP_20_00
Nuclei_Texture_InfoMeas2_AGP_20_01
Nuclei_Texture_InfoMeas2_AGP_20_02
Nuclei_Texture_InfoMeas2_AGP_20_03
Nuclei_Texture_InfoMeas2_AGP_5_00
Nuclei_Texture_InfoMeas2_AGP_5_01
Nuclei_Texture_InfoMeas2_AGP_5_02
Nuclei_Texture_InfoMeas2_AGP_5_03
Nuclei_Texture_InfoMeas2_Brightfield_10_00

Nuclei_Texture_InfoMeas2_Brightfield_10_01
Nuclei_Texture_InfoMeas2_Brightfield_10_02
Nuclei_Texture_InfoMeas2_Brightfield_10_03
Nuclei_Texture_InfoMeas2_Brightfield_20_00
Nuclei_Texture_InfoMeas2_Brightfield_20_01
Nuclei_Texture_InfoMeas2_Brightfield_20_02
Nuclei_Texture_InfoMeas2_Brightfield_20_03
Nuclei_Texture_InfoMeas2_Brightfield_5_00
Nuclei_Texture_InfoMeas2_Brightfield_5_01
Nuclei_Texture_InfoMeas2_Brightfield_5_02
Nuclei_Texture_InfoMeas2_Brightfield_5_03
Nuclei_Texture_InfoMeas2_DNA_10_00
Nuclei_Texture_InfoMeas2_DNA_10_01
Nuclei_Texture_InfoMeas2_DNA_10_02
Nuclei_Texture_InfoMeas2_DNA_10_03
Nuclei_Texture_InfoMeas2_DNA_20_00
Nuclei_Texture_InfoMeas2_DNA_20_01
Nuclei_Texture_InfoMeas2_DNA_20_02
Nuclei_Texture_InfoMeas2_DNA_20_03
Nuclei_Texture_InfoMeas2_DNA_5_00
Nuclei_Texture_InfoMeas2_DNA_5_01
Nuclei_Texture_InfoMeas2_DNA_5_02
Nuclei_Texture_InfoMeas2_DNA_5_03
Nuclei_Texture_InfoMeas2_ER_10_00
Nuclei_Texture_InfoMeas2_ER_10_01
Nuclei_Texture_InfoMeas2_ER_10_02
Nuclei_Texture_InfoMeas2_ER_10_03
Nuclei_Texture_InfoMeas2_ER_20_00
Nuclei_Texture_InfoMeas2_ER_20_01
Nuclei_Texture_InfoMeas2_ER_20_02
Nuclei_Texture_InfoMeas2_ER_20_03
Nuclei_Texture_InfoMeas2_ER_5_00
Nuclei_Texture_InfoMeas2_ER_5_01
Nuclei_Texture_InfoMeas2_ER_5_02
Nuclei_Texture_InfoMeas2_ER_5_03
Nuclei_Texture_InfoMeas2_Mito_10_00
Nuclei_Texture_InfoMeas2_Mito_10_01
Nuclei_Texture_InfoMeas2_Mito_10_02
Nuclei_Texture_InfoMeas2_Mito_10_03

Nuclei_Texture_InfoMeas2_Mito_20_00
Nuclei_Texture_InfoMeas2_Mito_20_01
Nuclei_Texture_InfoMeas2_Mito_20_02
Nuclei_Texture_InfoMeas2_Mito_20_03
Nuclei_Texture_InfoMeas2_Mito_5_00
Nuclei_Texture_InfoMeas2_Mito_5_01
Nuclei_Texture_InfoMeas2_Mito_5_02
Nuclei_Texture_InfoMeas2_Mito_5_03
Nuclei_Texture_InfoMeas2_RNA_10_00
Nuclei_Texture_InfoMeas2_RNA_10_01
Nuclei_Texture_InfoMeas2_RNA_10_02
Nuclei_Texture_InfoMeas2_RNA_10_03
Nuclei_Texture_InfoMeas2_RNA_20_00
Nuclei_Texture_InfoMeas2_RNA_20_01
Nuclei_Texture_InfoMeas2_RNA_20_02
Nuclei_Texture_InfoMeas2_RNA_20_03
Nuclei_Texture_InfoMeas2_RNA_5_00
Nuclei_Texture_InfoMeas2_RNA_5_01
Nuclei_Texture_InfoMeas2_RNA_5_02
Nuclei_Texture_InfoMeas2_RNA_5_03
Nuclei_Texture_InverseDifferenceMoment_AGP_10_00
Nuclei_Texture_InverseDifferenceMoment_AGP_10_01
Nuclei_Texture_InverseDifferenceMoment_AGP_10_02
Nuclei_Texture_InverseDifferenceMoment_AGP_10_03
Nuclei_Texture_InverseDifferenceMoment_AGP_20_00
Nuclei_Texture_InverseDifferenceMoment_AGP_20_01
Nuclei_Texture_InverseDifferenceMoment_AGP_20_02
Nuclei_Texture_InverseDifferenceMoment_AGP_20_03
Nuclei_Texture_InverseDifferenceMoment_AGP_5_00
Nuclei_Texture_InverseDifferenceMoment_AGP_5_01
Nuclei_Texture_InverseDifferenceMoment_AGP_5_02
Nuclei_Texture_InverseDifferenceMoment_AGP_5_03
Nuclei_Texture_InverseDifferenceMoment_Brightfield_10_00
Nuclei_Texture_InverseDifferenceMoment_Brightfield_10_01
Nuclei_Texture_InverseDifferenceMoment_Brightfield_10_02
Nuclei_Texture_InverseDifferenceMoment_Brightfield_10_03
Nuclei_Texture_InverseDifferenceMoment_Brightfield_20_00
Nuclei_Texture_InverseDifferenceMoment_Brightfield_20_01
Nuclei_Texture_InverseDifferenceMoment_Brightfield_20_02

Nuclei_Texture_InverseDifferenceMoment_Brightfield_20_03
Nuclei_Texture_InverseDifferenceMoment_Brightfield_5_00
Nuclei_Texture_InverseDifferenceMoment_Brightfield_5_01
Nuclei_Texture_InverseDifferenceMoment_Brightfield_5_02
Nuclei_Texture_InverseDifferenceMoment_Brightfield_5_03
Nuclei_Texture_InverseDifferenceMoment_DNA_10_00
Nuclei_Texture_InverseDifferenceMoment_DNA_10_01
Nuclei_Texture_InverseDifferenceMoment_DNA_10_02
Nuclei_Texture_InverseDifferenceMoment_DNA_10_03
Nuclei_Texture_InverseDifferenceMoment_DNA_20_00
Nuclei_Texture_InverseDifferenceMoment_DNA_20_01
Nuclei_Texture_InverseDifferenceMoment_DNA_20_02
Nuclei_Texture_InverseDifferenceMoment_DNA_20_03
Nuclei_Texture_InverseDifferenceMoment_DNA_5_00
Nuclei_Texture_InverseDifferenceMoment_DNA_5_01
Nuclei_Texture_InverseDifferenceMoment_DNA_5_02
Nuclei_Texture_InverseDifferenceMoment_DNA_5_03
Nuclei_Texture_InverseDifferenceMoment_ER_10_00
Nuclei_Texture_InverseDifferenceMoment_ER_10_01
Nuclei_Texture_InverseDifferenceMoment_ER_10_02
Nuclei_Texture_InverseDifferenceMoment_ER_10_03
Nuclei_Texture_InverseDifferenceMoment_ER_20_00
Nuclei_Texture_InverseDifferenceMoment_ER_20_01
Nuclei_Texture_InverseDifferenceMoment_ER_20_02
Nuclei_Texture_InverseDifferenceMoment_ER_20_03
Nuclei_Texture_InverseDifferenceMoment_ER_5_00
Nuclei_Texture_InverseDifferenceMoment_ER_5_01
Nuclei_Texture_InverseDifferenceMoment_ER_5_02
Nuclei_Texture_InverseDifferenceMoment_ER_5_03
Nuclei_Texture_InverseDifferenceMoment_Mito_10_00
Nuclei_Texture_InverseDifferenceMoment_Mito_10_01
Nuclei_Texture_InverseDifferenceMoment_Mito_10_02
Nuclei_Texture_InverseDifferenceMoment_Mito_10_03
Nuclei_Texture_InverseDifferenceMoment_Mito_20_00
Nuclei_Texture_InverseDifferenceMoment_Mito_20_01
Nuclei_Texture_InverseDifferenceMoment_Mito_20_02
Nuclei_Texture_InverseDifferenceMoment_Mito_20_03
Nuclei_Texture_InverseDifferenceMoment_Mito_5_00
Nuclei_Texture_InverseDifferenceMoment_Mito_5_01

Nuclei_Texture_InverseDifferenceMoment_Mito_5_02
Nuclei_Texture_InverseDifferenceMoment_Mito_5_03
Nuclei_Texture_InverseDifferenceMoment_RNA_10_00
Nuclei_Texture_InverseDifferenceMoment_RNA_10_01
Nuclei_Texture_InverseDifferenceMoment_RNA_10_02
Nuclei_Texture_InverseDifferenceMoment_RNA_10_03
Nuclei_Texture_InverseDifferenceMoment_RNA_20_00
Nuclei_Texture_InverseDifferenceMoment_RNA_20_01
Nuclei_Texture_InverseDifferenceMoment_RNA_20_02
Nuclei_Texture_InverseDifferenceMoment_RNA_20_03
Nuclei_Texture_InverseDifferenceMoment_RNA_5_00
Nuclei_Texture_InverseDifferenceMoment_RNA_5_01
Nuclei_Texture_InverseDifferenceMoment_RNA_5_02
Nuclei_Texture_InverseDifferenceMoment_RNA_5_03
Nuclei_Texture_SumAverage_AGP_10_00
Nuclei_Texture_SumAverage_AGP_10_01
Nuclei_Texture_SumAverage_AGP_10_02
Nuclei_Texture_SumAverage_AGP_10_03
Nuclei_Texture_SumAverage_AGP_20_00
Nuclei_Texture_SumAverage_AGP_20_01
Nuclei_Texture_SumAverage_AGP_20_02
Nuclei_Texture_SumAverage_AGP_20_03
Nuclei_Texture_SumAverage_AGP_5_00
Nuclei_Texture_SumAverage_AGP_5_01
Nuclei_Texture_SumAverage_AGP_5_02
Nuclei_Texture_SumAverage_AGP_5_03
Nuclei_Texture_SumAverage_Brightfield_10_00
Nuclei_Texture_SumAverage_Brightfield_10_01
Nuclei_Texture_SumAverage_Brightfield_10_02
Nuclei_Texture_SumAverage_Brightfield_10_03
Nuclei_Texture_SumAverage_Brightfield_20_00
Nuclei_Texture_SumAverage_Brightfield_20_01
Nuclei_Texture_SumAverage_Brightfield_20_02
Nuclei_Texture_SumAverage_Brightfield_20_03
Nuclei_Texture_SumAverage_Brightfield_5_00
Nuclei_Texture_SumAverage_Brightfield_5_01
Nuclei_Texture_SumAverage_Brightfield_5_02
Nuclei_Texture_SumAverage_Brightfield_5_03
Nuclei_Texture_SumAverage_DNA_10_00

Nuclei_Texture_SumAverage_DNA_10_01
Nuclei_Texture_SumAverage_DNA_10_02
Nuclei_Texture_SumAverage_DNA_10_03
Nuclei_Texture_SumAverage_DNA_20_00
Nuclei_Texture_SumAverage_DNA_20_01
Nuclei_Texture_SumAverage_DNA_20_02
Nuclei_Texture_SumAverage_DNA_20_03
Nuclei_Texture_SumAverage_DNA_5_00
Nuclei_Texture_SumAverage_DNA_5_01
Nuclei_Texture_SumAverage_DNA_5_02
Nuclei_Texture_SumAverage_DNA_5_03
Nuclei_Texture_SumAverage_ER_10_00
Nuclei_Texture_SumAverage_ER_10_01
Nuclei_Texture_SumAverage_ER_10_02
Nuclei_Texture_SumAverage_ER_10_03
Nuclei_Texture_SumAverage_ER_20_00
Nuclei_Texture_SumAverage_ER_20_01
Nuclei_Texture_SumAverage_ER_20_02
Nuclei_Texture_SumAverage_ER_20_03
Nuclei_Texture_SumAverage_ER_5_00
Nuclei_Texture_SumAverage_ER_5_01
Nuclei_Texture_SumAverage_ER_5_02
Nuclei_Texture_SumAverage_ER_5_03
Nuclei_Texture_SumAverage_Mito_10_00
Nuclei_Texture_SumAverage_Mito_10_01
Nuclei_Texture_SumAverage_Mito_10_02
Nuclei_Texture_SumAverage_Mito_10_03
Nuclei_Texture_SumAverage_Mito_20_00
Nuclei_Texture_SumAverage_Mito_20_01
Nuclei_Texture_SumAverage_Mito_20_02
Nuclei_Texture_SumAverage_Mito_20_03
Nuclei_Texture_SumAverage_Mito_5_00
Nuclei_Texture_SumAverage_Mito_5_01
Nuclei_Texture_SumAverage_Mito_5_02
Nuclei_Texture_SumAverage_Mito_5_03
Nuclei_Texture_SumAverage_RNA_10_00
Nuclei_Texture_SumAverage_RNA_10_01
Nuclei_Texture_SumAverage_RNA_10_02
Nuclei_Texture_SumAverage_RNA_10_03

Nuclei_Texture_SumAverage_RNA_20_00
Nuclei_Texture_SumAverage_RNA_20_01
Nuclei_Texture_SumAverage_RNA_20_02
Nuclei_Texture_SumAverage_RNA_20_03
Nuclei_Texture_SumAverage_RNA_5_00
Nuclei_Texture_SumAverage_RNA_5_01
Nuclei_Texture_SumAverage_RNA_5_02
Nuclei_Texture_SumAverage_RNA_5_03
Nuclei_Texture_SumEntropy_AGP_10_00
Nuclei_Texture_SumEntropy_AGP_10_01
Nuclei_Texture_SumEntropy_AGP_10_02
Nuclei_Texture_SumEntropy_AGP_10_03
Nuclei_Texture_SumEntropy_AGP_20_00
Nuclei_Texture_SumEntropy_AGP_20_01
Nuclei_Texture_SumEntropy_AGP_20_02
Nuclei_Texture_SumEntropy_AGP_20_03
Nuclei_Texture_SumEntropy_AGP_5_00
Nuclei_Texture_SumEntropy_AGP_5_01
Nuclei_Texture_SumEntropy_AGP_5_02
Nuclei_Texture_SumEntropy_AGP_5_03
Nuclei_Texture_SumEntropy_Brightfield_10_00
Nuclei_Texture_SumEntropy_Brightfield_10_01
Nuclei_Texture_SumEntropy_Brightfield_10_02
Nuclei_Texture_SumEntropy_Brightfield_10_03
Nuclei_Texture_SumEntropy_Brightfield_20_00
Nuclei_Texture_SumEntropy_Brightfield_20_01
Nuclei_Texture_SumEntropy_Brightfield_20_02
Nuclei_Texture_SumEntropy_Brightfield_20_03
Nuclei_Texture_SumEntropy_Brightfield_5_00
Nuclei_Texture_SumEntropy_Brightfield_5_01
Nuclei_Texture_SumEntropy_Brightfield_5_02
Nuclei_Texture_SumEntropy_Brightfield_5_03
Nuclei_Texture_SumEntropy_DNA_10_00
Nuclei_Texture_SumEntropy_DNA_10_01
Nuclei_Texture_SumEntropy_DNA_10_02
Nuclei_Texture_SumEntropy_DNA_10_03
Nuclei_Texture_SumEntropy_DNA_20_00
Nuclei_Texture_SumEntropy_DNA_20_01
Nuclei_Texture_SumEntropy_DNA_20_02

Nuclei_Texture_SumEntropy_DNA_20_03
Nuclei_Texture_SumEntropy_DNA_5_00
Nuclei_Texture_SumEntropy_DNA_5_01
Nuclei_Texture_SumEntropy_DNA_5_02
Nuclei_Texture_SumEntropy_DNA_5_03
Nuclei_Texture_SumEntropy_ER_10_00
Nuclei_Texture_SumEntropy_ER_10_01
Nuclei_Texture_SumEntropy_ER_10_02
Nuclei_Texture_SumEntropy_ER_10_03
Nuclei_Texture_SumEntropy_ER_20_00
Nuclei_Texture_SumEntropy_ER_20_01
Nuclei_Texture_SumEntropy_ER_20_02
Nuclei_Texture_SumEntropy_ER_20_03
Nuclei_Texture_SumEntropy_ER_5_00
Nuclei_Texture_SumEntropy_ER_5_01
Nuclei_Texture_SumEntropy_ER_5_02
Nuclei_Texture_SumEntropy_ER_5_03
Nuclei_Texture_SumEntropy_Mito_10_00
Nuclei_Texture_SumEntropy_Mito_10_01
Nuclei_Texture_SumEntropy_Mito_10_02
Nuclei_Texture_SumEntropy_Mito_10_03
Nuclei_Texture_SumEntropy_Mito_20_00
Nuclei_Texture_SumEntropy_Mito_20_01
Nuclei_Texture_SumEntropy_Mito_20_02
Nuclei_Texture_SumEntropy_Mito_20_03
Nuclei_Texture_SumEntropy_Mito_5_00
Nuclei_Texture_SumEntropy_Mito_5_01
Nuclei_Texture_SumEntropy_Mito_5_02
Nuclei_Texture_SumEntropy_Mito_5_03
Nuclei_Texture_SumEntropy_RNA_10_00
Nuclei_Texture_SumEntropy_RNA_10_01
Nuclei_Texture_SumEntropy_RNA_10_02
Nuclei_Texture_SumEntropy_RNA_10_03
Nuclei_Texture_SumEntropy_RNA_20_00
Nuclei_Texture_SumEntropy_RNA_20_01
Nuclei_Texture_SumEntropy_RNA_20_02
Nuclei_Texture_SumEntropy_RNA_20_03
Nuclei_Texture_SumEntropy_RNA_5_00
Nuclei_Texture_SumEntropy_RNA_5_01

Nuclei_Texture_SumEntropy_RNA_5_02
Nuclei_Texture_SumEntropy_RNA_5_03
Nuclei_Texture_SumVariance_AGP_10_00
Nuclei_Texture_SumVariance_AGP_10_01
Nuclei_Texture_SumVariance_AGP_10_02
Nuclei_Texture_SumVariance_AGP_10_03
Nuclei_Texture_SumVariance_AGP_20_00
Nuclei_Texture_SumVariance_AGP_20_01
Nuclei_Texture_SumVariance_AGP_20_02
Nuclei_Texture_SumVariance_AGP_20_03
Nuclei_Texture_SumVariance_AGP_5_00
Nuclei_Texture_SumVariance_AGP_5_01
Nuclei_Texture_SumVariance_AGP_5_02
Nuclei_Texture_SumVariance_AGP_5_03
Nuclei_Texture_SumVariance_Brightfield_10_00
Nuclei_Texture_SumVariance_Brightfield_10_01
Nuclei_Texture_SumVariance_Brightfield_10_02
Nuclei_Texture_SumVariance_Brightfield_10_03
Nuclei_Texture_SumVariance_Brightfield_20_00
Nuclei_Texture_SumVariance_Brightfield_20_01
Nuclei_Texture_SumVariance_Brightfield_20_02
Nuclei_Texture_SumVariance_Brightfield_20_03
Nuclei_Texture_SumVariance_Brightfield_5_00
Nuclei_Texture_SumVariance_Brightfield_5_01
Nuclei_Texture_SumVariance_Brightfield_5_02
Nuclei_Texture_SumVariance_Brightfield_5_03
Nuclei_Texture_SumVariance_DNA_10_00
Nuclei_Texture_SumVariance_DNA_10_01
Nuclei_Texture_SumVariance_DNA_10_02
Nuclei_Texture_SumVariance_DNA_10_03
Nuclei_Texture_SumVariance_DNA_20_00
Nuclei_Texture_SumVariance_DNA_20_01
Nuclei_Texture_SumVariance_DNA_20_02
Nuclei_Texture_SumVariance_DNA_20_03
Nuclei_Texture_SumVariance_DNA_5_00
Nuclei_Texture_SumVariance_DNA_5_01
Nuclei_Texture_SumVariance_DNA_5_02
Nuclei_Texture_SumVariance_DNA_5_03
Nuclei_Texture_SumVariance_ER_10_00

Nuclei_Texture_SumVariance_ER_10_01
Nuclei_Texture_SumVariance_ER_10_02
Nuclei_Texture_SumVariance_ER_10_03
Nuclei_Texture_SumVariance_ER_20_00
Nuclei_Texture_SumVariance_ER_20_01
Nuclei_Texture_SumVariance_ER_20_02
Nuclei_Texture_SumVariance_ER_20_03
Nuclei_Texture_SumVariance_ER_5_00
Nuclei_Texture_SumVariance_ER_5_01
Nuclei_Texture_SumVariance_ER_5_02
Nuclei_Texture_SumVariance_ER_5_03
Nuclei_Texture_SumVariance_Mito_10_00
Nuclei_Texture_SumVariance_Mito_10_01
Nuclei_Texture_SumVariance_Mito_10_02
Nuclei_Texture_SumVariance_Mito_10_03
Nuclei_Texture_SumVariance_Mito_20_00
Nuclei_Texture_SumVariance_Mito_20_01
Nuclei_Texture_SumVariance_Mito_20_02
Nuclei_Texture_SumVariance_Mito_20_03
Nuclei_Texture_SumVariance_Mito_5_00
Nuclei_Texture_SumVariance_Mito_5_01
Nuclei_Texture_SumVariance_Mito_5_02
Nuclei_Texture_SumVariance_Mito_5_03
Nuclei_Texture_SumVariance_RNA_10_00
Nuclei_Texture_SumVariance_RNA_10_01
Nuclei_Texture_SumVariance_RNA_10_02
Nuclei_Texture_SumVariance_RNA_10_03
Nuclei_Texture_SumVariance_RNA_20_00
Nuclei_Texture_SumVariance_RNA_20_01
Nuclei_Texture_SumVariance_RNA_20_02
Nuclei_Texture_SumVariance_RNA_20_03
Nuclei_Texture_SumVariance_RNA_5_00
Nuclei_Texture_SumVariance_RNA_5_01
Nuclei_Texture_SumVariance_RNA_5_02
Nuclei_Texture_SumVariance_RNA_5_03
Nuclei_Texture_Variance_AGP_10_00
Nuclei_Texture_Variance_AGP_10_01
Nuclei_Texture_Variance_AGP_10_02
Nuclei_Texture_Variance_AGP_10_03

Nuclei_Texture_Variance_AGP_20_00
Nuclei_Texture_Variance_AGP_20_01
Nuclei_Texture_Variance_AGP_20_02
Nuclei_Texture_Variance_AGP_20_03
Nuclei_Texture_Variance_AGP_5_00
Nuclei_Texture_Variance_AGP_5_01
Nuclei_Texture_Variance_AGP_5_02
Nuclei_Texture_Variance_AGP_5_03
Nuclei_Texture_Variance_Brightfield_10_00
Nuclei_Texture_Variance_Brightfield_10_01
Nuclei_Texture_Variance_Brightfield_10_02
Nuclei_Texture_Variance_Brightfield_10_03
Nuclei_Texture_Variance_Brightfield_20_00
Nuclei_Texture_Variance_Brightfield_20_01
Nuclei_Texture_Variance_Brightfield_20_02
Nuclei_Texture_Variance_Brightfield_20_03
Nuclei_Texture_Variance_Brightfield_5_00
Nuclei_Texture_Variance_Brightfield_5_01
Nuclei_Texture_Variance_Brightfield_5_02
Nuclei_Texture_Variance_Brightfield_5_03
Nuclei_Texture_Variance_DNA_10_00
Nuclei_Texture_Variance_DNA_10_01
Nuclei_Texture_Variance_DNA_10_02
Nuclei_Texture_Variance_DNA_10_03
Nuclei_Texture_Variance_DNA_20_00
Nuclei_Texture_Variance_DNA_20_01
Nuclei_Texture_Variance_DNA_20_02
Nuclei_Texture_Variance_DNA_20_03
Nuclei_Texture_Variance_DNA_5_00
Nuclei_Texture_Variance_DNA_5_01
Nuclei_Texture_Variance_DNA_5_02
Nuclei_Texture_Variance_DNA_5_03
Nuclei_Texture_Variance_ER_10_00
Nuclei_Texture_Variance_ER_10_01
Nuclei_Texture_Variance_ER_10_02
Nuclei_Texture_Variance_ER_10_03
Nuclei_Texture_Variance_ER_20_00
Nuclei_Texture_Variance_ER_20_01
Nuclei_Texture_Variance_ER_20_02

Nuclei_Texture_Variance_ER_20_03
Nuclei_Texture_Variance_ER_5_00
Nuclei_Texture_Variance_ER_5_01
Nuclei_Texture_Variance_ER_5_02
Nuclei_Texture_Variance_ER_5_03
Nuclei_Texture_Variance_Mito_10_00
Nuclei_Texture_Variance_Mito_10_01
Nuclei_Texture_Variance_Mito_10_02
Nuclei_Texture_Variance_Mito_10_03
Nuclei_Texture_Variance_Mito_20_00
Nuclei_Texture_Variance_Mito_20_01
Nuclei_Texture_Variance_Mito_20_02
Nuclei_Texture_Variance_Mito_20_03
Nuclei_Texture_Variance_Mito_5_00
Nuclei_Texture_Variance_Mito_5_01
Nuclei_Texture_Variance_Mito_5_02
Nuclei_Texture_Variance_Mito_5_03
Nuclei_Texture_Variance_RNA_10_00
Nuclei_Texture_Variance_RNA_10_01
Nuclei_Texture_Variance_RNA_10_02
Nuclei_Texture_Variance_RNA_10_03
Nuclei_Texture_Variance_RNA_20_00
Nuclei_Texture_Variance_RNA_20_01
Nuclei_Texture_Variance_RNA_20_02
Nuclei_Texture_Variance_RNA_20_03
Nuclei_Texture_Variance_RNA_5_00
Nuclei_Texture_Variance_RNA_5_01
Nuclei_Texture_Variance_RNA_5_02
Nuclei_Texture_Variance_RNA_5_03
Cytoplasm_AreaShape_Area
Cytoplasm_AreaShape_Compactness
Cytoplasm_AreaShape_Eccentricity
Cytoplasm_AreaShape_Extent
Cytoplasm_AreaShape_FormFactor
Cytoplasm_AreaShape_MajorAxisLength
Cytoplasm_AreaShape_MaxFeretDiameter
Cytoplasm_AreaShape_MaximumRadius
Cytoplasm_AreaShape_MeanRadius
Cytoplasm_AreaShape_MedianRadius

Cytoplasm_AreaShape_MinFeretDiameter
Cytoplasm_AreaShape_MinorAxisLength
Cytoplasm_AreaShape_Perimeter
Cytoplasm_AreaShape_Solidity
Cytoplasm_AreaShape_Zernike_0_0
Cytoplasm_AreaShape_Zernike_1_1
Cytoplasm_AreaShape_Zernike_2_0
Cytoplasm_AreaShape_Zernike_2_2
Cytoplasm_AreaShape_Zernike_3_1
Cytoplasm_AreaShape_Zernike_3_3
Cytoplasm_AreaShape_Zernike_4_0
Cytoplasm_AreaShape_Zernike_4_2
Cytoplasm_AreaShape_Zernike_4_4
Cytoplasm_AreaShape_Zernike_5_1
Cytoplasm_AreaShape_Zernike_5_3
Cytoplasm_AreaShape_Zernike_5_5
Cytoplasm_AreaShape_Zernike_6_0
Cytoplasm_AreaShape_Zernike_6_2
Cytoplasm_AreaShape_Zernike_6_4
Cytoplasm_AreaShape_Zernike_6_6
Cytoplasm_AreaShape_Zernike_7_1
Cytoplasm_AreaShape_Zernike_7_3
Cytoplasm_AreaShape_Zernike_7_5
Cytoplasm_AreaShape_Zernike_7_7
Cytoplasm_AreaShape_Zernike_8_0
Cytoplasm_AreaShape_Zernike_8_2
Cytoplasm_AreaShape_Zernike_8_4
Cytoplasm_AreaShape_Zernike_8_6
Cytoplasm_AreaShape_Zernike_8_8
Cytoplasm_AreaShape_Zernike_9_1
Cytoplasm_AreaShape_Zernike_9_3
Cytoplasm_AreaShape_Zernike_9_5
Cytoplasm_AreaShape_Zernike_9_7
Cytoplasm_AreaShape_Zernike_9_9
Cytoplasm_Granularity_10_AGP
Cytoplasm_Granularity_10_Brightfield
Cytoplasm_Granularity_10_ER
Cytoplasm_Granularity_10_Mito
Cytoplasm_Granularity_10_RNA

Cytoplasm_Granularity_11_AGP
Cytoplasm_Granularity_11_Brightfield
Cytoplasm_Granularity_11_ER
Cytoplasm_Granularity_11_Mito
Cytoplasm_Granularity_11_RNA
Cytoplasm_Granularity_12_AGP
Cytoplasm_Granularity_12_Brightfield
Cytoplasm_Granularity_12_ER
Cytoplasm_Granularity_12_Mito
Cytoplasm_Granularity_12_RNA
Cytoplasm_Granularity_13_AGP
Cytoplasm_Granularity_13_Brightfield
Cytoplasm_Granularity_13_ER
Cytoplasm_Granularity_13_Mito
Cytoplasm_Granularity_13_RNA
Cytoplasm_Granularity_1_AGP
Cytoplasm_Granularity_1_Brightfield
Cytoplasm_Granularity_1_ER
Cytoplasm_Granularity_1_Mito
Cytoplasm_Granularity_1_RNA
Cytoplasm_Granularity_2_AGP
Cytoplasm_Granularity_2_Brightfield
Cytoplasm_Granularity_2_ER
Cytoplasm_Granularity_2_Mito
Cytoplasm_Granularity_2_RNA
Cytoplasm_Granularity_3_AGP
Cytoplasm_Granularity_3_Brightfield
Cytoplasm_Granularity_3_ER
Cytoplasm_Granularity_3_Mito
Cytoplasm_Granularity_3_RNA
Cytoplasm_Granularity_4_AGP
Cytoplasm_Granularity_4_Brightfield
Cytoplasm_Granularity_4_ER
Cytoplasm_Granularity_4_Mito
Cytoplasm_Granularity_4_RNA
Cytoplasm_Granularity_5_AGP
Cytoplasm_Granularity_5_Brightfield
Cytoplasm_Granularity_5_ER
Cytoplasm_Granularity_5_Mito

Cytoplasm_Granularity_5_RNA
Cytoplasm_Granularity_6_AGP
Cytoplasm_Granularity_6_Brightfield
Cytoplasm_Granularity_6_ER
Cytoplasm_Granularity_6_Mito
Cytoplasm_Granularity_6_RNA
Cytoplasm_Granularity_7_AGP
Cytoplasm_Granularity_7_Brightfield
Cytoplasm_Granularity_7_ER
Cytoplasm_Granularity_7_Mito
Cytoplasm_Granularity_7_RNA
Cytoplasm_Granularity_8_AGP
Cytoplasm_Granularity_8_Brightfield
Cytoplasm_Granularity_8_ER
Cytoplasm_Granularity_8_Mito
Cytoplasm_Granularity_8_RNA
Cytoplasm_Granularity_9_AGP
Cytoplasm_Granularity_9_Brightfield
Cytoplasm_Granularity_9_ER
Cytoplasm_Granularity_9_Mito
Cytoplasm_Granularity_9_RNA
Cytoplasm_Intensity_IntegratedIntensityEdge_AGP
Cytoplasm_Intensity_IntegratedIntensityEdge_Brightfield
Cytoplasm_Intensity_IntegratedIntensityEdge_DNA
Cytoplasm_Intensity_IntegratedIntensityEdge_ER
Cytoplasm_Intensity_IntegratedIntensityEdge_Mito
Cytoplasm_Intensity_IntegratedIntensityEdge_RNA
Cytoplasm_Intensity_IntegratedIntensity_AGP
Cytoplasm_Intensity_IntegratedIntensity_Brightfield
Cytoplasm_Intensity_IntegratedIntensity_DNA
Cytoplasm_Intensity_IntegratedIntensity_ER
Cytoplasm_Intensity_IntegratedIntensity_Mito
Cytoplasm_Intensity_IntegratedIntensity_RNA
Cytoplasm_Intensity_LowerQuartileIntensity_AGP
Cytoplasm_Intensity_LowerQuartileIntensity_Brightfield
Cytoplasm_Intensity_LowerQuartileIntensity_DNA
Cytoplasm_Intensity_LowerQuartileIntensity_ER
Cytoplasm_Intensity_LowerQuartileIntensity_Mito
Cytoplasm_Intensity_LowerQuartileIntensity_RNA

Cytoplasm_Intensity_MADIntensity_AGP
Cytoplasm_Intensity_MADIntensity_Brightfield
Cytoplasm_Intensity_MADIntensity_DNA
Cytoplasm_Intensity_MADIntensity_ER
Cytoplasm_Intensity_MADIntensity_Mito
Cytoplasm_Intensity_MADIntensity_RNA
Cytoplasm_Intensity_MassDisplacement_AGP
Cytoplasm_Intensity_MassDisplacement_Brightfield
Cytoplasm_Intensity_MassDisplacement_DNA
Cytoplasm_Intensity_MassDisplacement_ER
Cytoplasm_Intensity_MassDisplacement_Mito
Cytoplasm_Intensity_MassDisplacement_RNA
Cytoplasm_Intensity_MaxIntensityEdge_AGP
Cytoplasm_Intensity_MaxIntensityEdge_Brightfield
Cytoplasm_Intensity_MaxIntensityEdge_DNA
Cytoplasm_Intensity_MaxIntensityEdge_ER
Cytoplasm_Intensity_MaxIntensityEdge_Mito
Cytoplasm_Intensity_MaxIntensityEdge_RNA
Cytoplasm_Intensity_MaxIntensity_AGP
Cytoplasm_Intensity_MaxIntensity_Brightfield
Cytoplasm_Intensity_MaxIntensity_DNA
Cytoplasm_Intensity_MaxIntensity_ER
Cytoplasm_Intensity_MaxIntensity_Mito
Cytoplasm_Intensity_MaxIntensity_RNA
Cytoplasm_Intensity_MeanIntensityEdge_AGP
Cytoplasm_Intensity_MeanIntensityEdge_Brightfield
Cytoplasm_Intensity_MeanIntensityEdge_DNA
Cytoplasm_Intensity_MeanIntensityEdge_ER
Cytoplasm_Intensity_MeanIntensityEdge_Mito
Cytoplasm_Intensity_MeanIntensityEdge_RNA
Cytoplasm_Intensity_MeanIntensity_AGP
Cytoplasm_Intensity_MeanIntensity_Brightfield
Cytoplasm_Intensity_MeanIntensity_DNA
Cytoplasm_Intensity_MeanIntensity_ER
Cytoplasm_Intensity_MeanIntensity_Mito
Cytoplasm_Intensity_MeanIntensity_RNA
Cytoplasm_Intensity_MedianIntensity_AGP
Cytoplasm_Intensity_MedianIntensity_Brightfield
Cytoplasm_Intensity_MedianIntensity_DNA

Cytoplasm_Intensity_MedianIntensity_ER
Cytoplasm_Intensity_MedianIntensity_Mito
Cytoplasm_Intensity_MedianIntensity_RNA
Cytoplasm_Intensity_MinIntensityEdge_AGP
Cytoplasm_Intensity_MinIntensityEdge_Brightfield
Cytoplasm_Intensity_MinIntensityEdge_DNA
Cytoplasm_Intensity_MinIntensityEdge_ER
Cytoplasm_Intensity_MinIntensityEdge_Mito
Cytoplasm_Intensity_MinIntensityEdge_RNA
Cytoplasm_Intensity_MinIntensity_AGP
Cytoplasm_Intensity_MinIntensity_Brightfield
Cytoplasm_Intensity_MinIntensity_DNA
Cytoplasm_Intensity_MinIntensity_ER
Cytoplasm_Intensity_MinIntensity_Mito
Cytoplasm_Intensity_MinIntensity_RNA
Cytoplasm_Intensity_StdIntensityEdge_AGP
Cytoplasm_Intensity_StdIntensityEdge_Brightfield
Cytoplasm_Intensity_StdIntensityEdge_DNA
Cytoplasm_Intensity_StdIntensityEdge_ER
Cytoplasm_Intensity_StdIntensityEdge_Mito
Cytoplasm_Intensity_StdIntensityEdge_RNA
Cytoplasm_Intensity_StdIntensity_AGP
Cytoplasm_Intensity_StdIntensity_Brightfield
Cytoplasm_Intensity_StdIntensity_DNA
Cytoplasm_Intensity_StdIntensity_ER
Cytoplasm_Intensity_StdIntensity_Mito
Cytoplasm_Intensity_StdIntensity_RNA
Cytoplasm_Intensity_UpperQuartileIntensity_AGP
Cytoplasm_Intensity_UpperQuartileIntensity_Brightfield
Cytoplasm_Intensity_UpperQuartileIntensity_DNA
Cytoplasm_Intensity_UpperQuartileIntensity_ER
Cytoplasm_Intensity_UpperQuartileIntensity_Mito
Cytoplasm_Intensity_UpperQuartileIntensity_RNA
Cytoplasm_RadialDistribution_FracAtD_AGP_1of4
Cytoplasm_RadialDistribution_FracAtD_AGP_2of4
Cytoplasm_RadialDistribution_FracAtD_AGP_3of4
Cytoplasm_RadialDistribution_FracAtD_AGP_4of4
Cytoplasm_RadialDistribution_FracAtD_Brightfield_1of4
Cytoplasm_RadialDistribution_FracAtD_Brightfield_2of4

Cytoplasm_RadialDistribution_FracAtD_Brightfield_3of4
Cytoplasm_RadialDistribution_FracAtD_Brightfield_4of4
Cytoplasm_RadialDistribution_FracAtD_DNA_1of4
Cytoplasm_RadialDistribution_FracAtD_DNA_2of4
Cytoplasm_RadialDistribution_FracAtD_DNA_3of4
Cytoplasm_RadialDistribution_FracAtD_DNA_4of4
Cytoplasm_RadialDistribution_FracAtD_ER_1of4
Cytoplasm_RadialDistribution_FracAtD_ER_2of4
Cytoplasm_RadialDistribution_FracAtD_ER_3of4
Cytoplasm_RadialDistribution_FracAtD_ER_4of4
Cytoplasm_RadialDistribution_FracAtD_Mito_1of4
Cytoplasm_RadialDistribution_FracAtD_Mito_2of4
Cytoplasm_RadialDistribution_FracAtD_Mito_3of4
Cytoplasm_RadialDistribution_FracAtD_Mito_4of4
Cytoplasm_RadialDistribution_FracAtD_RNA_1of4
Cytoplasm_RadialDistribution_FracAtD_RNA_2of4
Cytoplasm_RadialDistribution_FracAtD_RNA_3of4
Cytoplasm_RadialDistribution_FracAtD_RNA_4of4
Cytoplasm_RadialDistribution_MeanFrac_AGP_1of4
Cytoplasm_RadialDistribution_MeanFrac_AGP_2of4
Cytoplasm_RadialDistribution_MeanFrac_AGP_3of4
Cytoplasm_RadialDistribution_MeanFrac_AGP_4of4
Cytoplasm_RadialDistribution_MeanFrac_Brightfield_1of4
Cytoplasm_RadialDistribution_MeanFrac_Brightfield_2of4
Cytoplasm_RadialDistribution_MeanFrac_Brightfield_3of4
Cytoplasm_RadialDistribution_MeanFrac_Brightfield_4of4
Cytoplasm_RadialDistribution_MeanFrac_DNA_1of4
Cytoplasm_RadialDistribution_MeanFrac_DNA_2of4
Cytoplasm_RadialDistribution_MeanFrac_DNA_3of4
Cytoplasm_RadialDistribution_MeanFrac_DNA_4of4
Cytoplasm_RadialDistribution_MeanFrac_ER_1of4
Cytoplasm_RadialDistribution_MeanFrac_ER_2of4
Cytoplasm_RadialDistribution_MeanFrac_ER_3of4
Cytoplasm_RadialDistribution_MeanFrac_ER_4of4
Cytoplasm_RadialDistribution_MeanFrac_Mito_1of4
Cytoplasm_RadialDistribution_MeanFrac_Mito_2of4
Cytoplasm_RadialDistribution_MeanFrac_Mito_3of4
Cytoplasm_RadialDistribution_MeanFrac_Mito_4of4
Cytoplasm_RadialDistribution_MeanFrac_RNA_1of4

Cytoplasm_RadialDistribution_MeanFrac_RNA_2of4
Cytoplasm_RadialDistribution_MeanFrac_RNA_3of4
Cytoplasm_RadialDistribution_MeanFrac_RNA_4of4
Cytoplasm_RadialDistribution_RadialCV_AGP_1of4
Cytoplasm_RadialDistribution_RadialCV_AGP_2of4
Cytoplasm_RadialDistribution_RadialCV_AGP_3of4
Cytoplasm_RadialDistribution_RadialCV_AGP_4of4
Cytoplasm_RadialDistribution_RadialCV_Brightfield_1of4
Cytoplasm_RadialDistribution_RadialCV_Brightfield_2of4
Cytoplasm_RadialDistribution_RadialCV_Brightfield_3of4
Cytoplasm_RadialDistribution_RadialCV_Brightfield_4of4
Cytoplasm_RadialDistribution_RadialCV_DNA_1of4
Cytoplasm_RadialDistribution_RadialCV_DNA_2of4
Cytoplasm_RadialDistribution_RadialCV_DNA_3of4
Cytoplasm_RadialDistribution_RadialCV_DNA_4of4
Cytoplasm_RadialDistribution_RadialCV_ER_1of4
Cytoplasm_RadialDistribution_RadialCV_ER_2of4
Cytoplasm_RadialDistribution_RadialCV_ER_3of4
Cytoplasm_RadialDistribution_RadialCV_ER_4of4
Cytoplasm_RadialDistribution_RadialCV_Mito_1of4
Cytoplasm_RadialDistribution_RadialCV_Mito_2of4
Cytoplasm_RadialDistribution_RadialCV_Mito_3of4
Cytoplasm_RadialDistribution_RadialCV_Mito_4of4
Cytoplasm_RadialDistribution_RadialCV_RNA_1of4
Cytoplasm_RadialDistribution_RadialCV_RNA_2of4
Cytoplasm_RadialDistribution_RadialCV_RNA_3of4
Cytoplasm_RadialDistribution_RadialCV_RNA_4of4
Cytoplasm_Texture_AngularSecondMoment_AGP_10_00
Cytoplasm_Texture_AngularSecondMoment_AGP_10_01
Cytoplasm_Texture_AngularSecondMoment_AGP_10_02
Cytoplasm_Texture_AngularSecondMoment_AGP_10_03
Cytoplasm_Texture_AngularSecondMoment_AGP_20_00
Cytoplasm_Texture_AngularSecondMoment_AGP_20_01
Cytoplasm_Texture_AngularSecondMoment_AGP_20_02
Cytoplasm_Texture_AngularSecondMoment_AGP_20_03
Cytoplasm_Texture_AngularSecondMoment_AGP_5_00
Cytoplasm_Texture_AngularSecondMoment_AGP_5_01
Cytoplasm_Texture_AngularSecondMoment_AGP_5_02
Cytoplasm_Texture_AngularSecondMoment_AGP_5_03

Cytoplasm_Texture_AngularSecondMoment_Brightfield_10_00
Cytoplasm_Texture_AngularSecondMoment_Brightfield_10_01
Cytoplasm_Texture_AngularSecondMoment_Brightfield_10_02
Cytoplasm_Texture_AngularSecondMoment_Brightfield_10_03
Cytoplasm_Texture_AngularSecondMoment_Brightfield_20_00
Cytoplasm_Texture_AngularSecondMoment_Brightfield_20_01
Cytoplasm_Texture_AngularSecondMoment_Brightfield_20_02
Cytoplasm_Texture_AngularSecondMoment_Brightfield_20_03
Cytoplasm_Texture_AngularSecondMoment_Brightfield_5_00
Cytoplasm_Texture_AngularSecondMoment_Brightfield_5_01
Cytoplasm_Texture_AngularSecondMoment_Brightfield_5_02
Cytoplasm_Texture_AngularSecondMoment_Brightfield_5_03
Cytoplasm_Texture_AngularSecondMoment_DNA_10_00
Cytoplasm_Texture_AngularSecondMoment_DNA_10_01
Cytoplasm_Texture_AngularSecondMoment_DNA_10_02
Cytoplasm_Texture_AngularSecondMoment_DNA_10_03
Cytoplasm_Texture_AngularSecondMoment_DNA_20_00
Cytoplasm_Texture_AngularSecondMoment_DNA_20_01
Cytoplasm_Texture_AngularSecondMoment_DNA_20_02
Cytoplasm_Texture_AngularSecondMoment_DNA_20_03
Cytoplasm_Texture_AngularSecondMoment_DNA_5_00
Cytoplasm_Texture_AngularSecondMoment_DNA_5_01
Cytoplasm_Texture_AngularSecondMoment_DNA_5_02
Cytoplasm_Texture_AngularSecondMoment_DNA_5_03
Cytoplasm_Texture_AngularSecondMoment_ER_10_00
Cytoplasm_Texture_AngularSecondMoment_ER_10_01
Cytoplasm_Texture_AngularSecondMoment_ER_10_02
Cytoplasm_Texture_AngularSecondMoment_ER_10_03
Cytoplasm_Texture_AngularSecondMoment_ER_20_00
Cytoplasm_Texture_AngularSecondMoment_ER_20_01
Cytoplasm_Texture_AngularSecondMoment_ER_20_02
Cytoplasm_Texture_AngularSecondMoment_ER_20_03
Cytoplasm_Texture_AngularSecondMoment_ER_5_00
Cytoplasm_Texture_AngularSecondMoment_ER_5_01
Cytoplasm_Texture_AngularSecondMoment_ER_5_02
Cytoplasm_Texture_AngularSecondMoment_ER_5_03
Cytoplasm_Texture_AngularSecondMoment_Mito_10_00
Cytoplasm_Texture_AngularSecondMoment_Mito_10_01
Cytoplasm_Texture_AngularSecondMoment_Mito_10_02

Cytoplasm_Texture_AngularSecondMoment_Mito_10_03
Cytoplasm_Texture_AngularSecondMoment_Mito_20_00
Cytoplasm_Texture_AngularSecondMoment_Mito_20_01
Cytoplasm_Texture_AngularSecondMoment_Mito_20_02
Cytoplasm_Texture_AngularSecondMoment_Mito_20_03
Cytoplasm_Texture_AngularSecondMoment_Mito_5_00
Cytoplasm_Texture_AngularSecondMoment_Mito_5_01
Cytoplasm_Texture_AngularSecondMoment_Mito_5_02
Cytoplasm_Texture_AngularSecondMoment_Mito_5_03
Cytoplasm_Texture_AngularSecondMoment_RNA_10_00
Cytoplasm_Texture_AngularSecondMoment_RNA_10_01
Cytoplasm_Texture_AngularSecondMoment_RNA_10_02
Cytoplasm_Texture_AngularSecondMoment_RNA_10_03
Cytoplasm_Texture_AngularSecondMoment_RNA_20_00
Cytoplasm_Texture_AngularSecondMoment_RNA_20_01
Cytoplasm_Texture_AngularSecondMoment_RNA_20_02
Cytoplasm_Texture_AngularSecondMoment_RNA_20_03
Cytoplasm_Texture_AngularSecondMoment_RNA_5_00
Cytoplasm_Texture_AngularSecondMoment_RNA_5_01
Cytoplasm_Texture_AngularSecondMoment_RNA_5_02
Cytoplasm_Texture_AngularSecondMoment_RNA_5_03
Cytoplasm_Texture_Contrast_AGP_10_00
Cytoplasm_Texture_Contrast_AGP_10_01
Cytoplasm_Texture_Contrast_AGP_10_02
Cytoplasm_Texture_Contrast_AGP_10_03
Cytoplasm_Texture_Contrast_AGP_20_00
Cytoplasm_Texture_Contrast_AGP_20_01
Cytoplasm_Texture_Contrast_AGP_20_02
Cytoplasm_Texture_Contrast_AGP_20_03
Cytoplasm_Texture_Contrast_AGP_5_00
Cytoplasm_Texture_Contrast_AGP_5_01
Cytoplasm_Texture_Contrast_AGP_5_02
Cytoplasm_Texture_Contrast_AGP_5_03
Cytoplasm_Texture_Contrast_Brightfield_10_00
Cytoplasm_Texture_Contrast_Brightfield_10_01
Cytoplasm_Texture_Contrast_Brightfield_10_02
Cytoplasm_Texture_Contrast_Brightfield_10_03
Cytoplasm_Texture_Contrast_Brightfield_20_00
Cytoplasm_Texture_Contrast_Brightfield_20_01

Cytoplasm_Texture_Contrast_Brightfield_20_02
Cytoplasm_Texture_Contrast_Brightfield_20_03
Cytoplasm_Texture_Contrast_Brightfield_5_00
Cytoplasm_Texture_Contrast_Brightfield_5_01
Cytoplasm_Texture_Contrast_Brightfield_5_02
Cytoplasm_Texture_Contrast_Brightfield_5_03
Cytoplasm_Texture_Contrast_DNA_10_00
Cytoplasm_Texture_Contrast_DNA_10_01
Cytoplasm_Texture_Contrast_DNA_10_02
Cytoplasm_Texture_Contrast_DNA_10_03
Cytoplasm_Texture_Contrast_DNA_20_00
Cytoplasm_Texture_Contrast_DNA_20_01
Cytoplasm_Texture_Contrast_DNA_20_02
Cytoplasm_Texture_Contrast_DNA_20_03
Cytoplasm_Texture_Contrast_DNA_5_00
Cytoplasm_Texture_Contrast_DNA_5_01
Cytoplasm_Texture_Contrast_DNA_5_02
Cytoplasm_Texture_Contrast_DNA_5_03
Cytoplasm_Texture_Contrast_ER_10_00
Cytoplasm_Texture_Contrast_ER_10_01
Cytoplasm_Texture_Contrast_ER_10_02
Cytoplasm_Texture_Contrast_ER_10_03
Cytoplasm_Texture_Contrast_ER_20_00
Cytoplasm_Texture_Contrast_ER_20_01
Cytoplasm_Texture_Contrast_ER_20_02
Cytoplasm_Texture_Contrast_ER_20_03
Cytoplasm_Texture_Contrast_ER_5_00
Cytoplasm_Texture_Contrast_ER_5_01
Cytoplasm_Texture_Contrast_ER_5_02
Cytoplasm_Texture_Contrast_ER_5_03
Cytoplasm_Texture_Contrast_Mito_10_00
Cytoplasm_Texture_Contrast_Mito_10_01
Cytoplasm_Texture_Contrast_Mito_10_02
Cytoplasm_Texture_Contrast_Mito_10_03
Cytoplasm_Texture_Contrast_Mito_20_00
Cytoplasm_Texture_Contrast_Mito_20_01
Cytoplasm_Texture_Contrast_Mito_20_02
Cytoplasm_Texture_Contrast_Mito_20_03
Cytoplasm_Texture_Contrast_Mito_5_00

Cytoplasm_Texture_Contrast_Mito_5_01
Cytoplasm_Texture_Contrast_Mito_5_02
Cytoplasm_Texture_Contrast_Mito_5_03
Cytoplasm_Texture_Contrast_RNA_10_00
Cytoplasm_Texture_Contrast_RNA_10_01
Cytoplasm_Texture_Contrast_RNA_10_02
Cytoplasm_Texture_Contrast_RNA_10_03
Cytoplasm_Texture_Contrast_RNA_20_00
Cytoplasm_Texture_Contrast_RNA_20_01
Cytoplasm_Texture_Contrast_RNA_20_02
Cytoplasm_Texture_Contrast_RNA_20_03
Cytoplasm_Texture_Contrast_RNA_5_00
Cytoplasm_Texture_Contrast_RNA_5_01
Cytoplasm_Texture_Contrast_RNA_5_02
Cytoplasm_Texture_Contrast_RNA_5_03
Cytoplasm_Texture_DifferenceEntropy_AGP_10_00
Cytoplasm_Texture_DifferenceEntropy_AGP_10_01
Cytoplasm_Texture_DifferenceEntropy_AGP_10_02
Cytoplasm_Texture_DifferenceEntropy_AGP_10_03
Cytoplasm_Texture_DifferenceEntropy_AGP_20_00
Cytoplasm_Texture_DifferenceEntropy_AGP_20_01
Cytoplasm_Texture_DifferenceEntropy_AGP_20_02
Cytoplasm_Texture_DifferenceEntropy_AGP_20_03
Cytoplasm_Texture_DifferenceEntropy_AGP_5_00
Cytoplasm_Texture_DifferenceEntropy_AGP_5_01
Cytoplasm_Texture_DifferenceEntropy_AGP_5_02
Cytoplasm_Texture_DifferenceEntropy_AGP_5_03
Cytoplasm_Texture_DifferenceEntropy_Brightfield_10_00
Cytoplasm_Texture_DifferenceEntropy_Brightfield_10_01
Cytoplasm_Texture_DifferenceEntropy_Brightfield_10_02
Cytoplasm_Texture_DifferenceEntropy_Brightfield_10_03
Cytoplasm_Texture_DifferenceEntropy_Brightfield_20_00
Cytoplasm_Texture_DifferenceEntropy_Brightfield_20_01
Cytoplasm_Texture_DifferenceEntropy_Brightfield_20_02
Cytoplasm_Texture_DifferenceEntropy_Brightfield_20_03
Cytoplasm_Texture_DifferenceEntropy_Brightfield_5_00
Cytoplasm_Texture_DifferenceEntropy_Brightfield_5_01
Cytoplasm_Texture_DifferenceEntropy_Brightfield_5_02
Cytoplasm_Texture_DifferenceEntropy_Brightfield_5_03

Cytoplasm_Texture_DifferenceEntropy_DNA_10_00
Cytoplasm_Texture_DifferenceEntropy_DNA_10_01
Cytoplasm_Texture_DifferenceEntropy_DNA_10_02
Cytoplasm_Texture_DifferenceEntropy_DNA_10_03
Cytoplasm_Texture_DifferenceEntropy_DNA_20_00
Cytoplasm_Texture_DifferenceEntropy_DNA_20_01
Cytoplasm_Texture_DifferenceEntropy_DNA_20_02
Cytoplasm_Texture_DifferenceEntropy_DNA_20_03
Cytoplasm_Texture_DifferenceEntropy_DNA_5_00
Cytoplasm_Texture_DifferenceEntropy_DNA_5_01
Cytoplasm_Texture_DifferenceEntropy_DNA_5_02
Cytoplasm_Texture_DifferenceEntropy_DNA_5_03
Cytoplasm_Texture_DifferenceEntropy_ER_10_00
Cytoplasm_Texture_DifferenceEntropy_ER_10_01
Cytoplasm_Texture_DifferenceEntropy_ER_10_02
Cytoplasm_Texture_DifferenceEntropy_ER_10_03
Cytoplasm_Texture_DifferenceEntropy_ER_20_00
Cytoplasm_Texture_DifferenceEntropy_ER_20_01
Cytoplasm_Texture_DifferenceEntropy_ER_20_02
Cytoplasm_Texture_DifferenceEntropy_ER_20_03
Cytoplasm_Texture_DifferenceEntropy_ER_5_00
Cytoplasm_Texture_DifferenceEntropy_ER_5_01
Cytoplasm_Texture_DifferenceEntropy_ER_5_02
Cytoplasm_Texture_DifferenceEntropy_ER_5_03
Cytoplasm_Texture_DifferenceEntropy_Mito_10_00
Cytoplasm_Texture_DifferenceEntropy_Mito_10_01
Cytoplasm_Texture_DifferenceEntropy_Mito_10_02
Cytoplasm_Texture_DifferenceEntropy_Mito_10_03
Cytoplasm_Texture_DifferenceEntropy_Mito_20_00
Cytoplasm_Texture_DifferenceEntropy_Mito_20_01
Cytoplasm_Texture_DifferenceEntropy_Mito_20_02
Cytoplasm_Texture_DifferenceEntropy_Mito_20_03
Cytoplasm_Texture_DifferenceEntropy_Mito_5_00
Cytoplasm_Texture_DifferenceEntropy_Mito_5_01
Cytoplasm_Texture_DifferenceEntropy_Mito_5_02
Cytoplasm_Texture_DifferenceEntropy_Mito_5_03
Cytoplasm_Texture_DifferenceEntropy_RNA_10_00
Cytoplasm_Texture_DifferenceEntropy_RNA_10_01
Cytoplasm_Texture_DifferenceEntropy_RNA_10_02

Cytoplasm_Texture_DifferenceEntropy_RNA_10_03
Cytoplasm_Texture_DifferenceEntropy_RNA_20_00
Cytoplasm_Texture_DifferenceEntropy_RNA_20_01
Cytoplasm_Texture_DifferenceEntropy_RNA_20_02
Cytoplasm_Texture_DifferenceEntropy_RNA_20_03
Cytoplasm_Texture_DifferenceEntropy_RNA_5_00
Cytoplasm_Texture_DifferenceEntropy_RNA_5_01
Cytoplasm_Texture_DifferenceEntropy_RNA_5_02
Cytoplasm_Texture_DifferenceEntropy_RNA_5_03
Cytoplasm_Texture_DifferenceVariance_AGP_10_00
Cytoplasm_Texture_DifferenceVariance_AGP_10_01
Cytoplasm_Texture_DifferenceVariance_AGP_10_02
Cytoplasm_Texture_DifferenceVariance_AGP_10_03
Cytoplasm_Texture_DifferenceVariance_AGP_20_00
Cytoplasm_Texture_DifferenceVariance_AGP_20_01
Cytoplasm_Texture_DifferenceVariance_AGP_20_02
Cytoplasm_Texture_DifferenceVariance_AGP_20_03
Cytoplasm_Texture_DifferenceVariance_AGP_5_00
Cytoplasm_Texture_DifferenceVariance_AGP_5_01
Cytoplasm_Texture_DifferenceVariance_AGP_5_02
Cytoplasm_Texture_DifferenceVariance_AGP_5_03
Cytoplasm_Texture_DifferenceVariance_Brightfield_10_00
Cytoplasm_Texture_DifferenceVariance_Brightfield_10_01
Cytoplasm_Texture_DifferenceVariance_Brightfield_10_02
Cytoplasm_Texture_DifferenceVariance_Brightfield_10_03
Cytoplasm_Texture_DifferenceVariance_Brightfield_20_00
Cytoplasm_Texture_DifferenceVariance_Brightfield_20_01
Cytoplasm_Texture_DifferenceVariance_Brightfield_20_02
Cytoplasm_Texture_DifferenceVariance_Brightfield_20_03
Cytoplasm_Texture_DifferenceVariance_Brightfield_5_00
Cytoplasm_Texture_DifferenceVariance_Brightfield_5_01
Cytoplasm_Texture_DifferenceVariance_Brightfield_5_02
Cytoplasm_Texture_DifferenceVariance_Brightfield_5_03
Cytoplasm_Texture_DifferenceVariance_DNA_10_00
Cytoplasm_Texture_DifferenceVariance_DNA_10_01
Cytoplasm_Texture_DifferenceVariance_DNA_10_02
Cytoplasm_Texture_DifferenceVariance_DNA_10_03
Cytoplasm_Texture_DifferenceVariance_DNA_20_00
Cytoplasm_Texture_DifferenceVariance_DNA_20_01

Cytoplasm_Texture_DifferenceVariance_DNA_20_02
Cytoplasm_Texture_DifferenceVariance_DNA_20_03
Cytoplasm_Texture_DifferenceVariance_DNA_5_00
Cytoplasm_Texture_DifferenceVariance_DNA_5_01
Cytoplasm_Texture_DifferenceVariance_DNA_5_02
Cytoplasm_Texture_DifferenceVariance_DNA_5_03
Cytoplasm_Texture_DifferenceVariance_ER_10_00
Cytoplasm_Texture_DifferenceVariance_ER_10_01
Cytoplasm_Texture_DifferenceVariance_ER_10_02
Cytoplasm_Texture_DifferenceVariance_ER_10_03
Cytoplasm_Texture_DifferenceVariance_ER_20_00
Cytoplasm_Texture_DifferenceVariance_ER_20_01
Cytoplasm_Texture_DifferenceVariance_ER_20_02
Cytoplasm_Texture_DifferenceVariance_ER_20_03
Cytoplasm_Texture_DifferenceVariance_ER_5_00
Cytoplasm_Texture_DifferenceVariance_ER_5_01
Cytoplasm_Texture_DifferenceVariance_ER_5_02
Cytoplasm_Texture_DifferenceVariance_ER_5_03
Cytoplasm_Texture_DifferenceVariance_Mito_10_00
Cytoplasm_Texture_DifferenceVariance_Mito_10_01
Cytoplasm_Texture_DifferenceVariance_Mito_10_02
Cytoplasm_Texture_DifferenceVariance_Mito_10_03
Cytoplasm_Texture_DifferenceVariance_Mito_20_00
Cytoplasm_Texture_DifferenceVariance_Mito_20_01
Cytoplasm_Texture_DifferenceVariance_Mito_20_02
Cytoplasm_Texture_DifferenceVariance_Mito_20_03
Cytoplasm_Texture_DifferenceVariance_Mito_5_00
Cytoplasm_Texture_DifferenceVariance_Mito_5_01
Cytoplasm_Texture_DifferenceVariance_Mito_5_02
Cytoplasm_Texture_DifferenceVariance_Mito_5_03
Cytoplasm_Texture_DifferenceVariance_RNA_10_00
Cytoplasm_Texture_DifferenceVariance_RNA_10_01
Cytoplasm_Texture_DifferenceVariance_RNA_10_02
Cytoplasm_Texture_DifferenceVariance_RNA_10_03
Cytoplasm_Texture_DifferenceVariance_RNA_20_00
Cytoplasm_Texture_DifferenceVariance_RNA_20_01
Cytoplasm_Texture_DifferenceVariance_RNA_20_02
Cytoplasm_Texture_DifferenceVariance_RNA_20_03
Cytoplasm_Texture_DifferenceVariance_RNA_5_00

Cytoplasm_Texture_DifferenceVariance_RNA_5_01
Cytoplasm_Texture_DifferenceVariance_RNA_5_02
Cytoplasm_Texture_DifferenceVariance_RNA_5_03
Cytoplasm_Texture_Entropy_AGP_10_00
Cytoplasm_Texture_Entropy_AGP_10_01
Cytoplasm_Texture_Entropy_AGP_10_02
Cytoplasm_Texture_Entropy_AGP_10_03
Cytoplasm_Texture_Entropy_AGP_20_00
Cytoplasm_Texture_Entropy_AGP_20_01
Cytoplasm_Texture_Entropy_AGP_20_02
Cytoplasm_Texture_Entropy_AGP_20_03
Cytoplasm_Texture_Entropy_AGP_5_00
Cytoplasm_Texture_Entropy_AGP_5_01
Cytoplasm_Texture_Entropy_AGP_5_02
Cytoplasm_Texture_Entropy_AGP_5_03
Cytoplasm_Texture_Entropy_Brightfield_10_00
Cytoplasm_Texture_Entropy_Brightfield_10_01
Cytoplasm_Texture_Entropy_Brightfield_10_02
Cytoplasm_Texture_Entropy_Brightfield_10_03
Cytoplasm_Texture_Entropy_Brightfield_20_00
Cytoplasm_Texture_Entropy_Brightfield_20_01
Cytoplasm_Texture_Entropy_Brightfield_20_02
Cytoplasm_Texture_Entropy_Brightfield_20_03
Cytoplasm_Texture_Entropy_Brightfield_5_00
Cytoplasm_Texture_Entropy_Brightfield_5_01
Cytoplasm_Texture_Entropy_Brightfield_5_02
Cytoplasm_Texture_Entropy_Brightfield_5_03
Cytoplasm_Texture_Entropy_DNA_10_00
Cytoplasm_Texture_Entropy_DNA_10_01
Cytoplasm_Texture_Entropy_DNA_10_02
Cytoplasm_Texture_Entropy_DNA_10_03
Cytoplasm_Texture_Entropy_DNA_20_00
Cytoplasm_Texture_Entropy_DNA_20_01
Cytoplasm_Texture_Entropy_DNA_20_02
Cytoplasm_Texture_Entropy_DNA_20_03
Cytoplasm_Texture_Entropy_DNA_5_00
Cytoplasm_Texture_Entropy_DNA_5_01
Cytoplasm_Texture_Entropy_DNA_5_02
Cytoplasm_Texture_Entropy_DNA_5_03

Cytoplasm_Texture_Entropy_ER_10_00
Cytoplasm_Texture_Entropy_ER_10_01
Cytoplasm_Texture_Entropy_ER_10_02
Cytoplasm_Texture_Entropy_ER_10_03
Cytoplasm_Texture_Entropy_ER_20_00
Cytoplasm_Texture_Entropy_ER_20_01
Cytoplasm_Texture_Entropy_ER_20_02
Cytoplasm_Texture_Entropy_ER_20_03
Cytoplasm_Texture_Entropy_ER_5_00
Cytoplasm_Texture_Entropy_ER_5_01
Cytoplasm_Texture_Entropy_ER_5_02
Cytoplasm_Texture_Entropy_ER_5_03
Cytoplasm_Texture_Entropy_Mito_10_00
Cytoplasm_Texture_Entropy_Mito_10_01
Cytoplasm_Texture_Entropy_Mito_10_02
Cytoplasm_Texture_Entropy_Mito_10_03
Cytoplasm_Texture_Entropy_Mito_20_00
Cytoplasm_Texture_Entropy_Mito_20_01
Cytoplasm_Texture_Entropy_Mito_20_02
Cytoplasm_Texture_Entropy_Mito_20_03
Cytoplasm_Texture_Entropy_Mito_5_00
Cytoplasm_Texture_Entropy_Mito_5_01
Cytoplasm_Texture_Entropy_Mito_5_02
Cytoplasm_Texture_Entropy_Mito_5_03
Cytoplasm_Texture_Entropy_RNA_10_00
Cytoplasm_Texture_Entropy_RNA_10_01
Cytoplasm_Texture_Entropy_RNA_10_02
Cytoplasm_Texture_Entropy_RNA_10_03
Cytoplasm_Texture_Entropy_RNA_20_00
Cytoplasm_Texture_Entropy_RNA_20_01
Cytoplasm_Texture_Entropy_RNA_20_02
Cytoplasm_Texture_Entropy_RNA_20_03
Cytoplasm_Texture_Entropy_RNA_5_00
Cytoplasm_Texture_Entropy_RNA_5_01
Cytoplasm_Texture_Entropy_RNA_5_02
Cytoplasm_Texture_Entropy_RNA_5_03
Cytoplasm_Texture_InfoMeas1_AGP_10_00
Cytoplasm_Texture_InfoMeas1_AGP_10_01
Cytoplasm_Texture_InfoMeas1_AGP_10_02

Cytoplasm_Texture_InfoMeas1_AGP_10_03
Cytoplasm_Texture_InfoMeas1_AGP_20_00
Cytoplasm_Texture_InfoMeas1_AGP_20_01
Cytoplasm_Texture_InfoMeas1_AGP_20_02
Cytoplasm_Texture_InfoMeas1_AGP_20_03
Cytoplasm_Texture_InfoMeas1_AGP_5_00
Cytoplasm_Texture_InfoMeas1_AGP_5_01
Cytoplasm_Texture_InfoMeas1_AGP_5_02
Cytoplasm_Texture_InfoMeas1_AGP_5_03
Cytoplasm_Texture_InfoMeas1_Brightfield_10_00
Cytoplasm_Texture_InfoMeas1_Brightfield_10_01
Cytoplasm_Texture_InfoMeas1_Brightfield_10_02
Cytoplasm_Texture_InfoMeas1_Brightfield_10_03
Cytoplasm_Texture_InfoMeas1_Brightfield_20_00
Cytoplasm_Texture_InfoMeas1_Brightfield_20_01
Cytoplasm_Texture_InfoMeas1_Brightfield_20_02
Cytoplasm_Texture_InfoMeas1_Brightfield_20_03
Cytoplasm_Texture_InfoMeas1_Brightfield_5_00
Cytoplasm_Texture_InfoMeas1_Brightfield_5_01
Cytoplasm_Texture_InfoMeas1_Brightfield_5_02
Cytoplasm_Texture_InfoMeas1_Brightfield_5_03
Cytoplasm_Texture_InfoMeas1_DNA_10_00
Cytoplasm_Texture_InfoMeas1_DNA_10_01
Cytoplasm_Texture_InfoMeas1_DNA_10_02
Cytoplasm_Texture_InfoMeas1_DNA_10_03
Cytoplasm_Texture_InfoMeas1_DNA_20_00
Cytoplasm_Texture_InfoMeas1_DNA_20_01
Cytoplasm_Texture_InfoMeas1_DNA_20_02
Cytoplasm_Texture_InfoMeas1_DNA_20_03
Cytoplasm_Texture_InfoMeas1_DNA_5_00
Cytoplasm_Texture_InfoMeas1_DNA_5_01
Cytoplasm_Texture_InfoMeas1_DNA_5_02
Cytoplasm_Texture_InfoMeas1_DNA_5_03
Cytoplasm_Texture_InfoMeas1_ER_10_00
Cytoplasm_Texture_InfoMeas1_ER_10_01
Cytoplasm_Texture_InfoMeas1_ER_10_02
Cytoplasm_Texture_InfoMeas1_ER_10_03
Cytoplasm_Texture_InfoMeas1_ER_20_00
Cytoplasm_Texture_InfoMeas1_ER_20_01

Cytoplasm_Texture_InfoMeas1_ER_20_02
Cytoplasm_Texture_InfoMeas1_ER_20_03
Cytoplasm_Texture_InfoMeas1_ER_5_00
Cytoplasm_Texture_InfoMeas1_ER_5_01
Cytoplasm_Texture_InfoMeas1_ER_5_02
Cytoplasm_Texture_InfoMeas1_ER_5_03
Cytoplasm_Texture_InfoMeas1_Mito_10_00
Cytoplasm_Texture_InfoMeas1_Mito_10_01
Cytoplasm_Texture_InfoMeas1_Mito_10_02
Cytoplasm_Texture_InfoMeas1_Mito_10_03
Cytoplasm_Texture_InfoMeas1_Mito_20_00
Cytoplasm_Texture_InfoMeas1_Mito_20_01
Cytoplasm_Texture_InfoMeas1_Mito_20_02
Cytoplasm_Texture_InfoMeas1_Mito_20_03
Cytoplasm_Texture_InfoMeas1_Mito_5_00
Cytoplasm_Texture_InfoMeas1_Mito_5_01
Cytoplasm_Texture_InfoMeas1_Mito_5_02
Cytoplasm_Texture_InfoMeas1_Mito_5_03
Cytoplasm_Texture_InfoMeas1_RNA_10_00
Cytoplasm_Texture_InfoMeas1_RNA_10_01
Cytoplasm_Texture_InfoMeas1_RNA_10_02
Cytoplasm_Texture_InfoMeas1_RNA_10_03
Cytoplasm_Texture_InfoMeas1_RNA_20_00
Cytoplasm_Texture_InfoMeas1_RNA_20_01
Cytoplasm_Texture_InfoMeas1_RNA_20_02
Cytoplasm_Texture_InfoMeas1_RNA_20_03
Cytoplasm_Texture_InfoMeas1_RNA_5_00
Cytoplasm_Texture_InfoMeas1_RNA_5_01
Cytoplasm_Texture_InfoMeas1_RNA_5_02
Cytoplasm_Texture_InfoMeas1_RNA_5_03
Cytoplasm_Texture_InfoMeas2_AGP_10_00
Cytoplasm_Texture_InfoMeas2_AGP_10_01
Cytoplasm_Texture_InfoMeas2_AGP_10_02
Cytoplasm_Texture_InfoMeas2_AGP_10_03
Cytoplasm_Texture_InfoMeas2_AGP_20_00
Cytoplasm_Texture_InfoMeas2_AGP_20_01
Cytoplasm_Texture_InfoMeas2_AGP_20_02
Cytoplasm_Texture_InfoMeas2_AGP_20_03
Cytoplasm_Texture_InfoMeas2_AGP_5_00

Cytoplasm_Texture_InfoMeas2_AGP_5_01
Cytoplasm_Texture_InfoMeas2_AGP_5_02
Cytoplasm_Texture_InfoMeas2_AGP_5_03
Cytoplasm_Texture_InfoMeas2_Brightfield_10_00
Cytoplasm_Texture_InfoMeas2_Brightfield_10_01
Cytoplasm_Texture_InfoMeas2_Brightfield_10_02
Cytoplasm_Texture_InfoMeas2_Brightfield_10_03
Cytoplasm_Texture_InfoMeas2_Brightfield_20_00
Cytoplasm_Texture_InfoMeas2_Brightfield_20_01
Cytoplasm_Texture_InfoMeas2_Brightfield_20_02
Cytoplasm_Texture_InfoMeas2_Brightfield_20_03
Cytoplasm_Texture_InfoMeas2_Brightfield_5_00
Cytoplasm_Texture_InfoMeas2_Brightfield_5_01
Cytoplasm_Texture_InfoMeas2_Brightfield_5_02
Cytoplasm_Texture_InfoMeas2_Brightfield_5_03
Cytoplasm_Texture_InfoMeas2_DNA_10_00
Cytoplasm_Texture_InfoMeas2_DNA_10_01
Cytoplasm_Texture_InfoMeas2_DNA_10_02
Cytoplasm_Texture_InfoMeas2_DNA_10_03
Cytoplasm_Texture_InfoMeas2_DNA_20_00
Cytoplasm_Texture_InfoMeas2_DNA_20_01
Cytoplasm_Texture_InfoMeas2_DNA_20_02
Cytoplasm_Texture_InfoMeas2_DNA_20_03
Cytoplasm_Texture_InfoMeas2_DNA_5_00
Cytoplasm_Texture_InfoMeas2_DNA_5_01
Cytoplasm_Texture_InfoMeas2_DNA_5_02
Cytoplasm_Texture_InfoMeas2_DNA_5_03
Cytoplasm_Texture_InfoMeas2_ER_10_00
Cytoplasm_Texture_InfoMeas2_ER_10_01
Cytoplasm_Texture_InfoMeas2_ER_10_02
Cytoplasm_Texture_InfoMeas2_ER_10_03
Cytoplasm_Texture_InfoMeas2_ER_20_00
Cytoplasm_Texture_InfoMeas2_ER_20_01
Cytoplasm_Texture_InfoMeas2_ER_20_02
Cytoplasm_Texture_InfoMeas2_ER_20_03
Cytoplasm_Texture_InfoMeas2_ER_5_00
Cytoplasm_Texture_InfoMeas2_ER_5_01
Cytoplasm_Texture_InfoMeas2_ER_5_02
Cytoplasm_Texture_InfoMeas2_ER_5_03

Cytoplasm_Texture_InfoMeas2_Mito_10_00
Cytoplasm_Texture_InfoMeas2_Mito_10_01
Cytoplasm_Texture_InfoMeas2_Mito_10_02
Cytoplasm_Texture_InfoMeas2_Mito_10_03
Cytoplasm_Texture_InfoMeas2_Mito_20_00
Cytoplasm_Texture_InfoMeas2_Mito_20_01
Cytoplasm_Texture_InfoMeas2_Mito_20_02
Cytoplasm_Texture_InfoMeas2_Mito_20_03
Cytoplasm_Texture_InfoMeas2_Mito_5_00
Cytoplasm_Texture_InfoMeas2_Mito_5_01
Cytoplasm_Texture_InfoMeas2_Mito_5_02
Cytoplasm_Texture_InfoMeas2_Mito_5_03
Cytoplasm_Texture_InfoMeas2_RNA_10_00
Cytoplasm_Texture_InfoMeas2_RNA_10_01
Cytoplasm_Texture_InfoMeas2_RNA_10_02
Cytoplasm_Texture_InfoMeas2_RNA_10_03
Cytoplasm_Texture_InfoMeas2_RNA_20_00
Cytoplasm_Texture_InfoMeas2_RNA_20_01
Cytoplasm_Texture_InfoMeas2_RNA_20_02
Cytoplasm_Texture_InfoMeas2_RNA_20_03
Cytoplasm_Texture_InfoMeas2_RNA_5_00
Cytoplasm_Texture_InfoMeas2_RNA_5_01
Cytoplasm_Texture_InfoMeas2_RNA_5_02
Cytoplasm_Texture_InfoMeas2_RNA_5_03
Cytoplasm_Texture_InverseDifferenceMoment_AGP_10_00
Cytoplasm_Texture_InverseDifferenceMoment_AGP_10_01
Cytoplasm_Texture_InverseDifferenceMoment_AGP_10_02
Cytoplasm_Texture_InverseDifferenceMoment_AGP_10_03
Cytoplasm_Texture_InverseDifferenceMoment_AGP_20_00
Cytoplasm_Texture_InverseDifferenceMoment_AGP_20_01
Cytoplasm_Texture_InverseDifferenceMoment_AGP_20_02
Cytoplasm_Texture_InverseDifferenceMoment_AGP_20_03
Cytoplasm_Texture_InverseDifferenceMoment_AGP_5_00
Cytoplasm_Texture_InverseDifferenceMoment_AGP_5_01
Cytoplasm_Texture_InverseDifferenceMoment_AGP_5_02
Cytoplasm_Texture_InverseDifferenceMoment_AGP_5_03
Cytoplasm_Texture_InverseDifferenceMoment_Brightfield_10_00
Cytoplasm_Texture_InverseDifferenceMoment_Brightfield_10_01
Cytoplasm_Texture_InverseDifferenceMoment_Brightfield_10_02

Cytoplasm_Texture_InverseDifferenceMoment_Brightfield_10_03
Cytoplasm_Texture_InverseDifferenceMoment_Brightfield_20_00
Cytoplasm_Texture_InverseDifferenceMoment_Brightfield_20_01
Cytoplasm_Texture_InverseDifferenceMoment_Brightfield_20_02
Cytoplasm_Texture_InverseDifferenceMoment_Brightfield_20_03
Cytoplasm_Texture_InverseDifferenceMoment_Brightfield_5_00
Cytoplasm_Texture_InverseDifferenceMoment_Brightfield_5_01
Cytoplasm_Texture_InverseDifferenceMoment_Brightfield_5_02
Cytoplasm_Texture_InverseDifferenceMoment_Brightfield_5_03
Cytoplasm_Texture_InverseDifferenceMoment_DNA_10_00
Cytoplasm_Texture_InverseDifferenceMoment_DNA_10_01
Cytoplasm_Texture_InverseDifferenceMoment_DNA_10_02
Cytoplasm_Texture_InverseDifferenceMoment_DNA_10_03
Cytoplasm_Texture_InverseDifferenceMoment_DNA_20_00
Cytoplasm_Texture_InverseDifferenceMoment_DNA_20_01
Cytoplasm_Texture_InverseDifferenceMoment_DNA_20_02
Cytoplasm_Texture_InverseDifferenceMoment_DNA_20_03
Cytoplasm_Texture_InverseDifferenceMoment_DNA_5_00
Cytoplasm_Texture_InverseDifferenceMoment_DNA_5_01
Cytoplasm_Texture_InverseDifferenceMoment_DNA_5_02
Cytoplasm_Texture_InverseDifferenceMoment_DNA_5_03
Cytoplasm_Texture_InverseDifferenceMoment_ER_10_00
Cytoplasm_Texture_InverseDifferenceMoment_ER_10_01
Cytoplasm_Texture_InverseDifferenceMoment_ER_10_02
Cytoplasm_Texture_InverseDifferenceMoment_ER_10_03
Cytoplasm_Texture_InverseDifferenceMoment_ER_20_00
Cytoplasm_Texture_InverseDifferenceMoment_ER_20_01
Cytoplasm_Texture_InverseDifferenceMoment_ER_20_02
Cytoplasm_Texture_InverseDifferenceMoment_ER_20_03
Cytoplasm_Texture_InverseDifferenceMoment_ER_5_00
Cytoplasm_Texture_InverseDifferenceMoment_ER_5_01
Cytoplasm_Texture_InverseDifferenceMoment_ER_5_02
Cytoplasm_Texture_InverseDifferenceMoment_ER_5_03
Cytoplasm_Texture_InverseDifferenceMoment_Mito_10_00
Cytoplasm_Texture_InverseDifferenceMoment_Mito_10_01
Cytoplasm_Texture_InverseDifferenceMoment_Mito_10_02
Cytoplasm_Texture_InverseDifferenceMoment_Mito_10_03
Cytoplasm_Texture_InverseDifferenceMoment_Mito_20_00
Cytoplasm_Texture_InverseDifferenceMoment_Mito_20_01

Cytoplasm_Texture_InverseDifferenceMoment_Mito_20_02
Cytoplasm_Texture_InverseDifferenceMoment_Mito_20_03
Cytoplasm_Texture_InverseDifferenceMoment_Mito_5_00
Cytoplasm_Texture_InverseDifferenceMoment_Mito_5_01
Cytoplasm_Texture_InverseDifferenceMoment_Mito_5_02
Cytoplasm_Texture_InverseDifferenceMoment_Mito_5_03
Cytoplasm_Texture_InverseDifferenceMoment_RNA_10_00
Cytoplasm_Texture_InverseDifferenceMoment_RNA_10_01
Cytoplasm_Texture_InverseDifferenceMoment_RNA_10_02
Cytoplasm_Texture_InverseDifferenceMoment_RNA_10_03
Cytoplasm_Texture_InverseDifferenceMoment_RNA_20_00
Cytoplasm_Texture_InverseDifferenceMoment_RNA_20_01
Cytoplasm_Texture_InverseDifferenceMoment_RNA_20_02
Cytoplasm_Texture_InverseDifferenceMoment_RNA_20_03
Cytoplasm_Texture_InverseDifferenceMoment_RNA_5_00
Cytoplasm_Texture_InverseDifferenceMoment_RNA_5_01
Cytoplasm_Texture_InverseDifferenceMoment_RNA_5_02
Cytoplasm_Texture_InverseDifferenceMoment_RNA_5_03
Cytoplasm_Texture_SumAverage_AGP_10_00
Cytoplasm_Texture_SumAverage_AGP_10_01
Cytoplasm_Texture_SumAverage_AGP_10_02
Cytoplasm_Texture_SumAverage_AGP_10_03
Cytoplasm_Texture_SumAverage_AGP_20_00
Cytoplasm_Texture_SumAverage_AGP_20_01
Cytoplasm_Texture_SumAverage_AGP_20_02
Cytoplasm_Texture_SumAverage_AGP_20_03
Cytoplasm_Texture_SumAverage_AGP_5_00
Cytoplasm_Texture_SumAverage_AGP_5_01
Cytoplasm_Texture_SumAverage_AGP_5_02
Cytoplasm_Texture_SumAverage_AGP_5_03
Cytoplasm_Texture_SumAverage_Brightfield_10_00
Cytoplasm_Texture_SumAverage_Brightfield_10_01
Cytoplasm_Texture_SumAverage_Brightfield_10_02
Cytoplasm_Texture_SumAverage_Brightfield_10_03
Cytoplasm_Texture_SumAverage_Brightfield_20_00
Cytoplasm_Texture_SumAverage_Brightfield_20_01
Cytoplasm_Texture_SumAverage_Brightfield_20_02
Cytoplasm_Texture_SumAverage_Brightfield_20_03
Cytoplasm_Texture_SumAverage_Brightfield_5_00

Cytoplasm_Texture_SumAverage_Brightfield_5_01
Cytoplasm_Texture_SumAverage_Brightfield_5_02
Cytoplasm_Texture_SumAverage_Brightfield_5_03
Cytoplasm_Texture_SumAverage_DNA_10_00
Cytoplasm_Texture_SumAverage_DNA_10_01
Cytoplasm_Texture_SumAverage_DNA_10_02
Cytoplasm_Texture_SumAverage_DNA_10_03
Cytoplasm_Texture_SumAverage_DNA_20_00
Cytoplasm_Texture_SumAverage_DNA_20_01
Cytoplasm_Texture_SumAverage_DNA_20_02
Cytoplasm_Texture_SumAverage_DNA_20_03
Cytoplasm_Texture_SumAverage_DNA_5_00
Cytoplasm_Texture_SumAverage_DNA_5_01
Cytoplasm_Texture_SumAverage_DNA_5_02
Cytoplasm_Texture_SumAverage_DNA_5_03
Cytoplasm_Texture_SumAverage_ER_10_00
Cytoplasm_Texture_SumAverage_ER_10_01
Cytoplasm_Texture_SumAverage_ER_10_02
Cytoplasm_Texture_SumAverage_ER_10_03
Cytoplasm_Texture_SumAverage_ER_20_00
Cytoplasm_Texture_SumAverage_ER_20_01
Cytoplasm_Texture_SumAverage_ER_20_02
Cytoplasm_Texture_SumAverage_ER_20_03
Cytoplasm_Texture_SumAverage_ER_5_00
Cytoplasm_Texture_SumAverage_ER_5_01
Cytoplasm_Texture_SumAverage_ER_5_02
Cytoplasm_Texture_SumAverage_ER_5_03
Cytoplasm_Texture_SumAverage_Mito_10_00
Cytoplasm_Texture_SumAverage_Mito_10_01
Cytoplasm_Texture_SumAverage_Mito_10_02
Cytoplasm_Texture_SumAverage_Mito_10_03
Cytoplasm_Texture_SumAverage_Mito_20_00
Cytoplasm_Texture_SumAverage_Mito_20_01
Cytoplasm_Texture_SumAverage_Mito_20_02
Cytoplasm_Texture_SumAverage_Mito_20_03
Cytoplasm_Texture_SumAverage_Mito_5_00
Cytoplasm_Texture_SumAverage_Mito_5_01
Cytoplasm_Texture_SumAverage_Mito_5_02
Cytoplasm_Texture_SumAverage_Mito_5_03

Cytoplasm_Texture_SumAverage_RNA_10_00
Cytoplasm_Texture_SumAverage_RNA_10_01
Cytoplasm_Texture_SumAverage_RNA_10_02
Cytoplasm_Texture_SumAverage_RNA_10_03
Cytoplasm_Texture_SumAverage_RNA_20_00
Cytoplasm_Texture_SumAverage_RNA_20_01
Cytoplasm_Texture_SumAverage_RNA_20_02
Cytoplasm_Texture_SumAverage_RNA_20_03
Cytoplasm_Texture_SumAverage_RNA_5_00
Cytoplasm_Texture_SumAverage_RNA_5_01
Cytoplasm_Texture_SumAverage_RNA_5_02
Cytoplasm_Texture_SumAverage_RNA_5_03
Cytoplasm_Texture_SumEntropy_AGP_10_00
Cytoplasm_Texture_SumEntropy_AGP_10_01
Cytoplasm_Texture_SumEntropy_AGP_10_02
Cytoplasm_Texture_SumEntropy_AGP_10_03
Cytoplasm_Texture_SumEntropy_AGP_20_00
Cytoplasm_Texture_SumEntropy_AGP_20_01
Cytoplasm_Texture_SumEntropy_AGP_20_02
Cytoplasm_Texture_SumEntropy_AGP_20_03
Cytoplasm_Texture_SumEntropy_AGP_5_00
Cytoplasm_Texture_SumEntropy_AGP_5_01
Cytoplasm_Texture_SumEntropy_AGP_5_02
Cytoplasm_Texture_SumEntropy_AGP_5_03
Cytoplasm_Texture_SumEntropy_Brightfield_10_00
Cytoplasm_Texture_SumEntropy_Brightfield_10_01
Cytoplasm_Texture_SumEntropy_Brightfield_10_02
Cytoplasm_Texture_SumEntropy_Brightfield_10_03
Cytoplasm_Texture_SumEntropy_Brightfield_20_00
Cytoplasm_Texture_SumEntropy_Brightfield_20_01
Cytoplasm_Texture_SumEntropy_Brightfield_20_02
Cytoplasm_Texture_SumEntropy_Brightfield_20_03
Cytoplasm_Texture_SumEntropy_Brightfield_5_00
Cytoplasm_Texture_SumEntropy_Brightfield_5_01
Cytoplasm_Texture_SumEntropy_Brightfield_5_02
Cytoplasm_Texture_SumEntropy_Brightfield_5_03
Cytoplasm_Texture_SumEntropy_DNA_10_00
Cytoplasm_Texture_SumEntropy_DNA_10_01
Cytoplasm_Texture_SumEntropy_DNA_10_02

Cytoplasm_Texture_SumEntropy_DNA_10_03
Cytoplasm_Texture_SumEntropy_DNA_20_00
Cytoplasm_Texture_SumEntropy_DNA_20_01
Cytoplasm_Texture_SumEntropy_DNA_20_02
Cytoplasm_Texture_SumEntropy_DNA_20_03
Cytoplasm_Texture_SumEntropy_DNA_5_00
Cytoplasm_Texture_SumEntropy_DNA_5_01
Cytoplasm_Texture_SumEntropy_DNA_5_02
Cytoplasm_Texture_SumEntropy_DNA_5_03
Cytoplasm_Texture_SumEntropy_ER_10_00
Cytoplasm_Texture_SumEntropy_ER_10_01
Cytoplasm_Texture_SumEntropy_ER_10_02
Cytoplasm_Texture_SumEntropy_ER_10_03
Cytoplasm_Texture_SumEntropy_ER_20_00
Cytoplasm_Texture_SumEntropy_ER_20_01
Cytoplasm_Texture_SumEntropy_ER_20_02
Cytoplasm_Texture_SumEntropy_ER_20_03
Cytoplasm_Texture_SumEntropy_ER_5_00
Cytoplasm_Texture_SumEntropy_ER_5_01
Cytoplasm_Texture_SumEntropy_ER_5_02
Cytoplasm_Texture_SumEntropy_ER_5_03
Cytoplasm_Texture_SumEntropy_Mito_10_00
Cytoplasm_Texture_SumEntropy_Mito_10_01
Cytoplasm_Texture_SumEntropy_Mito_10_02
Cytoplasm_Texture_SumEntropy_Mito_10_03
Cytoplasm_Texture_SumEntropy_Mito_20_00
Cytoplasm_Texture_SumEntropy_Mito_20_01
Cytoplasm_Texture_SumEntropy_Mito_20_02
Cytoplasm_Texture_SumEntropy_Mito_20_03
Cytoplasm_Texture_SumEntropy_Mito_5_00
Cytoplasm_Texture_SumEntropy_Mito_5_01
Cytoplasm_Texture_SumEntropy_Mito_5_02
Cytoplasm_Texture_SumEntropy_Mito_5_03
Cytoplasm_Texture_SumEntropy_RNA_10_00
Cytoplasm_Texture_SumEntropy_RNA_10_01
Cytoplasm_Texture_SumEntropy_RNA_10_02
Cytoplasm_Texture_SumEntropy_RNA_10_03
Cytoplasm_Texture_SumEntropy_RNA_20_00
Cytoplasm_Texture_SumEntropy_RNA_20_01

Cytoplasm_Texture_SumEntropy_RNA_20_02
Cytoplasm_Texture_SumEntropy_RNA_20_03
Cytoplasm_Texture_SumEntropy_RNA_5_00
Cytoplasm_Texture_SumEntropy_RNA_5_01
Cytoplasm_Texture_SumEntropy_RNA_5_02
Cytoplasm_Texture_SumEntropy_RNA_5_03
Cytoplasm_Texture_SumVariance_AGP_10_00
Cytoplasm_Texture_SumVariance_AGP_10_01
Cytoplasm_Texture_SumVariance_AGP_10_02
Cytoplasm_Texture_SumVariance_AGP_10_03
Cytoplasm_Texture_SumVariance_AGP_20_00
Cytoplasm_Texture_SumVariance_AGP_20_01
Cytoplasm_Texture_SumVariance_AGP_20_02
Cytoplasm_Texture_SumVariance_AGP_20_03
Cytoplasm_Texture_SumVariance_AGP_5_00
Cytoplasm_Texture_SumVariance_AGP_5_01
Cytoplasm_Texture_SumVariance_AGP_5_02
Cytoplasm_Texture_SumVariance_AGP_5_03
Cytoplasm_Texture_SumVariance_Brightfield_10_00
Cytoplasm_Texture_SumVariance_Brightfield_10_01
Cytoplasm_Texture_SumVariance_Brightfield_10_02
Cytoplasm_Texture_SumVariance_Brightfield_10_03
Cytoplasm_Texture_SumVariance_Brightfield_20_00
Cytoplasm_Texture_SumVariance_Brightfield_20_01
Cytoplasm_Texture_SumVariance_Brightfield_20_02
Cytoplasm_Texture_SumVariance_Brightfield_20_03
Cytoplasm_Texture_SumVariance_Brightfield_5_00
Cytoplasm_Texture_SumVariance_Brightfield_5_01
Cytoplasm_Texture_SumVariance_Brightfield_5_02
Cytoplasm_Texture_SumVariance_Brightfield_5_03
Cytoplasm_Texture_SumVariance_DNA_10_00
Cytoplasm_Texture_SumVariance_DNA_10_01
Cytoplasm_Texture_SumVariance_DNA_10_02
Cytoplasm_Texture_SumVariance_DNA_10_03
Cytoplasm_Texture_SumVariance_DNA_20_00
Cytoplasm_Texture_SumVariance_DNA_20_01
Cytoplasm_Texture_SumVariance_DNA_20_02
Cytoplasm_Texture_SumVariance_DNA_20_03
Cytoplasm_Texture_SumVariance_DNA_5_00

Cytoplasm_Texture_SumVariance_DNA_5_01
Cytoplasm_Texture_SumVariance_DNA_5_02
Cytoplasm_Texture_SumVariance_DNA_5_03
Cytoplasm_Texture_SumVariance_ER_10_00
Cytoplasm_Texture_SumVariance_ER_10_01
Cytoplasm_Texture_SumVariance_ER_10_02
Cytoplasm_Texture_SumVariance_ER_10_03
Cytoplasm_Texture_SumVariance_ER_20_00
Cytoplasm_Texture_SumVariance_ER_20_01
Cytoplasm_Texture_SumVariance_ER_20_02
Cytoplasm_Texture_SumVariance_ER_20_03
Cytoplasm_Texture_SumVariance_ER_5_00
Cytoplasm_Texture_SumVariance_ER_5_01
Cytoplasm_Texture_SumVariance_ER_5_02
Cytoplasm_Texture_SumVariance_ER_5_03
Cytoplasm_Texture_SumVariance_Mito_10_00
Cytoplasm_Texture_SumVariance_Mito_10_01
Cytoplasm_Texture_SumVariance_Mito_10_02
Cytoplasm_Texture_SumVariance_Mito_10_03
Cytoplasm_Texture_SumVariance_Mito_20_00
Cytoplasm_Texture_SumVariance_Mito_20_01
Cytoplasm_Texture_SumVariance_Mito_20_02
Cytoplasm_Texture_SumVariance_Mito_20_03
Cytoplasm_Texture_SumVariance_Mito_5_00
Cytoplasm_Texture_SumVariance_Mito_5_01
Cytoplasm_Texture_SumVariance_Mito_5_02
Cytoplasm_Texture_SumVariance_Mito_5_03
Cytoplasm_Texture_SumVariance_RNA_10_00
Cytoplasm_Texture_SumVariance_RNA_10_01
Cytoplasm_Texture_SumVariance_RNA_10_02
Cytoplasm_Texture_SumVariance_RNA_10_03
Cytoplasm_Texture_SumVariance_RNA_20_00
Cytoplasm_Texture_SumVariance_RNA_20_01
Cytoplasm_Texture_SumVariance_RNA_20_02
Cytoplasm_Texture_SumVariance_RNA_20_03
Cytoplasm_Texture_SumVariance_RNA_5_00
Cytoplasm_Texture_SumVariance_RNA_5_01
Cytoplasm_Texture_SumVariance_RNA_5_02
Cytoplasm_Texture_SumVariance_RNA_5_03

Cytoplasm_Texture_Variance_AGP_10_00
Cytoplasm_Texture_Variance_AGP_10_01
Cytoplasm_Texture_Variance_AGP_10_02
Cytoplasm_Texture_Variance_AGP_10_03
Cytoplasm_Texture_Variance_AGP_20_00
Cytoplasm_Texture_Variance_AGP_20_01
Cytoplasm_Texture_Variance_AGP_20_02
Cytoplasm_Texture_Variance_AGP_20_03
Cytoplasm_Texture_Variance_AGP_5_00
Cytoplasm_Texture_Variance_AGP_5_01
Cytoplasm_Texture_Variance_AGP_5_02
Cytoplasm_Texture_Variance_AGP_5_03
Cytoplasm_Texture_Variance_Brightfield_10_00
Cytoplasm_Texture_Variance_Brightfield_10_01
Cytoplasm_Texture_Variance_Brightfield_10_02
Cytoplasm_Texture_Variance_Brightfield_10_03
Cytoplasm_Texture_Variance_Brightfield_20_00
Cytoplasm_Texture_Variance_Brightfield_20_01
Cytoplasm_Texture_Variance_Brightfield_20_02
Cytoplasm_Texture_Variance_Brightfield_20_03
Cytoplasm_Texture_Variance_Brightfield_5_00
Cytoplasm_Texture_Variance_Brightfield_5_01
Cytoplasm_Texture_Variance_Brightfield_5_02
Cytoplasm_Texture_Variance_Brightfield_5_03
Cytoplasm_Texture_Variance_DNA_10_00
Cytoplasm_Texture_Variance_DNA_10_01
Cytoplasm_Texture_Variance_DNA_10_02
Cytoplasm_Texture_Variance_DNA_10_03
Cytoplasm_Texture_Variance_DNA_20_00
Cytoplasm_Texture_Variance_DNA_20_01
Cytoplasm_Texture_Variance_DNA_20_02
Cytoplasm_Texture_Variance_DNA_20_03
Cytoplasm_Texture_Variance_DNA_5_00
Cytoplasm_Texture_Variance_DNA_5_01
Cytoplasm_Texture_Variance_DNA_5_02
Cytoplasm_Texture_Variance_DNA_5_03
Cytoplasm_Texture_Variance_ER_10_00
Cytoplasm_Texture_Variance_ER_10_01
Cytoplasm_Texture_Variance_ER_10_02

Cytoplasm_Texture_Variance_ER_10_03
Cytoplasm_Texture_Variance_ER_20_00
Cytoplasm_Texture_Variance_ER_20_01
Cytoplasm_Texture_Variance_ER_20_02
Cytoplasm_Texture_Variance_ER_20_03
Cytoplasm_Texture_Variance_ER_5_00
Cytoplasm_Texture_Variance_ER_5_01
Cytoplasm_Texture_Variance_ER_5_02
Cytoplasm_Texture_Variance_ER_5_03
Cytoplasm_Texture_Variance_Mito_10_00
Cytoplasm_Texture_Variance_Mito_10_01
Cytoplasm_Texture_Variance_Mito_10_02
Cytoplasm_Texture_Variance_Mito_10_03
Cytoplasm_Texture_Variance_Mito_20_00
Cytoplasm_Texture_Variance_Mito_20_01
Cytoplasm_Texture_Variance_Mito_20_02
Cytoplasm_Texture_Variance_Mito_20_03
Cytoplasm_Texture_Variance_Mito_5_00
Cytoplasm_Texture_Variance_Mito_5_01
Cytoplasm_Texture_Variance_Mito_5_02
Cytoplasm_Texture_Variance_Mito_5_03
Cytoplasm_Texture_Variance_RNA_10_00
Cytoplasm_Texture_Variance_RNA_10_01
Cytoplasm_Texture_Variance_RNA_10_02
Cytoplasm_Texture_Variance_RNA_10_03
Cytoplasm_Texture_Variance_RNA_20_00
Cytoplasm_Texture_Variance_RNA_20_01
Cytoplasm_Texture_Variance_RNA_20_02
Cytoplasm_Texture_Variance_RNA_20_03
Cytoplasm_Texture_Variance_RNA_5_00
Cytoplasm_Texture_Variance_RNA_5_01
Cytoplasm_Texture_Variance_RNA_5_02
Cytoplasm_Texture_Variance_RNA_5_03

Table S3. Composite traits for rare and common variant association tests.

Morphological traits which were selected for downstream association testing with rare and common variants. These features were chosen based on having a correlation of <0.9 with all other morphological traits contained in our dataset.

Trait
Cells_Texture_Contrast_RNA_5_02
Cells_Texture_Entropy_AGP_10_01
Cytoplasm_Texture_DifferenceVariance_DNA_5_00
Cells_Texture_SumEntropy_ER_5_01
Cytoplasm_Intensity_StdIntensityEdge_Mito
Cytoplasm_Texture_SumEntropy_Mito_10_00
Cytoplasm_Texture_SumEntropy_ER_5_00
Cells_Texture_InfoMeas1_ER_20_00
Nuclei_Texture_SumAverage_Brightfield_20_01
Nuclei_Texture_InfoMeas2_ER_5_00
Nuclei_Texture_InfoMeas2_Mito_20_01
Cells_Granularity_11_ER
Cells_Texture_AngularSecondMoment_ER_5_00
Cells_Texture_InfoMeas2_DNA_20_02
Nuclei_Texture_InfoMeas1_Brightfield_5_01
Cells_Texture_InfoMeas2_Mito_20_01
Cells_Intensity_LowerQuartileIntensity_RNA
Cells_RadialDistribution_FracAtD_Brightfield_3of4
Cells_Texture_InfoMeas2_RNA_5_03
Nuclei_Granularity_6_ER
Cytoplasm_RadialDistribution_FracAtD_ER_4of4
Cells_AreaShape_Area
Cells_Granularity_7_Brightfield
Cells_Intensity_LowerQuartileIntensity_ER
Cytoplasm_Intensity_MedianIntensity_Mito
Cells_Granularity_10_AGP
Cytoplasm_Texture_InverseDifferenceMoment_ER_10_00
Cells_Texture_InfoMeas1_Mito_5_01
Nuclei_RadialDistribution_FracAtD_AGP_4of4
Cells_Texture_InfoMeas2_Mito_5_01
Nuclei_AreaShape_Zernike_9_7
Nuclei_RadialDistribution_MeanFrac_DNA_4of4
Nuclei_Texture_InfoMeas1_AGP_20_00
Cytoplasm_Texture_AngularSecondMoment_ER_20_00
Cytoplasm_Texture_InfoMeas1_DNA_5_01
Cytoplasm_Texture_InfoMeas2_DNA_10_00
Cells_Granularity_9_Mito
Nuclei_Intensity_MedianIntensity_Mito

Cells_Intensity_MaxIntensityEdge_DNA
Cells_Granularity_5_AGP
Cells_RadialDistribution_MeanFrac_AGP_2of4
Nuclei_AreaShape_Zernike_0_0
Nuclei_AreaShape_Area
Cells_Granularity_4_ER
Cells_Texture_InfoMeas1_Mito_20_00
Cells_Texture_InfoMeas1_RNA_5_01
Nuclei_RadialDistribution_RadialCV_Mito_3of4
Cells_Texture_InfoMeas1_AGP_20_01
Nuclei_Intensity_StdIntensityEdge_DNA
Cells_RadialDistribution_FracAtD_RNA_4of4
Nuclei_Granularity_12_Mito
Cells_Texture_InfoMeas1_ER_5_00
Cells_RadialDistribution_RadialCV_Brightfield_3of4
Cells_Granularity_4_Brightfield
Cells_Granularity_5_RNA
Nuclei_Granularity_11_DNA
Cells_Texture_InfoMeas1_AGP_5_00
Cells_Intensity_IntegratedIntensity_Brightfield
Nuclei_RadialDistribution_MeanFrac_Mito_4of4
Cytoplasm_Intensity_MassDisplacement_RNA
Nuclei_Granularity_8_RNA
Nuclei_Texture_InfoMeas1_RNA_20_03
Cells_Granularity_6_Mito
Cytoplasm_Texture_InfoMeas2_RNA_5_00
Cells_AreaShape_Zernike_5_5
Cytoplasm_Texture_AngularSecondMoment_RNA_20_01
Nuclei_RadialDistribution_MeanFrac_RNA_2of4
Cells_Intensity_MinIntensity_AGP
Cytoplasm_RadialDistribution_FracAtD_DNA_4of4
Cytoplasm_RadialDistribution_MeanFrac_DNA_1of4
Nuclei_RadialDistribution_RadialCV_Brightfield_2of4
Nuclei_Texture_InfoMeas1_Mito_5_00
Cells_RadialDistribution_FracAtD_AGP_2of4
Nuclei_Intensity_MassDisplacement_AGP
Cells_AreaShape_Compactness
Cells_RadialDistribution_FracAtD_Mito_2of4
Cytoplasm_Intensity_IntegratedIntensityEdge_ER

Cells_Intensity_IntegratedIntensityEdge_AGP
Cytoplasm_AreaShape_Zernike_4_4
Cytoplasm_RadialDistribution_MeanFrac_Brightfield_4of4
Cytoplasm_Texture_DifferenceVariance_ER_20_03
Nuclei_RadialDistribution_FracAtD_ER_2of4
Nuclei_RadialDistribution_MeanFrac_ER_4of4
Nuclei_RadialDistribution_RadialCV_DNA_3of4
Nuclei_Intensity_MassDisplacement_RNA
Cells_Granularity_1_Brightfield
Cells_Granularity_13_Brightfield
Cells_Granularity_5_Mito
Cells_RadialDistribution_FracAtD_ER_4of4
Cells_RadialDistribution_RadialCV_DNA_2of4
Cytoplasm_Granularity_3_RNA
Cytoplasm_RadialDistribution_MeanFrac_Mito_2of4
Cytoplasm_RadialDistribution_MeanFrac_RNA_1of4
Cytoplasm_RadialDistribution_RadialCV_AGP_2of4
Cytoplasm_RadialDistribution_RadialCV_Mito_2of4
Cytoplasm_RadialDistribution_RadialCV_RNA_2of4
Cells_AreaShape_Zernike_7_5
Cells_Texture_InfoMeas1_DNA_5_00
Cytoplasm_Intensity_MassDisplacement_Mito
Nuclei_Granularity_3_RNA
Nuclei_Texture_InfoMeas1_RNA_5_00
Cells_AreaShape_MaximumRadius
Cells_Intensity_IntegratedIntensity_Mito
Cells_Texture_AngularSecondMoment_ER_20_03
Cytoplasm_Granularity_7_AGP
Cytoplasm_Intensity_MassDisplacement_ER
Cytoplasm_RadialDistribution_FracAtD_Mito_1of4
Cytoplasm_RadialDistribution_RadialCV_DNA_2of4
Cytoplasm_Texture_InfoMeas1_AGP_10_00
Nuclei_RadialDistribution_FracAtD_Brightfield_4of4
Cells_AreaShape_Zernike_6_6
Cells_Granularity_2_Mito
Cells_Granularity_8_AGP
Cells_Intensity_IntegratedIntensity_RNA
Cytoplasm_RadialDistribution_MeanFrac_ER_2of4
Nuclei_Intensity_MinIntensity_ER

Nuclei_RadialDistribution_FracAtD_Mito_1of4
Cytoplasm_Granularity_12_Brightfield
Cells_AreaShape_Eccentricity
Cells_AreaShape_Zernike_6_4
Cells_AreaShape_Zernike_8_6
Cells_Granularity_1_ER
Cells_Granularity_1_Mito
Cells_Granularity_2_AGP
Cells_Granularity_2_Brightfield
Cells_Granularity_2_ER
Cells_RadialDistribution_MeanFrac_Mito_1of4
Cells_RadialDistribution_RadialCV_AGP_1of4
Cells_RadialDistribution_RadialCV_ER_2of4
Cells_RadialDistribution_RadialCV_Mito_1of4
Cells_Texture_InfoMeas1_AGP_10_00
Cells_Texture_InfoMeas1_AGP_10_01
Cells_Texture_InfoMeas1_DNA_10_01
Cytoplasm_RadialDistribution_MeanFrac_AGP_1of4
Cytoplasm_Texture_InfoMeas1_AGP_5_01
Cytoplasm_Texture_InfoMeas1_DNA_10_01
Cytoplasm_Texture_InfoMeas1_DNA_20_00
Cytoplasm_Texture_InfoMeas1_RNA_5_01
Nuclei_Granularity_2_DNA
Nuclei_Intensity_IntegratedIntensity_Brightfield
Nuclei_Intensity_IntegratedIntensity_ER
Nuclei_RadialDistribution_FracAtD_RNA_3of4
Nuclei_RadialDistribution_RadialCV_ER_1of4
Nuclei_RadialDistribution_RadialCV_RNA_1of4
Nuclei_Texture_InfoMeas1_AGP_5_00
Cytoplasm_AreaShape_Extent
Nuclei_Granularity_13_AGP
Cells_Granularity_3_AGP
Cells_RadialDistribution_MeanFrac_DNA_4of4
Cells_RadialDistribution_RadialCV_ER_3of4
Cytoplasm_AreaShape_Zernike_3_3
Nuclei_Granularity_7_DNA
Nuclei_RadialDistribution_MeanFrac_Brightfield_3of4
Nuclei_RadialDistribution_RadialCV_AGP_1of4
Cells_AreaShape_MeanRadius

Cells_Granularity_2_RNA
Cells_Granularity_3_Mito
Cells_Granularity_4_AGP
Cells_Intensity_MaxIntensityEdge_ER
Cells_RadialDistribution_MeanFrac_Brightfield_1of4
Cells_RadialDistribution_RadialCV_AGP_4of4
Cells_RadialDistribution_RadialCV_ER_4of4
Cytoplasm_AreaShape_Zernike_6_0
Cytoplasm_RadialDistribution_RadialCV_Brightfield_2of4
Cytoplasm_Texture_InfoMeas1_AGP_10_03
Nuclei_AreaShape_Zernike_3_3
Cells_AreaShape_Zernike_1_1
Cells_AreaShape_Zernike_3_1
Cells_AreaShape_Zernike_4_0
Cells_AreaShape_Zernike_4_2
Cells_AreaShape_Zernike_5_1
Cells_AreaShape_Zernike_5_3
Cells_AreaShape_Zernike_6_0
Cells_AreaShape_Zernike_6_2
Cells_AreaShape_Zernike_7_1
Cells_AreaShape_Zernike_7_3
Cells_AreaShape_Zernike_8_0
Cells_AreaShape_Zernike_8_4
Cells_AreaShape_Zernike_9_1
Cells_Intensity_IntegratedIntensityEdge_Mito
Cells_Intensity_IntegratedIntensityEdge_RNA
Cells_Intensity_MassDisplacement_Brightfield
Cells_Intensity_MassDisplacement_DNA
Cells_RadialDistribution_FracAtD_DNA_3of4
Cells_RadialDistribution_FracAtD_Mito_3of4
Cells_RadialDistribution_MeanFrac_Brightfield_3of4
Cells_RadialDistribution_MeanFrac_Brightfield_4of4
Cells_RadialDistribution_MeanFrac_ER_3of4
Cells_RadialDistribution_MeanFrac_ER_4of4
Cells_RadialDistribution_RadialCV_AGP_3of4
Cells_RadialDistribution_RadialCV_DNA_4of4
Cells_RadialDistribution_RadialCV_RNA_1of4
Cytoplasm_AreaShape_Compactness
Cytoplasm_AreaShape_Zernike_1_1

Cytoplasm_AreaShape_Zernike_2_0
Cytoplasm_AreaShape_Zernike_4_2
Cytoplasm_AreaShape_Zernike_5_3
Cytoplasm_AreaShape_Zernike_6_2
Cytoplasm_AreaShape_Zernike_7_3
Cytoplasm_AreaShape_Zernike_8_0
Cytoplasm_AreaShape_Zernike_8_2
Cytoplasm_AreaShape_Zernike_8_4
Cytoplasm_AreaShape_Zernike_9_1
Cytoplasm_AreaShape_Zernike_9_3
Cytoplasm_AreaShape_Zernike_9_5
Cytoplasm_Granularity_1_RNA
Cytoplasm_Granularity_3_AGP
Cytoplasm_Granularity_3_Brightfield
Cytoplasm_Granularity_4_Mito
Cytoplasm_Intensity_IntegratedIntensity_DNA
Cytoplasm_Intensity_MassDisplacement_Brightfield
Cytoplasm_Intensity_MassDisplacement_DNA
Cytoplasm_RadialDistribution_FracAtD_AGP_1of4
Cytoplasm_RadialDistribution_MeanFrac_AGP_3of4
Cytoplasm_RadialDistribution_MeanFrac_AGP_4of4
Cytoplasm_RadialDistribution_MeanFrac_ER_4of4
Cytoplasm_RadialDistribution_MeanFrac_Mito_4of4
Cytoplasm_RadialDistribution_MeanFrac_RNA_4of4
Cytoplasm_RadialDistribution_RadialCV_Brightfield_1of4
Cytoplasm_RadialDistribution_RadialCV_DNA_4of4
Cytoplasm_RadialDistribution_RadialCV_ER_1of4
Cytoplasm_RadialDistribution_RadialCV_ER_2of4
Cytoplasm_RadialDistribution_RadialCV_ER_3of4
Nuclei_AreaShape_Zernike_2_2
Nuclei_AreaShape_Zernike_4_2
Nuclei_AreaShape_Zernike_6_0
Nuclei_AreaShape_Zernike_6_2
Nuclei_AreaShape_Zernike_6_4
Nuclei_AreaShape_Zernike_8_0
Nuclei_AreaShape_Zernike_8_2
Nuclei_AreaShape_Zernike_8_6
Nuclei_AreaShape_Zernike_9_1
Nuclei_Granularity_1_DNA

Nuclei_Granularity_1_ER
Nuclei_Granularity_1_Mito
Nuclei_Granularity_2_Brightfield
Nuclei_Granularity_2_ER
Nuclei_Granularity_6_DNA
Nuclei_Granularity_7_Mito
Nuclei_Granularity_9_AGP
Nuclei_RadialDistribution_FracAtD_Mito_3of4
Nuclei_RadialDistribution_FracAtD_RNA_1of4
Nuclei_RadialDistribution_MeanFrac_Brightfield_1of4
Nuclei_RadialDistribution_MeanFrac_Brightfield_2of4
Nuclei_RadialDistribution_MeanFrac_ER_1of4
Nuclei_RadialDistribution_RadialCV_DNA_1of4

Table S4. Morphology traits with nominal association to rare variants in *WASF2*.

List of morphological traits which have nominal significance with association to rare variant burden in *WASF2*. We provide the effect size (β estimate) and raw p-value of the association for each trait

TRAIT	GENE	B EST	P	CELLS	TRAIT CATEGORY
Cells_AreaShape_Compactness	WASF2	0.6017736	0.0178128	colony	AreaShape
Cells_RadialDistribution_FracAtD_Mito_2of4	WASF2	-1.1361109	0.0001998	colony	RadialDistribution
Cells_Intensity_IntegratedIntensityEdge_AGP	WASF2	0.5112278	0.000634	colony	Intensity
Cytoplasm_AreaShape_Zernike_4_4	WASF2	-0.333889	0.0298563	colony	AreaShape
Nuclei_RadialDistribution_FracAtD_ER_2of4	WASF2	-0.5671898	0.0042808	colony	RadialDistribution
Nuclei_RadialDistribution_MeanFrac_ER_4of4	WASF2	-0.6604801	0.0005766	colony	RadialDistribution
Nuclei_RadialDistribution_RadialCV_DNA_3of4	WASF2	0.4122793	0.0026387	colony	RadialDistribution
Cells_RadialDistribution_FracAtD_ER_4of4	WASF2	0.8142418	0.0006957	colony	RadialDistribution
Cells_RadialDistribution_RadialCV_DNA_2of4	WASF2	-0.590796	0.0010327	colony	RadialDistribution
Cytoplasm_RadialDistribution_RadialCV_Mito_2of4	WASF2	-0.6062079	0.0067084	colony	RadialDistribution
Cells_AreaShape_MaximumRadius	WASF2	-0.5603363	0.0314627	colony	AreaShape
Cells_Texture_AngularSecondMoment_ER_20_03	WASF2	0.4638048	0.0352444	colony	Texture
Cytoplasm_Granularity_7_AGP	WASF2	-0.37654	0.002002	colony	Granularity
Cytoplasm_Intensity_MassDisplacement_ER	WASF2	0.4027906	0.0281393	colony	Intensity
Nuclei_RadialDistribution_FracAtD_Brightfield_4of4	WASF2	1.0981268	0.0002245	colony	RadialDistribution
Cells_Granularity_8_AGP	WASF2	-0.4047754	0.0130437	colony	Granularity
Cells_AreaShape_Eccentricity	WASF2	0.5963399	0.0338874	colony	AreaShape
Cells_Granularity_2_AGP	WASF2	0.3518073	0.0461333	colony	Granularity
Cells_RadialDistribution_RadialCV_Mito_1of4	WASF2	-0.3821848	0.0341008	colony	RadialDistribution

Cells_Texture_InfoMeas1_DNA_10_01	WASF2	-0.6511002	0.0001529	colony	Texture
Cytoplasm_Texture_InfoMeas1_DNA_10_01	WASF2	-0.7078816	0.0004732	colony	Texture
Cytoplasm_Texture_InfoMeas1_DNA_20_00	WASF2	-0.8277485	8.374E-05	colony	Texture
Nuclei_RadialDistribution_RadialCV_RNA_1of4	WASF2	-0.29101	0.0155395	colony	RadialDistribution
Cytoplasm_AreaShape_Extent	WASF2	-0.8858641	0.0013642	colony	AreaShape
Nuclei_Granularity_13_AGP	WASF2	0.3997386	0.0408859	colony	Granularity
Cells_RadialDistribution_MeanFrac_DNA_4of4	WASF2	0.9318605	3.228E-06	colony	RadialDistribution
Cytoplasm_AreaShape_Zernike_3_3	WASF2	-0.7213938	0.0072769	colony	AreaShape
Nuclei_RadialDistribution_MeanFrac_Brightfield_3of4	WASF2	-0.8479671	6.28E-05	colony	RadialDistribution
Cells_AreaShape_MeanRadius	WASF2	-0.6516643	0.0197043	colony	AreaShape
Cells_Granularity_2_RNA	WASF2	0.3708179	0.013173	colony	Granularity
Cells_Granularity_11_ER	WASF2	-0.2429185	0.0458579	colony	Granularity
Nuclei_Texture_InfoMeas1_Brightfield_5_01	WASF2	-0.3401803	0.0083151	colony	Texture
Cells_Intensity_MaxIntensityEdge_ER	WASF2	0.4764551	0.027194	colony	Intensity
Cells_RadialDistribution_RadialCV_AGP_4of4	WASF2	0.4564188	0.0024917	colony	RadialDistribution
Cytoplasm_AreaShape_Zernike_6_0	WASF2	-0.9662585	0.0003932	colony	AreaShape
Nuclei_AreaShape_Zernike_3_3	WASF2	0.5817608	0.0105419	colony	AreaShape
Cells_AreaShape_Zernike_1_1	WASF2	-0.7532907	0.0077673	colony	AreaShape
Cells_AreaShape_Zernike_6_2	WASF2	0.4835634	0.0425625	colony	AreaShape
Cells_AreaShape_Zernike_8_0	WASF2	0.3280529	0.0499159	colony	AreaShape
Cells_Intensity_IntegratedIntensityEdge_Mito	WASF2	0.8036502	2.573E-05	colony	Intensity
Cells_Intensity_IntegratedIntensityEdge_RNA	WASF2	0.5452743	0.0041773	colony	Intensity
Cells_Intensity_MassDisplacement_Brightfield	WASF2	0.4846785	0.00053	colony	Intensity
Cells_Intensity_MassDisplacement_DNA	WASF2	0.5218184	0.0053568	colony	Intensity
Cells_RadialDistribution_FracAtD_DNA_3of4	WASF2	-0.44752	0.0125261	colony	RadialDistribution
Cells_RadialDistribution_FracAtD_Mito_3of4	WASF2	-1.1337213	2.181E-05	colony	RadialDistribution
Cells_RadialDistribution_MeanFrac_ER_4of4	WASF2	0.7398511	0.000422	colony	RadialDistribution
Cells_RadialDistribution_RadialCV_DNA_4of4	WASF2	0.8813744	5.218E-07	colony	RadialDistribution
Cytoplasm_AreaShape_Compactness	WASF2	0.882947	0.0018131	colony	AreaShape
Cytoplasm_AreaShape_Zernike_1_1	WASF2	-0.8070865	0.0033056	colony	AreaShape
Cytoplasm_AreaShape_Zernike_2_0	WASF2	-0.5165041	0.0106019	colony	AreaShape
Cytoplasm_AreaShape_Zernike_6_2	WASF2	-0.6693354	0.0112057	colony	AreaShape
Cytoplasm_AreaShape_Zernike_7_3	WASF2	-0.7699828	0.0010112	colony	AreaShape
Cytoplasm_AreaShape_Zernike_8_0	WASF2	-1.3983755	1.767E-07	colony	AreaShape
Cytoplasm_AreaShape_Zernike_8_2	WASF2	-1.3039356	1.239E-06	colony	AreaShape
Cytoplasm_AreaShape_Zernike_9_1	WASF2	-1.3619497	3.784E-08	colony	AreaShape
Cytoplasm_AreaShape_Zernike_9_3	WASF2	-1.2406166	3.038E-10	colony	AreaShape
Cytoplasm_Granularity_4_Mito	WASF2	-0.6410883	0.0084336	colony	Granularity
Cytoplasm_Intensity_MassDisplacement_Brightfield	WASF2	0.5457276	0.0009247	colony	Intensity

Cytoplasm_Intensity_MassDisplacement_DNA	WASF2	0.6338015	0.0016026	colony	Intensity
Cytoplasm_RadialDistribution_RadialCV_DNA_4of4	WASF2	0.4360133	0.0116073	colony	RadialDistribution
Nuclei_AreaShape_Zernike_2_2	WASF2	0.7143277	0.0152502	colony	AreaShape
Nuclei_AreaShape_Zernike_4_2	WASF2	1.0127704	0.0003	colony	AreaShape
Nuclei_AreaShape_Zernike_6_0	WASF2	-0.8407323	0.0006146	colony	AreaShape
Nuclei_AreaShape_Zernike_6_4	WASF2	-0.6124806	0.0040547	colony	AreaShape
Nuclei_AreaShape_Zernike_8_2	WASF2	-0.8280235	5.816E-05	colony	AreaShape
Nuclei_AreaShape_Zernike_8_6	WASF2	0.5680858	0.0413446	colony	AreaShape
Nuclei_Granularity_2_Brightfield	WASF2	0.3839519	0.0292795	colony	Granularity
Cells_RadialDistribution_FracAtD_Brightfield_3of4	WASF2	-0.5075018	0.0220096	colony	RadialDistribution
Nuclei_Granularity_6_DNA	WASF2	-0.7543435	0.0009715	colony	Granularity
Nuclei_Granularity_7_Mito	WASF2	-0.4279102	0.0434397	colony	Granularity
Nuclei_RadialDistribution_FracAtD_RNA_1of4	WASF2	-0.5649847	0.0009358	colony	RadialDistribution
Nuclei_RadialDistribution_MeanFrac_Brightfield_2of4	WASF2	0.2859392	0.0362378	colony	RadialDistribution
Nuclei_RadialDistribution_FracAtD_AGP_4of4	WASF2	0.4700853	0.0014464	colony	RadialDistribution
Nuclei_AreaShape_Zernike_9_7	WASF2	0.8159485	0.0005127	colony	AreaShape
Cells_Granularity_9_Mito	WASF2	-0.3632483	0.0284396	colony	Granularity
Cells_Intensity_MaxIntensityEdge_DNA	WASF2	0.6249189	0.0002926	colony	Intensity
Cells_RadialDistribution_MeanFrac_AGP_2of4	WASF2	-0.4731299	0.0003625	colony	RadialDistribution
Nuclei_AreaShape_Zernike_0_0	WASF2	-1.0756328	0.0001133	colony	AreaShape
Cells_Texture_InfoMeas1_Mito_20_00	WASF2	-0.593503	0.0069815	colony	Texture
Cells_Texture_InfoMeas1_AGP_20_01	WASF2	-0.5692211	0.0047877	colony	Texture
Nuclei_Intensity_StdIntensityEdge_DNA	WASF2	0.3780176	0.0004836	colony	Intensity
Cells_RadialDistribution_FracAtD_RNA_4of4	WASF2	0.9327524	9.39E-05	colony	RadialDistribution
Cells_Granularity_4_Brightfield	WASF2	0.4013453	0.0464732	colony	Granularity
Nuclei_Granularity_11_DNA	WASF2	0.5069649	0.0059807	colony	Granularity
Nuclei_RadialDistribution_MeanFrac_Mito_4of4	WASF2	-0.8906262	6.452E-07	colony	RadialDistribution
Cytoplasm_Intensity_MassDisplacement_RNA	WASF2	0.3430615	0.0403983	colony	Intensity
Nuclei_Granularity_8_RNA	WASF2	-0.350383	0.0161217	colony	Granularity
Cytoplasm_Texture_AngularSecondMoment_RNA_20_01	WASF2	0.4478666	0.0032128	colony	Texture
Cytoplasm_RadialDistribution_MeanFrac_DNA_1of4	WASF2	-0.6180675	0.0087686	colony	RadialDistribution
Cells_RadialDistribution_FracAtD_AGP_2of4	WASF2	-0.9079653	4.65E-06	colony	RadialDistribution

Table S5. Morphology traits with nominal association to rare variants in *PRLR*.

List of morphological traits which have nominal significance with association to rare variant burden in *PRLR*. We provide the effect size (β estimate) and raw p-value of the association for each trait.

TRAIT	GENE	B EST	P	CELLS	TRAIT CATEGORY
Cells_Texture_Entropy_AGP_10_01	PRLR	-0.3557241	0.0034529	isolate	Texture
Cytoplasm_Texture_DifferenceVariance_DNA_5_00	PRLR	-0.4561351	0.0005869	isolate	Texture
Cytoplasm_Intensity_StdIntensityEdge_Mito	PRLR	-0.8470681	0.0001267	isolate	Intensity
Cytoplasm_Texture_SumEntropy_ER_5_00	PRLR	0.5041965	0.004862	isolate	Texture
Cells_AreaShape_Compactness	PRLR	-0.8966286	0.0019508	isolate	AreaShape
Cells_RadialDistribution_FracAtD_Mito_2of4	PRLR	-0.6374274	0.0087116	isolate	RadialDistribution
Nuclei_Intensity_MassDisplacement_RNA	PRLR	-0.7257857	8.414E-05	isolate	Intensity
Cells_Granularity_1_Brightfield	PRLR	-0.9904826	0.0009598	isolate	Granularity
Cells_Granularity_13_Brightfield	PRLR	0.3650933	0.0380143	isolate	Granularity
Cells_Granularity_5_Mito	PRLR	-0.6065456	0.0115139	isolate	Granularity
Cells_RadialDistribution_RadialCV_DNA_2of4	PRLR	-0.8313656	1.644E-06	isolate	RadialDistribution
Cytoplasm_Granularity_3_RNA	PRLR	0.4132344	0.0119737	isolate	Granularity
Cytoplasm_RadialDistribution_MeanFrac_RNA_1of4	PRLR	-0.6249384	0.0118366	isolate	RadialDistribution
Cytoplasm_RadialDistribution_RadialCV_Mito_2of4	PRLR	-1.2108234	7.224E-07	isolate	RadialDistribution
Cytoplasm_RadialDistribution_RadialCV_RNA_2of4	PRLR	-0.9052167	3.755E-05	isolate	RadialDistribution
Cells_Texture_InfoMeas1_DNA_5_00	PRLR	0.9280761	1.589E-06	isolate	Texture
Cytoplasm_Intensity_MassDisplacement_Mito	PRLR	-0.8238812	0.0003447	isolate	Intensity
Nuclei_Texture_InfoMeas1_RNA_5_00	PRLR	0.4187454	0.0202438	isolate	Texture
Cells_AreaShape_MaximumRadius	PRLR	-0.6664863	0.0051358	isolate	AreaShape
Cells_Intensity_IntegratedIntensity_Mito	PRLR	-0.7216642	0.003493	isolate	Intensity
Cytoplasm_Granularity_7_AGP	PRLR	-0.3666169	0.0418282	isolate	Granularity
Cytoplasm_RadialDistribution_RadialCV_DNA_2of4	PRLR	-0.5547316	0.0052375	isolate	RadialDistribution
Cells_AreaShape_Zernike_6_6	PRLR	0.4183531	0.0238654	isolate	AreaShape
Cells_Granularity_2_Mito	PRLR	-1.046951	5.708E-05	isolate	Granularity
Cells_Granularity_8_AGP	PRLR	-0.6644403	0.004129	isolate	Granularity
Cells_Intensity_IntegratedIntensity_RNA	PRLR	-0.385728	0.0478212	isolate	Intensity
Nuclei_RadialDistribution_FracAtD_Mito_1of4	PRLR	0.80941	0.0006762	isolate	RadialDistribution
Cytoplasm_Granularity_12_Brightfield	PRLR	0.4022378	0.0183212	isolate	Granularity
Cells_AreaShape_Eccentricity	PRLR	-0.5982169	0.0237323	isolate	AreaShape
Cells_AreaShape_Zernike_6_4	PRLR	-0.5391383	0.0164723	isolate	AreaShape
Cells_AreaShape_Zernike_8_6	PRLR	-0.7855149	9.717E-05	isolate	AreaShape
Cells_Granularity_1_Mito	PRLR	0.7692888	0.0004519	isolate	Granularity
Cells_Granularity_2_Brightfield	PRLR	-0.5058511	0.0201259	isolate	Granularity
Cells_RadialDistribution_MeanFrac_Mito_1of4	PRLR	-0.9047417	3.41E-06	isolate	RadialDistribution
Cells_RadialDistribution_RadialCV_AGP_1of4	PRLR	-0.7015553	0.0004939	isolate	RadialDistribution
Cells_RadialDistribution_RadialCV_ER_2of4	PRLR	-0.4487259	0.0312959	isolate	RadialDistribution
Cells_RadialDistribution_RadialCV_Mito_1of4	PRLR	-1.1866286	1.263E-08	isolate	RadialDistribution
Cells_Texture_InfoMeas1_AGP_10_00	PRLR	1.0202095	0.0001267	isolate	Texture

Cells_Texture_InfoMeas1_AGP_10_01	PRLR	0.8362158	0.0012202	isolate	Texture
Cytoplasm_Texture_InfoMeas1_DNA_10_01	PRLR	-0.7082782	0.000101	isolate	Texture
Cytoplasm_Texture_InfoMeas1_DNA_20_00	PRLR	-0.891027	1.049E-08	isolate	Texture
Nuclei_Granularity_13_AGP	PRLR	-0.7233574	0.0028743	isolate	Granularity
Cells_RadialDistribution_MeanFrac_DNA_4of4	PRLR	0.8131307	1.246E-05	isolate	RadialDistribution
Cells_AreaShape_MeanRadius	PRLR	-0.6133381	0.0211087	isolate	AreaShape
Cells_Granularity_2_RNA	PRLR	0.3161193	0.0316986	isolate	Granularity
Nuclei_Texture_InfoMeas2_ER_5_00	PRLR	-0.4550886	0.0233363	isolate	Texture
Nuclei_Texture_InfoMeas2_Mito_20_01	PRLR	-0.6243651	0.0069638	isolate	Texture
Cells_Granularity_11_ER	PRLR	-0.7339797	0.0059197	isolate	Granularity
Cells_Intensity_LowerQuartileIntensity_RNA	PRLR	0.6303853	0.0003323	isolate	Intensity
Cells_Granularity_3_Mito	PRLR	-0.6937979	0.0035592	isolate	Granularity
Cells_Intensity_MaxIntensityEdge_ER	PRLR	0.9404135	9.324E-07	isolate	Intensity
Cells_RadialDistribution_MeanFrac_Brightfield_1of4	PRLR	0.4316748	0.0108063	isolate	RadialDistribution
Cytoplasm_Texture_InfoMeas1_AGP_10_03	PRLR	-0.3493902	0.0150413	isolate	Texture
Cells_AreaShape_Zernike_3_1	PRLR	-0.8572486	0.0003496	isolate	AreaShape
Cells_AreaShape_Zernike_5_1	PRLR	-0.3930979	0.0369342	isolate	AreaShape
Cells_AreaShape_Zernike_5_3	PRLR	-0.7687688	0.0001674	isolate	AreaShape
Cells_AreaShape_Zernike_7_1	PRLR	-0.5140015	0.0008748	isolate	AreaShape
Cells_AreaShape_Zernike_9_1	PRLR	-0.3741291	0.0132904	isolate	AreaShape
Cells_Intensity_IntegratedIntensityEdge_Mito	PRLR	0.4268803	0.031123	isolate	Intensity
Cells_Intensity_MassDisplacement_DNA	PRLR	-0.8463041	2.968E-05	isolate	Intensity
Cells_RadialDistribution_MeanFrac_Brightfield_3of4	PRLR	0.490192	0.0214856	isolate	RadialDistribution
Cells_RadialDistribution_MeanFrac_Brightfield_4of4	PRLR	-0.423764	0.0235673	isolate	RadialDistribution
Cells_RadialDistribution_MeanFrac_ER_3of4	PRLR	-0.7981181	0.0010624	isolate	RadialDistribution
Cells_RadialDistribution_MeanFrac_ER_4of4	PRLR	0.9067177	1.512E-06	isolate	RadialDistribution
Cells_RadialDistribution_RadialCV_AGP_3of4	PRLR	-0.8982881	1.12E-05	isolate	RadialDistribution
Cells_RadialDistribution_RadialCV_RNA_1of4	PRLR	-0.3519557	0.0222467	isolate	RadialDistribution
Cytoplasm_AreaShape_Zernike_1_1	PRLR	0.52193	0.023639	isolate	AreaShape
Cytoplasm_AreaShape_Zernike_2_0	PRLR	-0.7640335	1.188E-05	isolate	AreaShape
Cytoplasm_Granularity_1_RNA	PRLR	0.4176973	0.0115372	isolate	Granularity
Cytoplasm_Granularity_4_Mito	PRLR	-0.4985007	0.049474	isolate	Granularity
Cytoplasm_RadialDistribution_FracAtD_AGP_1of4	PRLR	0.5921326	0.0109885	isolate	RadialDistribution
Cytoplasm_RadialDistribution_RadialCV_ER_1of4	PRLR	-0.7378536	0.0007993	isolate	RadialDistribution
Cytoplasm_RadialDistribution_RadialCV_ER_2of4	PRLR	-0.8352595	0.0004154	isolate	RadialDistribution
Cytoplasm_RadialDistribution_RadialCV_ER_3of4	PRLR	-0.8864676	2.823E-05	isolate	RadialDistribution
Nuclei_AreaShape_Zernike_9_1	PRLR	0.3591145	0.0210986	isolate	AreaShape
Nuclei_Granularity_1_DNA	PRLR	0.8505475	2.117E-06	isolate	Granularity
Nuclei_Granularity_1_ER	PRLR	0.7179083	0.001736	isolate	Granularity

Nuclei_Granularity_1_Mito	PRLR	0.8230442	0.0006055	isolate	Granularity
Nuclei_Granularity_2_Brightfield	PRLR	-0.594136	0.006281	isolate	Granularity
Cells_RadialDistribution_FracAtD_Brightfield_3of4	PRLR	0.7217559	0.0025756	isolate	RadialDistribution
Cells_Texture_InfoMeas2_RNA_5_03	PRLR	-0.8139515	4.33E-05	isolate	Texture
Nuclei_Granularity_6_ER	PRLR	-0.8300917	4.724E-05	isolate	Granularity
Cytoplasm_RadialDistribution_FracAtD_ER_4of4	PRLR	-0.539062	0.0145527	isolate	RadialDistribution
Cells_AreaShape_Area	PRLR	-0.8934032	0.0001925	isolate	AreaShape
Cells_Intensity_LowerQuartileIntensity_ER	PRLR	0.8004329	2.834E-06	isolate	Intensity
Cytoplasm_Intensity_MedianIntensity_Mito	PRLR	0.7061762	3.343E-06	isolate	Intensity
Nuclei_Granularity_7_Mito	PRLR	-0.695204	0.0060171	isolate	Granularity
Nuclei_Granularity_9_AGP	PRLR	-0.7301686	0.0031766	isolate	Granularity
Nuclei_RadialDistribution_FracAtD_Mito_3of4	PRLR	0.4952221	0.0296223	isolate	RadialDistribution
Cells_Granularity_10_AGP	PRLR	-0.8181612	0.0008525	isolate	Granularity
Cytoplasm_Texture_InverseDifferenceMoment_ER_10_00	PRLR	-0.8540796	1.695E-05	isolate	Texture
Cells_Texture_InfoMeas1_Mito_5_01	PRLR	0.524889	0.0149343	isolate	Texture
Nuclei_RadialDistribution_FracAtD_AGP_4of4	PRLR	0.3743025	0.0048969	isolate	RadialDistribution
Cells_Texture_InfoMeas2_Mito_5_01	PRLR	-0.7402871	0.0022057	isolate	Texture
Nuclei_RadialDistribution_MeanFrac_DNA_4of4	PRLR	0.5921973	0.000399	isolate	RadialDistribution
Nuclei_Texture_InfoMeas1_AGP_20_00	PRLR	0.462526	0.0016304	isolate	Texture
Cytoplasm_Texture_InfoMeas2_DNA_10_00	PRLR	0.3767106	0.0169462	isolate	Texture
Cells_Granularity_9_Mito	PRLR	-0.858481	0.0019832	isolate	Granularity
Cells_Intensity_MaxIntensityEdge_DNA	PRLR	0.9881446	5.72E-10	isolate	Intensity
Cells_Granularity_5_AGP	PRLR	-0.3739489	0.0207113	isolate	Granularity
Cells_RadialDistribution_MeanFrac_AGP_2of4	PRLR	-0.7423354	1.069E-06	isolate	RadialDistribution
Cells_Texture_InfoMeas1_RNA_5_01	PRLR	0.7754414	7.574E-06	isolate	Texture
Nuclei_RadialDistribution_RadialCV_Mito_3of4	PRLR	-0.9262482	3.175E-05	isolate	RadialDistribution
Cells_Texture_InfoMeas1_AGP_20_01	PRLR	-0.3642686	0.005113	isolate	Texture
Nuclei_Granularity_12_Mito	PRLR	-0.915868	0.0004673	isolate	Granularity
Nuclei_Granularity_11_DNA	PRLR	-0.8237997	0.0007059	isolate	Granularity
Cells_Texture_InfoMeas1_AGP_5_00	PRLR	0.6169982	0.0002587	isolate	Texture
Cells_Intensity_IntegratedIntensity_Brightfield	PRLR	-0.6215621	0.012027	isolate	Intensity
Nuclei_RadialDistribution_MeanFrac_Mito_4of4	PRLR	-0.8316906	0.000488	isolate	RadialDistribution
Cytoplasm_Intensity_MassDisplacement_RNA	PRLR	-0.8851268	0.000254	isolate	Intensity
Nuclei_Granularity_8_RNA	PRLR	-0.9276078	0.0001638	isolate	Granularity
Cells_Granularity_6_Mito	PRLR	-0.6335881	0.0094296	isolate	Granularity
Cytoplasm_Texture_InfoMeas2_RNA_5_00	PRLR	-0.4514653	0.0111143	isolate	Texture
Cytoplasm_Texture_AngularSecondMoment_RNA_20_01	PRLR	0.4945651	0.0006634	isolate	Texture
Cells_Intensity_MinIntensity_AGP	PRLR	0.7291141	2.61E-05	isolate	Intensity
Cytoplasm_RadialDistribution_MeanFrac_DNA_1of4	PRLR	-0.9620107	0.0001228	isolate	RadialDistribution

Cells_RadialDistribution_FracAtD_AGP_2of4	PRLR	-0.4483205	0.0239086	isolate	RadialDistribution
Nuclei_Intensity_MassDisplacement_AGP	PRLR	-0.4448341	0.0101645	isolate	Intensity

Table S6. Morphology traits with nominal association to rare variants.

List of all morphological traits which showed nominal association to rare variants in our study. We provide the effect size (β estimate) and raw p-value of the association for each trait.

TRAIT	GENE	B EST	P	CELLS	TRAIT CATEGORY
Cytoplasm_Texture_DifferenceVariance_DNA_5_00	ZNF576	-0.5646098	2.734E-07	colony	Texture
Cytoplasm_Granularity_3_RNA	TSPAN15	0.8973459	3.711E-07	colony	Granularity
Cells_Texture_InfoMeas1_DNA_5_00	ZNF576	0.6963202	5.831E-07	colony	Texture
Nuclei_Texture_InfoMeas1_RNA_5_00	IFT46	0.5626494	2.498E-07	colony	Texture
Cells_RadialDistribution_RadialCV_DNA_4of4	WASF2	0.8813744	5.218E-07	colony	RadialDistribution
Cytoplasm_AreaShape_Zernike_8_0	WASF2	-1.3983755	1.767E-07	colony	AreaShape
Cytoplasm_AreaShape_Zernike_9_1	SCGB1D1	1.0421833	3.417E-07	colony	AreaShape
Cytoplasm_AreaShape_Zernike_9_1	WASF2	-1.3619497	3.784E-08	colony	AreaShape
Cytoplasm_AreaShape_Zernike_9_3	WASF2	-1.2406166	3.038E-10	colony	AreaShape
Cells_Intensity_LowerQuartileIntensity_ER	DGCR14	0.7537229	9.538E-07	colony	Intensity
Cytoplasm_Texture_InverseDifferenceMoment_ER_10_00	ZNF576	-0.8038411	5.952E-07	colony	Texture
Nuclei_RadialDistribution_MeanFrac_Mito_4of4	WASF2	-0.8906262	6.452E-07	colony	RadialDistribution
Cytoplasm_RadialDistribution_RadialCV_Mito_2of4	PRLR	-1.2108234	7.224E-07	isolate	RadialDistribution
Cells_AreaShape_Zernike_7_5	OR1C1	-0.7917319	7.776E-07	isolate	AreaShape
Cytoplasm_Granularity_7_AGP	PLAU	0.6104252	7.491E-07	isolate	Granularity
Cells_AreaShape_Zernike_6_4	OR1C1	-0.9409091	2.258E-07	isolate	AreaShape
Cells_AreaShape_Zernike_6_4	WIPI2	-1.0822189	3.497E-07	isolate	AreaShape
Cells_AreaShape_Zernike_8_6	OR1C1	-0.8464665	2.598E-07	isolate	AreaShape
Cells_RadialDistribution_RadialCV_Mito_1of4	PRLR	-1.1866286	1.263E-08	isolate	RadialDistribution
Cytoplasm_Texture_InfoMeas1_DNA_20_00	PRLR	-0.891027	1.049E-08	isolate	Texture
Cells_Intensity_MaxIntensityEdge_ER	PRLR	0.9404135	9.324E-07	isolate	Intensity
Cytoplasm_Granularity_3_AGP	AFF1	-0.466506	5.95E-07	isolate	Granularity
Cytoplasm_RadialDistribution_FracAtD_ER_4of4	ZNF436	-1.1155899	9.45E-07	isolate	RadialDistribution
Cytoplasm_Texture_InfoMeas2_DNA_10_00	ZBTB7C	0.6690934	4.385E-07	isolate	Texture
Cells_Intensity_MaxIntensityEdge_DNA	PRLR	0.9881446	5.72E-10	isolate	Intensity

Table S7. Oligo sequences for sgRNAs used in CRISPRi experiments.

Sequences for sgRNAs used in the CRISPRi validation experiments.

sgRNA name	Forward sequence	Reverse sequence
WASF2_a	CGTAATGGCGGACACAGGCA	TGCCTGTGTCCGCCATTACG

WASF2_b	GCGTAATGGCGGACACAGGC	GCCTGTGTCCGCCATTACGC
PRLR_a	GAAAGCCCAGCCCAGAAAAC	GTTTTCTGGGCTGGGCTTTC
PRLR_d	GGCTGGGGACCGGGGAGCGG	CCGCTCCCCGGTCCCCAGCC
TSPAN15_a	CCCGCCCCCTCCCAGACGCT	AGCGTCTGGGAGGGGGCGGG
TSPAN15_b	GCGGCTGCCTAGCGTCTGGG	CCCAGACGCTAGGCAGCCGC

Table S8. Morphology traits with suggestive evidence of association to common variants.

List of all morphological traits which show nominal association to common variants in our study. We provide the effect size (β estimate) and Bonferroni corrected p-value of the association for each trait.

TRAIT	CHR	SNP	POS	A1	A2	AF1	BETA	SE	P	Cell	TRAIT CATEGORY
Cells_Intensity_LowerQuartileIntensity_RNA	1	rs34961969	17223325	A	T	0.07	0.28	0.05	8.34E-09	intcol	Intensity
Cells_Intensity_IntegratedIntensity_Mito	1	rs71806205	29149682	A	AG	0.06	0.49	0.09	3.90E-08	intcol	Intensity
Nuclei_RadialDistribution_RadialCV_Brightfield_2of4	1	rs2943677	84584041	T	C	0.41	-0.19	0.03	3.17E-08	isolate	RadialDistribution
Nuclei_RadialDistribution_RadialCV_Brightfield_2of4	1	rs2943676	84584891	C	T	0.41	-0.19	0.03	3.17E-08	isolate	RadialDistribution
Nuclei_RadialDistribution_RadialCV_Brightfield_2of4	1	rs2943675	84585049	G	A	0.41	-0.19	0.03	3.17E-08	isolate	RadialDistribution
Nuclei_RadialDistribution_RadialCV_Brightfield_2of4	1	rs2945160	84585185	C	G	0.41	-0.19	0.03	3.17E-08	isolate	RadialDistribution
Nuclei_RadialDistribution_RadialCV_Brightfield_2of4	1	rs2994947	84586909	C	T	0.41	-0.19	0.03	3.66E-08	isolate	RadialDistribution
Nuclei_RadialDistribution_RadialCV_Brightfield_2of4	1	rs2994946	84587455	G	T	0.41	-0.19	0.03	3.17E-08	isolate	RadialDistribution
Nuclei_RadialDistribution_RadialCV_Brightfield_2of4	1	rs2945149	84588891	T	C	0.41	-0.19	0.03	3.66E-08	isolate	RadialDistribution
Nuclei_RadialDistribution_RadialCV_Brightfield_2of4	1	rs2943669	84591783	G	A	0.41	-0.19	0.03	3.17E-08	isolate	RadialDistribution
Nuclei_RadialDistribution_RadialCV_Brightfield_2of4	1	rs2943670	84591842	G	A	0.41	-0.19	0.03	3.17E-08	isolate	RadialDistribution
Nuclei_RadialDistribution_RadialCV_Brightfield_2of4	1	rs2945150	84592083	A	G	0.41	-0.19	0.03	3.17E-08	isolate	RadialDistribution
Nuclei_RadialDistribution_RadialCV_Brightfield_2of4	1	rs2945146	84597055	A	G	0.41	-0.19	0.03	3.17E-08	isolate	RadialDistribution
Nuclei_RadialDistribution_RadialCV_Brightfield_2of4	1	rs2945148	84599675	C	T	0.41	-0.19	0.03	3.17E-08	isolate	RadialDistribution
Nuclei_RadialDistribution_RadialCV_Brightfield_2of4	1	rs2911603	84605194	C	T	0.41	-0.19	0.03	3.17E-08	isolate	RadialDistribution
Nuclei_RadialDistribution_RadialCV_Brightfield_2of4	1	rs2911604	84606921	G	A	0.41	-0.19	0.03	3.17E-08	isolate	RadialDistribution
Nuclei_RadialDistribution_RadialCV_Brightfield_2of4	1	rs2943663	84611224	C	G	0.41	-0.19	0.03	3.17E-08	isolate	RadialDistribution
Nuclei_RadialDistribution_RadialCV_Brightfield_2of4	1	rs2911605	84611273	G	C	0.41	-0.19	0.03	3.17E-08	isolate	RadialDistribution
Nuclei_RadialDistribution_RadialCV_Brightfield_2of4	1	rs2945142	84611324	C	G	0.41	-0.19	0.03	3.17E-08	isolate	RadialDistribution
Nuclei_RadialDistribution_RadialCV_Brightfield_2of4	1	rs1850508	84611585	A	G	0.41	-0.19	0.03	3.17E-08	isolate	RadialDistribution
Nuclei_RadialDistribution_RadialCV_Brightfield_2of4	1	rs1850509	84611977	G	A	0.41	-0.19	0.03	3.17E-08	isolate	RadialDistribution

Nuclei_RadialDistribution_RadialCV_Brightfield_2of4	1	rs2007454	84613727	A	C	0.41	-0.19	0.03	2.12E-08	isolate	RadialDistribution
Nuclei_RadialDistribution_RadialCV_Brightfield_2of4	1	rs2945140	84615152	A	C	0.41	-0.19	0.03	3.17E-08	isolate	RadialDistribution
Cells_AreaShape_Zernike_5_3	1	rs9433603	87591608	A	G	0.58	0.29	0.05	3.99E-08	intcol	AreaShape
Cells_Texture_InfoMeas1_AGP_10_00	1	1:123982354_C_T	123982354	C	T	0.08	-0.64	0.10	5.70E-10	isolate	Texture
Cells_Texture_InfoMeas1_AGP_10_01	1	1:123982354_C_T	123982354	C	T	0.08	-0.58	0.10	8.67E-09	isolate	Texture
Nuclei_RadialDistribution_MeanFrac_Brightfield_3of4	1	rs12125619	210214217	A	G	0.34	0.26	0.05	2.09E-08	isolate	RadialDistribution
Cytoplasm_Texture_AngularSecondMoment_ER_20_00	1	rs6672161	225753329	G	A	0.06	0.39	0.07	1.05E-08	isolate	Texture
Cytoplasm_Texture_AngularSecondMoment_ER_20_00	1	rs111314906	225754472	C	T	0.06	0.39	0.07	9.04E-09	isolate	Texture
Cytoplasm_Texture_AngularSecondMoment_ER_20_00	1	rs79225203	225755013	T	G	0.06	0.39	0.07	1.05E-08	isolate	Texture
Cytoplasm_Texture_AngularSecondMoment_ER_20_00	1	rs114951309	225758899	T	C	0.06	0.39	0.07	1.05E-08	isolate	Texture
Cytoplasm_Texture_AngularSecondMoment_ER_20_00	1	rs6695625	225759524	C	T	0.06	0.39	0.07	1.05E-08	isolate	Texture
Cytoplasm_Texture_AngularSecondMoment_ER_20_00	1	rs6693328	225759829	G	A	0.06	0.39	0.07	1.05E-08	isolate	Texture
Cytoplasm_Texture_AngularSecondMoment_ER_20_00	1	rs199562211	225760680	AC	A	0.06	0.39	0.07	1.05E-08	isolate	Texture
Cytoplasm_Texture_AngularSecondMoment_ER_20_00	1	rs7538185	225761721	C	T	0.06	0.39	0.07	1.05E-08	isolate	Texture
Cytoplasm_Texture_AngularSecondMoment_ER_20_00	1	rs76832850	225764637	G	C	0.06	0.39	0.07	1.05E-08	isolate	Texture
Cytoplasm_Texture_AngularSecondMoment_ER_20_00	1	rs74419726	225766669	A	C	0.06	0.39	0.07	1.05E-08	isolate	Texture
Cytoplasm_Texture_AngularSecondMoment_ER_20_00	1	rs78799711	225766778	T	C	0.06	0.39	0.07	1.05E-08	isolate	Texture
Cytoplasm_Texture_AngularSecondMoment_ER_20_00	1	rs7520182	225767948	A	G	0.05	0.40	0.07	1.49E-08	isolate	Texture
Cytoplasm_Texture_DifferenceVariance_ER_20_03	1	rs7520182	225767948	A	G	0.05	0.41	0.07	3.22E-08	isolate	Texture
Cells_RadialDistribution_MeanFrac_ER_4of4	1	rs10925758	238766029	A	C	0.24	0.24	0.04	3.74E-08	isolate	RadialDistribution
Cells_RadialDistribution_MeanFrac_ER_4of4	1	rs10925759	238766046	C	T	0.24	0.24	0.04	3.74E-08	isolate	RadialDistribution
Cells_AreaShape_Zernike_3_1	2	rs11888919	1544686	A	G	0.05	-0.60	0.11	1.29E-08	intcol	AreaShape
Cells_AreaShape_Zernike_3_1	2	rs74770083	1545849	T	C	0.05	-0.60	0.11	1.29E-08	intcol	AreaShape
Cells_AreaShape_Zernike_3_1	2	rs11903370	1557197	G	C	0.05	-0.60	0.11	1.29E-08	intcol	AreaShape
Cells_AreaShape_Zernike_3_1	2	rs150903536	1561002	A	G	0.06	-0.53	0.10	1.93E-08	intcol	AreaShape
Cells_AreaShape_Zernike_3_1	2	rs11901668	1564008	C	G	0.06	-0.56	0.10	2.44E-08	intcol	AreaShape
Nuclei_Granularity_2_Brightfield	2	rs79553122	62845046	G	A	0.07	-0.48	0.09	2.59E-08	isolate	Granularity
Nuclei_Granularity_2_Brightfield	2	rs111306921	62849464	A	C	0.07	-0.48	0.09	2.59E-08	isolate	Granularity
Cells_AreaShape_Zernike_6_4	2	rs17754447	76268767	C	T	0.18	-0.34	0.06	9.76E-10	isolate	AreaShape
Cells_AreaShape_Zernike_6_4	2	rs17754897	76282738	A	G	0.18	-0.33	0.06	4.44E-09	isolate	AreaShape
Cells_AreaShape_Zernike_8_6	2	rs17754897	76282738	A	G	0.18	-0.29	0.05	1.23E-08	isolate	AreaShape
Cells_AreaShape_Zernike_6_4	2	rs1526619	76289879	G	A	0.17	-0.32	0.06	2.21E-08	isolate	AreaShape
Cells_AreaShape_Zernike_8_6	2	rs1526619	76289879	G	A	0.17	-0.29	0.05	2.40E-08	isolate	AreaShape
Cells_AreaShape_Zernike_9_1	2	2:113608872_C_T	113608872	C	T	0.09	-0.33	0.06	4.42E-09	isolate	AreaShape
Cells_Intensity_IntegratedIntensity_Brightfield	2	rs76117428	156111072	G	A	0.08	0.46	0.08	1.11E-08	intcol	Intensity

Cells_Intensity_IntegratedIntensity_Brightfield	2	rs72902129	156112915	A	G	0.08	0.46	0.08	1.27E-08	intcol	Intensity
Cells_Intensity_IntegratedIntensity_Brightfield	2	rs72902130	156114867	A	G	0.08	0.47	0.08	7.13E-09	intcol	Intensity
Cells_Intensity_IntegratedIntensity_Brightfield	2	rs6736241	156118947	T	C	0.08	0.46	0.08	1.11E-08	intcol	Intensity
Cells_Intensity_IntegratedIntensity_Brightfield	2	rs78646618	156124954	T	A	0.08	0.44	0.08	3.12E-08	intcol	Intensity
Cells_Intensity_IntegratedIntensity_Brightfield	2	rs16840025	156126079	A	T	0.08	0.46	0.08	1.11E-08	intcol	Intensity
Cells_Intensity_IntegratedIntensity_Brightfield	2	rs11413062	156129007	GA	G	0.08	0.44	0.08	3.39E-08	intcol	Intensity
Cells_Intensity_IntegratedIntensity_Brightfield	2	rs16840040	156129806	C	A	0.08	0.44	0.08	3.39E-08	intcol	Intensity
Cytoplasm_AreaShape_Zernike_2_0	3	rs71309872	2170212	A	C	0.15	0.33	0.06	1.84E-08	intcol	AreaShape
Nuclei_Granularity_2_DNA	3	rs11714730	35411853	G	A	0.14	0.35	0.06	7.94E-10	isolate	Granularity
Nuclei_Granularity_2_DNA	3	rs4678773	35416445	A	G	0.13	0.34	0.06	2.05E-09	isolate	Granularity
Nuclei_Granularity_2_DNA	3	rs17033175	35418730	A	G	0.14	0.32	0.05	1.01E-08	isolate	Granularity
Nuclei_Granularity_2_DNA	3	rs116732107	35435507	C	T	0.09	0.41	0.07	3.80E-09	isolate	Granularity
Cytoplasm_RadialDistribution_FracAtD_DNA_4of4	3	rs2358617	53155422	C	T	0.70	0.30	0.05	3.00E-08	intcol	RadialDistribution
Cytoplasm_RadialDistribution_FracAtD_DNA_4of4	3	rs11130345	53155654	G	A	0.70	0.30	0.05	3.00E-08	intcol	RadialDistribution
Cytoplasm_Texture_InfoMeas1_AGP_10_03	3	rs16851797	105935296	T	G	0.05	0.38	0.07	9.28E-09	isolate	Texture
Cells_Intensity_LowerQuartileIntensity_RNA	4	rs74336915	170945807	G	C	0.06	0.31	0.06	3.41E-08	intcol	Intensity
Nuclei_Intensity_MassDisplacement_RNA	5	rs901508	2006893	T	C	0.12	-0.31	0.06	3.66E-08	isolate	Intensity
Nuclei_RadialDistribution_MeanFrac_RNA_2of4	5	rs13177725	6795651	G	A	0.23	0.20	0.04	3.25E-08	isolate	RadialDistribution
Cells_Texture_InfoMeas1_AGP_5_00	5	rs6888188	31981299	C	G	0.35	-0.18	0.03	2.60E-08	intcol	Texture
Nuclei_Granularity_7_Mito	5	5:49820443_A_G	49820443	G	A	0.16	0.37	0.07	3.59E-08	intcol	Granularity
Nuclei_Granularity_1_DNA	5	rs168773	179202360	A	T	0.64	-0.15	0.03	1.88E-08	intcol	Granularity
Nuclei_Granularity_7_Mito	6	6:2683405_G_T	2683405	T	G	0.08	-0.52	0.09	5.07E-09	isolate	Granularity
Cytoplasm_RadialDistribution_RadialCV_Brightfield_1of4	6	rs9343870	79079756	G	T	0.36	0.26	0.05	3.32E-08	isolate	RadialDistribution
Cells_Texture_InfoMeas1_AGP_10_00	6	rs6557219	152422313	A	G	0.23	-0.23	0.04	4.03E-08	intcol	Texture
Cells_Texture_InfoMeas1_AGP_10_00	6	rs6913500	152422872	T	C	0.23	-0.23	0.04	4.03E-08	intcol	Texture
Cells_Granularity_1_ER	6	rs827967	158401447	T	C	0.32	-0.22	0.04	2.15E-08	isolate	Granularity
Cells_Granularity_1_ER	6	rs1758972	158403142	A	G	0.68	0.22	0.04	2.15E-08	isolate	Granularity
Cells_Granularity_1_ER	6	rs1754419	158403146	G	A	0.68	0.22	0.04	2.15E-08	isolate	Granularity
Nuclei_RadialDistribution_RadialCV_DNA_1of4	6	rs78212918	169255468	G	A	0.08	0.39	0.07	3.36E-08	isolate	RadialDistribution
Nuclei_Granularity_9_AGP	7	rs36036340	575905	A	G	0.08	0.38	0.06	6.02E-10	intcol	Granularity
Nuclei_Granularity_2_DNA	7	rs12720007	643957	A	G	0.16	0.32	0.05	1.28E-09	isolate	Granularity
Cells_Intensity_IntegratedIntensityEdge_RNA	7	rs202231415	57635662	G	A	0.06	0.54	0.09	1.14E-09	intcol	Intensity
Cells_Texture_Entropy_AGP_10_01	7	rs62498587	145359153	G	A	0.26	-0.16	0.03	2.67E-09	isolate	Texture
Cells_Texture_Entropy_AGP_10_01	7	rs10268673	145360956	T	C	0.26	-0.16	0.03	3.23E-09	isolate	Texture
Cells_Texture_Entropy_AGP_10_01	7	rs10227393	145365647	G	C	0.25	-0.16	0.03	1.98E-09	isolate	Texture
Cells_Texture_Entropy_AGP_10_01	7	rs10245974	145365873	C	T	0.26	-0.16	0.03	3.23E-09	isolate	Texture
Cells_Texture_Entropy_AGP_10_01	7	rs10230532	145371805	G	C	0.22	-0.15	0.03	2.55E-08	isolate	Texture

Cells_Texture_Entropy_AGP_10_01	7	rs5888163	145373442	A	AT	0.21	-0.15	0.03	2.22E-08	isolate	Texture
Cells_Granularity_1_Mito	7	rs1528016	145386700	T	C	0.09	0.40	0.07	2.10E-08	isolate	Granularity
Cytoplasm_Intensity_StdIntensityEdge_Mito	7	rs1528016	145386700	T	C	0.09	-0.44	0.07	5.05E-10	isolate	Intensity
Cells_Granularity_1_Mito	7	rs2023255	145393195	T	C	0.09	0.40	0.07	2.10E-08	isolate	Granularity
Cytoplasm_Intensity_StdIntensityEdge_Mito	7	rs2023255	145393195	T	C	0.09	-0.44	0.07	5.05E-10	isolate	Intensity
Cytoplasm_Intensity_StdIntensityEdge_Mito	7	rs369476630	145418562	A	AG	0.10	-0.41	0.07	3.87E-09	isolate	Intensity
Cells_RadialDistribution_MeanFrac_AGP_2of4	8	rs2554525	4056013	G	A	0.90	0.28	0.05	6.61E-09	isolate	RadialDistribution
Cells_RadialDistribution_MeanFrac_AGP_2of4	8	rs77500874	4057499	T	C	0.89	0.27	0.05	2.29E-08	isolate	RadialDistribution
Cells_RadialDistribution_MeanFrac_AGP_2of4	8	rs4416841	4059963	A	T	0.90	0.28	0.05	1.60E-08	isolate	RadialDistribution
Cells_RadialDistribution_MeanFrac_AGP_2of4	8	rs5889013	4061064	C	CATTTA	0.90	0.28	0.05	1.60E-08	isolate	RadialDistribution
Cells_RadialDistribution_MeanFrac_AGP_2of4	8	rs2911720	4061150	T	C	0.90	0.28	0.05	1.60E-08	isolate	RadialDistribution
Cells_RadialDistribution_MeanFrac_AGP_2of4	8	rs2216893	4061302	T	C	0.90	0.28	0.05	1.60E-08	isolate	RadialDistribution
Cells_Granularity_6_Mito	8	rs73239317	27939039	A	G	0.06	0.59	0.11	2.81E-08	isolate	Granularity
Cells_AreaShape_Zernike_4_2	8	rs71525174	72532825	C	T	0.12	-0.35	0.06	3.59E-09	isolate	AreaShape
Cytoplasm_AreaShape_Zernike_4_2	8	rs71525174	72532825	C	T	0.12	-0.31	0.05	2.41E-09	isolate	AreaShape
Cells_AreaShape_MeanRadius	8	rs4734466	100595612	C	G	0.13	-0.47	0.09	3.75E-08	intcol	AreaShape
Cells_Texture_InfoMeas1_RNA_5_01	8	rs10093391	114226111	G	A	0.09	0.32	0.06	2.30E-08	isolate	Texture
Cells_Texture_InfoMeas1_RNA_5_01	8	rs11783619	114227077	A	G	0.09	0.32	0.06	2.30E-08	isolate	Texture
Cells_Texture_InfoMeas1_RNA_5_01	8	rs66484064	114228456	C	T	0.09	0.32	0.06	2.30E-08	isolate	Texture
Cells_Texture_InfoMeas1_RNA_5_01	8	rs10087988	114229934	T	A	0.09	0.32	0.06	2.30E-08	isolate	Texture
Cells_Texture_InfoMeas1_RNA_5_01	8	rs10088231	114230124	T	A	0.09	0.32	0.06	2.30E-08	isolate	Texture
Cells_Texture_InfoMeas1_RNA_5_01	8	rs10092402	114231218	T	C	0.09	0.32	0.06	2.30E-08	isolate	Texture
Cells_Texture_InfoMeas1_RNA_5_01	8	rs10087011	114233156	G	T	0.09	0.32	0.06	2.30E-08	isolate	Texture
Cells_Texture_InfoMeas1_RNA_5_01	8	rs10107869	114234910	C	A	0.09	0.32	0.06	2.43E-08	isolate	Texture
Cells_Texture_InfoMeas2_RNA_5_03	8	rs10088012	114236963	A	G	0.09	-0.36	0.07	3.88E-08	isolate	Texture
Cells_Texture_InfoMeas1_RNA_5_01	8	rs10088012	114236963	A	G	0.09	0.33	0.06	8.45E-09	isolate	Texture
Cells_Texture_InfoMeas2_RNA_5_03	8	rs28417833	114269860	G	C	0.09	-0.37	0.07	3.59E-08	isolate	Texture
Cells_Texture_InfoMeas1_RNA_5_01	8	rs28417833	114269860	G	C	0.09	0.34	0.06	1.01E-08	isolate	Texture
Cells_Texture_InfoMeas2_RNA_5_03	8	rs28406718	114280858	T	G	0.09	-0.37	0.07	2.28E-08	isolate	Texture
Cells_Texture_InfoMeas1_RNA_5_01	8	rs28406718	114280858	T	G	0.09	0.33	0.06	1.11E-08	isolate	Texture
Cells_Texture_InfoMeas2_RNA_5_03	8	rs34217691	114287846	T	TATC	0.09	-0.38	0.07	2.55E-08	isolate	Texture
Cells_Texture_InfoMeas1_RNA_5_01	8	rs34217691	114287846	T	TATC	0.09	0.33	0.06	1.55E-08	isolate	Texture
Cells_Texture_InfoMeas2_RNA_5_03	8	rs6982069	114302696	C	G	0.08	-0.39	0.07	9.56E-09	isolate	Texture
Cells_Texture_InfoMeas1_RNA_5_01	8	rs6982069	114302696	C	G	0.08	0.35	0.06	4.10E-09	isolate	Texture
Cells_Texture_InfoMeas2_RNA_5_03	8	rs148620046	114303196	A	AC	0.09	-0.37	0.07	3.66E-08	isolate	Texture
Cells_Texture_InfoMeas1_RNA_5_01	8	rs148620046	114303196	A	AC	0.09	0.33	0.06	2.54E-08	isolate	Texture

Cells_Texture_InfoMeas2_RNA_5_03	8	rs67235795	114303372	A	T	0.09	-0.38	0.07	2.91E-08	isolate	Texture
Cells_Texture_InfoMeas1_RNA_5_01	8	rs67235795	114303372	A	T	0.09	0.34	0.06	1.76E-08	isolate	Texture
Cells_Texture_InfoMeas2_RNA_5_03	8	rs7007928	114304037	C	T	0.09	-0.38	0.07	2.55E-08	isolate	Texture
Cells_Texture_InfoMeas1_RNA_5_01	8	rs7007928	114304037	C	T	0.09	0.33	0.06	1.55E-08	isolate	Texture
Cells_Texture_InfoMeas2_RNA_5_03	8	rs13439875	114311660	G	C	0.08	-0.39	0.07	9.56E-09	isolate	Texture
Cells_Texture_InfoMeas1_RNA_5_01	8	rs13439875	114311660	G	C	0.08	0.35	0.06	4.10E-09	isolate	Texture
Cells_Texture_InfoMeas2_RNA_5_03	8	rs2132788	114315194	T	A	0.09	-0.37	0.07	3.20E-08	isolate	Texture
Cells_Texture_InfoMeas1_RNA_5_01	8	rs2132788	114315194	T	A	0.09	0.33	0.06	2.24E-08	isolate	Texture
Cells_Texture_InfoMeas2_RNA_5_03	8	rs2132789	114315284	G	A	0.09	-0.37	0.07	3.20E-08	isolate	Texture
Cells_Texture_InfoMeas1_RNA_5_01	8	rs2132789	114315284	G	A	0.09	0.33	0.06	2.24E-08	isolate	Texture
Cells_Texture_InfoMeas2_RNA_5_03	8	rs28540774	114317993	T	C	0.09	-0.38	0.07	2.91E-08	isolate	Texture
Cells_Texture_InfoMeas1_RNA_5_01	8	rs28540774	114317993	T	C	0.09	0.34	0.06	1.76E-08	isolate	Texture
Cells_Texture_InfoMeas2_RNA_5_03	8	rs143974673	114320404	TG	T	0.09	-0.37	0.07	3.20E-08	isolate	Texture
Cells_Texture_InfoMeas1_RNA_5_01	8	rs143974673	114320404	TG	T	0.09	0.33	0.06	2.24E-08	isolate	Texture
Cells_Texture_InfoMeas2_RNA_5_03	8	rs9656904	114335691	C	T	0.09	-0.39	0.07	8.43E-09	isolate	Texture
Cells_Texture_InfoMeas1_RNA_5_01	8	rs9656904	114335691	C	T	0.09	0.35	0.06	3.65E-09	isolate	Texture
Nuclei_Intensity_MedianIntensity_Mito	9	rs7040052	29335833	G	A	0.39	0.24	0.04	2.28E-08	isolate	Intensity
Cells_AreaShape_Zernike_7_3	9	9:61730492_A_G	61730492	A	G	0.14	-0.31	0.06	3.40E-08	isolate	AreaShape
Cells_Intensity_IntegratedIntensity_RNA	9	rs4743956	94514473	T	C	0.76	-0.22	0.04	1.60E-08	intcol	Intensity
Nuclei_RadialDistribution_FracAtD_RNA_3of4	9	rs13290729	103815560	T	C	0.53	-0.17	0.03	2.56E-08	isolate	RadialDistribution
Cytoplasm_RadialDistribution_MeanFrac_DNA_1of4	9	rs113816904	122460196	T	C	0.83	0.35	0.06	1.86E-08	intcol	RadialDistribution
Nuclei_Intensity_StdIntensityEdge_DNA	10	rs10904736	16435478	T	C	0.06	-0.20	0.03	5.93E-09	isolate	Intensity
Nuclei_Intensity_StdIntensityEdge_DNA	10	10:16476904_C_T	16476904	C	T	0.06	-0.21	0.04	1.12E-08	isolate	Intensity
Cells_Granularity_7_Brightfield	10	rs117246203	18362818	A	G	0.14	0.32	0.06	1.66E-08	intcol	Granularity
Nuclei_Texture_SumAverage_Brightfield_20_01	10	rs1248990	28736749	A	C	0.22	-0.16	0.03	2.38E-08	intcol	Texture
Cells_AreaShape_MaximumRadius	10	rs2778665	31768178	G	A	0.40	0.33	0.05	1.29E-09	intcol	AreaShape
Cells_AreaShape_MeanRadius	10	rs2778665	31768178	G	A	0.40	0.32	0.06	3.91E-08	intcol	AreaShape
Cytoplasm_Intensity_StdIntensityEdge_Mito	10	10:41575547_G_T	41575547	G	T	0.13	-0.37	0.06	8.63E-09	intcol	Intensity
Nuclei_Texture_InfoMeas1_RNA_5_00	10	rs56156031	60338941	T	C	0.24	0.21	0.04	4.08E-08	intcol	Texture
Nuclei_Texture_InfoMeas1_RNA_20_03	10	rs56156031	60338941	T	C	0.24	0.24	0.04	2.49E-08	intcol	Texture
Nuclei_Texture_InfoMeas1_RNA_5_00	10	rs55976329	60339030	A	G	0.24	0.21	0.04	4.08E-08	intcol	Texture
Nuclei_Texture_InfoMeas1_RNA_20_03	10	rs55976329	60339030	A	G	0.24	0.24	0.04	2.49E-08	intcol	Texture
Nuclei_Texture_InfoMeas1_RNA_5_00	10	rs1903203	60340406	A	T	0.24	0.21	0.04	1.45E-08	intcol	Texture
Nuclei_Texture_InfoMeas1_RNA_20_03	10	rs1903203	60340406	A	T	0.24	0.25	0.04	1.14E-08	intcol	Texture
Nuclei_Texture_InfoMeas1_RNA_5_00	10	rs113292084	60340641	C	G	0.24	0.22	0.04	7.54E-09	intcol	Texture
Nuclei_Texture_InfoMeas1_RNA_20_03	10	rs113292084	60340641	C	G	0.24	0.26	0.04	6.22E-09	intcol	Texture

Nuclei_Texture_InfoMeas1_RNA_5_00	10	rs79644767	60341478	T	A	0.24	0.21	0.04	1.45E-08	intcol	Texture
Nuclei_Texture_InfoMeas1_RNA_20_03	10	rs79644767	60341478	T	A	0.24	0.25	0.04	1.14E-08	intcol	Texture
Nuclei_Texture_InfoMeas1_RNA_5_00	10	rs55749838	60345290	A	C	0.24	0.21	0.04	4.08E-08	intcol	Texture
Nuclei_Texture_InfoMeas1_RNA_20_03	10	rs55749838	60345290	A	C	0.24	0.24	0.04	2.49E-08	intcol	Texture
Nuclei_Texture_InfoMeas1_RNA_20_03	10	rs78908423	60346538	T	C	0.24	0.24	0.04	3.95E-08	intcol	Texture
Nuclei_Texture_InfoMeas1_RNA_5_00	10	rs77914050	60349441	C	T	0.24	0.21	0.04	4.08E-08	intcol	Texture
Nuclei_Texture_InfoMeas1_RNA_20_03	10	rs77914050	60349441	C	T	0.24	0.24	0.04	2.49E-08	intcol	Texture
Nuclei_Texture_InfoMeas1_RNA_20_03	10	rs78075604	60350591	T	C	0.24	0.24	0.04	3.95E-08	intcol	Texture
Nuclei_Texture_InfoMeas1_RNA_5_00	10	rs41494951	60352694	T	C	0.24	0.21	0.04	4.08E-08	intcol	Texture
Nuclei_Texture_InfoMeas1_RNA_20_03	10	rs41494951	60352694	T	C	0.24	0.24	0.04	2.49E-08	intcol	Texture
Nuclei_Texture_InfoMeas1_RNA_5_00	10	rs79603065	60353906	A	G	0.24	0.21	0.04	4.08E-08	intcol	Texture
Nuclei_Texture_InfoMeas1_RNA_20_03	10	rs79603065	60353906	A	G	0.24	0.24	0.04	2.49E-08	intcol	Texture
Nuclei_Texture_InfoMeas1_RNA_20_03	10	rs150574912	60355846	GTAAGGATTTTCTTCC	G	0.24	0.25	0.04	2.18E-08	intcol	Texture
Nuclei_Texture_InfoMeas1_RNA_5_00	10	rs117443225	60363079	T	C	0.25	0.22	0.04	4.57E-09	intcol	Texture
Nuclei_Texture_InfoMeas1_RNA_20_03	10	rs117443225	60363079	T	C	0.25	0.25	0.04	4.40E-09	intcol	Texture
Cells_AreaShape_Zernike_5_5	10	rs4918911	95324222	G	C	0.67	0.28	0.05	3.68E-08	intcol	AreaShape
Nuclei_AreaShape_Zernike_8_6	10	rs17207435	105339859	G	A	0.09	-0.60	0.10	8.42E-09	intcol	AreaShape
Cells_RadialDistribution_MeanFrac_Brighfield_3of4	10	rs4752117	117826667	C	T	0.64	-0.30	0.05	2.83E-08	intcol	RadialDistribution
Cells_RadialDistribution_MeanFrac_Brighfield_3of4	10	rs4431946	117826951	C	A	0.62	-0.29	0.05	2.63E-08	intcol	RadialDistribution
Cells_RadialDistribution_MeanFrac_Brighfield_3of4	10	rs4752118	117827452	C	T	0.64	-0.29	0.05	2.86E-08	intcol	RadialDistribution
Cells_RadialDistribution_MeanFrac_Brighfield_3of4	10	rs11198135	117831657	C	A	0.65	-0.30	0.05	1.24E-08	intcol	RadialDistribution
Cells_RadialDistribution_MeanFrac_Brighfield_3of4	10	rs4752122	117832976	A	G	0.62	-0.29	0.05	2.26E-08	intcol	RadialDistribution
Cells_RadialDistribution_MeanFrac_Brighfield_3of4	10	rs199983705	117837435	T	C	0.62	-0.29	0.05	2.63E-08	intcol	RadialDistribution
Cells_RadialDistribution_MeanFrac_Brighfield_3of4	10	rs11198141	117838535	T	C	0.56	-0.31	0.05	2.04E-08	intcol	RadialDistribution
Cells_AreaShape_Zernike_6_4	10	rs11244871	126183597	A	G	0.22	0.38	0.07	3.31E-08	intcol	AreaShape
Cells_AreaShape_Zernike_4_2	10	rs11244871	126183597	A	G	0.22	0.37	0.07	1.70E-08	intcol	AreaShape
Cells_AreaShape_Zernike_6_4	10	rs1459709	126184259	A	G	0.22	0.38	0.07	3.31E-08	intcol	AreaShape
Cells_AreaShape_Zernike_4_2	10	rs1459709	126184259	A	G	0.22	0.37	0.07	1.70E-08	intcol	AreaShape
Cells_AreaShape_Zernike_4_2	10	rs17683203	126186844	A	G	0.22	0.37	0.07	3.09E-08	intcol	AreaShape
Nuclei_Granularity_6_ER	11	rs9651588	26025266	C	A	0.42	-0.19	0.03	2.64E-08	intcol	Granularity
Nuclei_Granularity_6_ER	11	rs7938796	26030632	G	A	0.46	-0.20	0.03	4.87E-09	intcol	Granularity
Nuclei_Granularity_6_ER	11	rs11029110	26032525	C	T	0.45	-0.20	0.03	3.34E-09	intcol	Granularity
Nuclei_Granularity_6_ER	11	rs11029111	26032705	G	C	0.45	-0.20	0.03	3.34E-09	intcol	Granularity
Nuclei_Granularity_6_ER	11	rs7951212	26033790	T	C	0.44	-0.19	0.03	3.45E-08	intcol	Granularity
Nuclei_Granularity_6_ER	11	rs7127255	26041603	C	T	0.46	-0.20	0.03	5.86E-09	intcol	Granularity
Nuclei_Granularity_6_ER	11	rs7105591	26073810	C	T	0.48	-0.18	0.03	3.90E-08	intcol	Granularity

Cells_AreaShape_Eccentricity	11	rs729120	72723073	T	C	0.21	-0.35	0.06	2.91E-08	isolate	AreaShape
Cytoplasm_RadialDistribution_RadialCV_ER_2of4	11	rs144391443	99134735	A	ACCAT	0.14	-0.39	0.07	2.99E-08	isolate	RadialDistribution
Cytoplasm_RadialDistribution_RadialCV_ER_2of4	11	rs59260299	99136142	C	T	0.14	-0.39	0.07	2.99E-08	isolate	RadialDistribution
Cytoplasm_RadialDistribution_RadialCV_ER_2of4	11	rs4282949	99136454	G	A	0.14	-0.39	0.07	2.99E-08	isolate	RadialDistribution
Cytoplasm_RadialDistribution_RadialCV_ER_2of4	11	rs1893151	99137347	C	G	0.14	-0.39	0.07	2.81E-08	isolate	RadialDistribution
Cytoplasm_RadialDistribution_RadialCV_ER_2of4	11	rs56082009	99139055	T	A	0.14	-0.39	0.07	2.99E-08	isolate	RadialDistribution
Cytoplasm_RadialDistribution_RadialCV_ER_2of4	11	rs2897525	99191904	A	C	0.23	-0.32	0.06	3.15E-08	isolate	RadialDistribution
Cytoplasm_RadialDistribution_RadialCV_RNA_2of4	11	rs2897525	99191904	A	C	0.23	-0.30	0.05	3.57E-08	isolate	RadialDistribution
Nuclei_RadialDistribution_RadialCV_Brightfield_2of4	11	rs10895507	103676418	G	A	0.57	0.21	0.04	2.25E-09	isolate	RadialDistribution
Cells_Intensity_IntegratedIntensity_Mito	11	rs6589125	110355052	A	T	0.25	0.33	0.06	2.84E-09	isolate	Intensity
Cells_Intensity_IntegratedIntensity_Mito	11	rs4753886	110355530	A	T	0.25	0.33	0.06	2.84E-09	isolate	Intensity
Cells_Intensity_IntegratedIntensity_Mito	11	rs969680	110355722	C	T	0.25	0.33	0.06	2.84E-09	isolate	Intensity
Nuclei_RadialDistribution_RadialCV_DNA_3of4	12	rs36011055	12047660	G	A	0.16	-0.22	0.04	2.17E-08	intcol	RadialDistribution
Nuclei_RadialDistribution_MeanFrac_Mito_4of4	12	rs11171902	39376846	T	C	0.10	-0.35	0.06	1.48E-08	intcol	RadialDistribution
Nuclei_RadialDistribution_MeanFrac_Mito_4of4	12	rs117819007	39416590	A	G	0.10	-0.33	0.06	3.87E-08	intcol	RadialDistribution
Nuclei_RadialDistribution_MeanFrac_Mito_4of4	12	rs74755643	39453947	G	A	0.10	-0.38	0.06	2.33E-09	intcol	RadialDistribution
Nuclei_RadialDistribution_MeanFrac_Mito_4of4	12	rs58754883	39496861	G	T	0.10	-0.38	0.06	2.33E-09	intcol	RadialDistribution
Nuclei_RadialDistribution_MeanFrac_Mito_4of4	12	rs79906550	39503388	A	G	0.10	-0.38	0.06	2.33E-09	intcol	RadialDistribution
Nuclei_RadialDistribution_MeanFrac_Mito_4of4	12	rs139937401	39509017	TA	T	0.10	-0.38	0.06	2.33E-09	intcol	RadialDistribution
Cytoplasm_RadialDistribution_RadialCV_DNA_2of4	12	rs17116870	55053776	T	A	0.05	0.53	0.09	7.27E-10	intcol	RadialDistribution
Cells_AreaShape_Zernike_6_4	12	rs10878843	68491019	A	G	0.21	-0.31	0.05	2.12E-09	isolate	AreaShape
Cells_AreaShape_Zernike_6_4	12	rs7960007	68491363	G	A	0.21	-0.30	0.05	5.10E-09	isolate	AreaShape
Cells_AreaShape_Zernike_6_4	12	rs1861932	68492753	C	T	0.20	-0.29	0.05	3.22E-08	isolate	AreaShape
Cells_AreaShape_Zernike_6_4	12	rs7961621	68493176	C	T	0.20	-0.30	0.05	1.50E-08	isolate	AreaShape
Cells_AreaShape_Zernike_6_4	12	rs7975398	68493557	T	C	0.20	-0.30	0.05	1.50E-08	isolate	AreaShape
Cells_AreaShape_Zernike_6_4	12	rs11451958	68493728	CT	C	0.20	-0.30	0.05	1.50E-08	isolate	AreaShape
Cells_AreaShape_Zernike_6_4	12	rs7978683	68493915	A	C	0.20	-0.33	0.05	1.07E-09	isolate	AreaShape
Cells_AreaShape_Zernike_6_4	12	rs12425991	68494026	A	T	0.20	-0.30	0.05	1.50E-08	isolate	AreaShape
Cells_AreaShape_Zernike_6_4	12	rs10784720	68495124	G	T	0.21	-0.31	0.05	4.23E-09	isolate	AreaShape
Cells_Texture_InfoMeas1_AGP_10_00	12	12:70522881_G_T	70522881	T	G	0.06	0.47	0.09	2.69E-08	intcol	Texture
Cells_Texture_InfoMeas1_ER_5_00	12	rs77014851	77720475	T	G	0.05	-0.40	0.07	3.07E-08	intcol	Texture
Cells_Texture_InfoMeas1_ER_5_00	12	rs112347431	77720484	G	A	0.05	-0.40	0.07	3.07E-08	intcol	Texture

Cells_AreaShape_Zernike_9_1	13	rs9652061	20272033	T	C	0.28	0.18	0.03	4.01E-08	intcol	AreaShape
Nuclei_Intensity_MinIntensity_ER	13	rs139097437	41711682	G	A	0.05	0.45	0.08	2.18E-08	isolate	Intensity
Nuclei_RadialDistribution_RadialCV_Brightfield_2of4	13	rs1562190	93570067	G	A	0.88	0.29	0.05	3.09E-08	isolate	RadialDistribution
Nuclei_RadialDistribution_RadialCV_Brightfield_2of4	13	rs1445255	93570456	A	T	0.88	0.29	0.05	3.09E-08	isolate	RadialDistribution
Nuclei_RadialDistribution_RadialCV_Brightfield_2of4	13	rs9524132	93571853	A	G	0.88	0.29	0.05	3.09E-08	isolate	RadialDistribution
Nuclei_RadialDistribution_RadialCV_Brightfield_2of4	13	rs3910920	93572347	C	G	0.88	0.29	0.05	3.09E-08	isolate	RadialDistribution
Nuclei_RadialDistribution_RadialCV_Brightfield_2of4	13	rs9524133	93573263	G	T	0.88	0.29	0.05	3.09E-08	isolate	RadialDistribution
Nuclei_RadialDistribution_RadialCV_Brightfield_2of4	13	rs9301897	93573305	T	C	0.87	0.31	0.05	4.55E-10	isolate	RadialDistribution
Nuclei_RadialDistribution_RadialCV_Brightfield_2of4	13	rs1822870	93577088	G	A	0.88	0.29	0.05	3.09E-08	isolate	RadialDistribution
Nuclei_RadialDistribution_RadialCV_Brightfield_2of4	13	rs34215831	93577491	A	AT	0.88	0.29	0.05	3.09E-08	isolate	RadialDistribution
Nuclei_RadialDistribution_RadialCV_Brightfield_2of4	13	rs7995706	93580622	A	G	0.88	0.29	0.05	3.09E-08	isolate	RadialDistribution
Nuclei_RadialDistribution_RadialCV_Brightfield_2of4	13	rs3843687	93582903	C	T	0.88	0.29	0.05	3.09E-08	isolate	RadialDistribution
Nuclei_RadialDistribution_RadialCV_Brightfield_2of4	13	rs9524136	93585063	G	A	0.76	0.23	0.04	2.21E-08	isolate	RadialDistribution
Nuclei_RadialDistribution_RadialCV_Brightfield_2of4	13	rs9524137	93585261	G	C	0.76	0.23	0.04	2.94E-08	isolate	RadialDistribution
Nuclei_RadialDistribution_RadialCV_Brightfield_2of4	13	rs1037071	93587765	G	A	0.76	0.23	0.04	2.21E-08	isolate	RadialDistribution
Nuclei_RadialDistribution_RadialCV_Brightfield_2of4	13	rs1037072	93587829	A	G	0.76	0.23	0.04	2.21E-08	isolate	RadialDistribution
Nuclei_RadialDistribution_RadialCV_Brightfield_2of4	13	rs2389009	93588834	G	A	0.88	0.29	0.05	3.09E-08	isolate	RadialDistribution
Nuclei_RadialDistribution_RadialCV_Brightfield_2of4	13	rs5805812	93589273	C	CA	0.88	0.29	0.05	3.09E-08	isolate	RadialDistribution
Nuclei_RadialDistribution_RadialCV_Brightfield_2of4	13	rs9516257	93589891	C	A	0.87	0.28	0.05	2.80E-08	isolate	RadialDistribution
Nuclei_RadialDistribution_RadialCV_Brightfield_2of4	13	rs9561407	93591256	G	T	0.88	0.29	0.05	3.09E-08	isolate	RadialDistribution
Nuclei_RadialDistribution_RadialCV_Brightfield_2of4	13	rs4773752	93592383	A	C	0.88	0.29	0.05	3.09E-08	isolate	RadialDistribution
Nuclei_RadialDistribution_RadialCV_Brightfield_2of4	13	rs7984734	93593840	C	T	0.77	0.23	0.04	3.06E-08	isolate	RadialDistribution
Cytoplasm_AreaShape_Zernike_9_3	14	rs17105816	36964646	T	C	0.12	-0.36	0.06	3.62E-08	intcol	AreaShape
Cytoplasm_AreaShape_Zernike_9_1	14	rs142657238	46964982	A	G	0.05	-0.41	0.07	8.12E-09	isolate	AreaShape
Cytoplasm_RadialDistribution_MeanFrac_ER_2of4	14	rs60726667	55391205	C	G	0.13	0.37	0.07	1.54E-08	isolate	RadialDistribution
Nuclei_Granularity_2_DNA	14	14:75027679_A_AC	75027679	AC	A	0.48	-0.23	0.04	1.82E-08	isolate	Granularity
Cytoplasm_AreaShape_Zernike_2_0	14	rs117847322	83785142	T	C	0.08	-0.43	0.08	1.56E-08	intcol	AreaShape
Nuclei_RadialDistribution_RadialCV_Mito_3of4	14	rs6574878	86240824	G	C	0.17	-0.27	0.05	3.31E-08	intcol	RadialDistribution
Nuclei_RadialDistribution_RadialCV_Mito_3of4	14	rs17740684	86248001	C	T	0.17	-0.27	0.05	3.31E-08	intcol	RadialDistribution
Nuclei_RadialDistribution_RadialCV_Mito_3of4	14	rs61452450	86256223	T	TGAGA	0.18	-0.26	0.05	2.01E-08	intcol	RadialDistribution
Nuclei_RadialDistribution_RadialCV_Mito_3of4	14	rs11628193	86258331	T	G	0.18	-0.26	0.05	2.01E-08	intcol	RadialDistribution
Nuclei_RadialDistribution_RadialCV_Mito_3of4	14	rs372195291	86275328	T	TGAAC	0.18	-0.26	0.05	2.01E-08	intcol	RadialDistribution
Nuclei_RadialDistribution_RadialCV_Mito_3of4	14	rs1394702	86281777	G	T	0.18	-0.26	0.05	2.01E-08	intcol	RadialDistribution
Nuclei_RadialDistribution_RadialCV_Mito_3of4	14	rs1394701	86281993	A	T	0.18	-0.26	0.05	2.01E-08	intcol	RadialDistribution
Cells_Texture_SumEntropy_ER_5_01	14	rs60031701	101799719	A	G	0.13	0.35	0.06	3.88E-08	isolate	Texture
Cells_Granularity_9_Mito	16	rs8053436	70557954	G	A	0.46	0.19	0.03	2.80E-08	intcol	Granularity
Cells_Granularity_2_ER	17	rs2440041	1053530	G	A	0.12	0.33	0.06	3.37E-08	isolate	Granularity

Cells_Granularity_2_ER	17	rs112091943	1053842	C	T	0.12	0.33	0.06	3.37E-08	isolate	Granularity
Cells_Granularity_2_ER	17	rs2589492	1055442	T	C	0.12	0.33	0.06	3.47E-08	isolate	Granularity
Cells_AreaShape_Zernike_5_5	17	rs4791428	10849536	G	A	0.82	0.37	0.07	1.85E-08	intcol	AreaShape
Cytoplasm_RadialDistribution_MeanFrac_AGP_3of4	17	rs55852445	13837440	C	T	0.71	-0.38	0.07	6.97E-09	intcol	RadialDistribution
Cells_RadialDistribution_MeanFrac_ER_4of4	17	rs315506	31700056	T	A	0.92	-0.47	0.08	1.28E-09	intcol	RadialDistribution
Cytoplasm_RadialDistribution_RadialCV_ER_2of4	17	rs315506	31700056	T	A	0.92	0.55	0.09	6.60E-09	intcol	RadialDistribution
Cytoplasm_RadialDistribution_RadialCV_ER_3of4	17	rs315506	31700056	T	A	0.92	0.52	0.08	1.43E-10	intcol	RadialDistribution
Nuclei_Granularity_2_Brightfield	18	rs116862261	12354478	G	A	0.11	-0.40	0.07	3.94E-08	isolate	Granularity
Cytoplasm_Intensity_IntegratedIntensity_DNA	19	rs10414332	54031836	C	A	0.89	-0.28	0.05	1.68E-08	intcol	Intensity
Cytoplasm_Intensity_IntegratedIntensity_DNA	19	rs58904839	54031918	G	A	0.89	-0.28	0.05	1.68E-08	intcol	Intensity
Cytoplasm_Intensity_IntegratedIntensity_DNA	19	rs62145123	54031922	G	C	0.89	-0.28	0.05	1.68E-08	intcol	Intensity
Cytoplasm_Intensity_IntegratedIntensity_DNA	19	rs10414570	54031926	G	A	0.89	-0.28	0.05	1.56E-08	intcol	Intensity
Cytoplasm_Intensity_IntegratedIntensity_DNA	19	rs62145124	54031933	C	T	0.89	-0.28	0.05	1.56E-08	intcol	Intensity
Cytoplasm_Intensity_IntegratedIntensity_DNA	19	rs62145126	54031938	A	G	0.89	-0.28	0.05	1.68E-08	intcol	Intensity
Cytoplasm_Intensity_IntegratedIntensity_DNA	19	rs11667806	54032060	T	C	0.89	-0.28	0.05	1.68E-08	intcol	Intensity
Cytoplasm_Intensity_IntegratedIntensity_DNA	19	rs62145127	54032195	T	C	0.89	-0.28	0.05	1.68E-08	intcol	Intensity
Cytoplasm_Intensity_IntegratedIntensity_DNA	19	rs35474370	54032319	C	T	0.89	-0.28	0.05	1.68E-08	intcol	Intensity
Cytoplasm_Intensity_IntegratedIntensity_DNA	19	19:54032324_G_GTT	54032324	GTT	G	0.89	-0.28	0.05	1.68E-08	intcol	Intensity
Cytoplasm_Intensity_IntegratedIntensity_DNA	19	rs2077892	54032694	C	T	0.88	-0.28	0.05	7.89E-09	intcol	Intensity
Nuclei_RadialDistribution_FracAtD_Brightfield_4of4	19	rs2448290	54033732	C	T	0.88	-0.38	0.07	1.97E-08	isolate	RadialDistribution
Cells_RadialDistribution_MeanFrac_Brightfield_4of4	19	rs2448290	54033732	C	T	0.88	0.35	0.06	3.13E-08	intcol	RadialDistribution
Cytoplasm_Intensity_IntegratedIntensity_DNA	19	rs2448290	54033732	C	T	0.88	-0.29	0.05	1.37E-09	intcol	Intensity
Nuclei_RadialDistribution_FracAtD_Brightfield_4of4	19	rs4239595	54033901	A	C	0.88	-0.38	0.07	8.83E-09	isolate	RadialDistribution
Cytoplasm_Intensity_IntegratedIntensity_DNA	19	rs4239595	54033901	A	C	0.88	-0.27	0.05	7.97E-09	intcol	Intensity
Nuclei_RadialDistribution_FracAtD_Brightfield_4of4	19	rs8101204	54034083	T	C	0.88	-0.38	0.07	1.97E-08	isolate	RadialDistribution
Cells_RadialDistribution_MeanFrac_Brightfield_4of4	19	rs8101204	54034083	T	C	0.88	0.35	0.06	3.13E-08	intcol	RadialDistribution
Cytoplasm_Intensity_IntegratedIntensity_DNA	19	rs8101204	54034083	T	C	0.88	-0.29	0.05	1.37E-09	intcol	Intensity
Nuclei_RadialDistribution_FracAtD_Brightfield_4of4	19	rs34102215	54034354	T	C	0.88	-0.38	0.07	1.97E-08	isolate	RadialDistribution
Cells_RadialDistribution_MeanFrac_Brightfield_4of4	19	rs34102215	54034354	T	C	0.88	0.35	0.06	3.13E-08	intcol	RadialDistribution
Cytoplasm_Intensity_IntegratedIntensity_DNA	19	rs34102215	54034354	T	C	0.88	-0.29	0.05	1.37E-09	intcol	Intensity
Nuclei_RadialDistribution_FracAtD_Brightfield_4of4	19	rs35676049	54034360	C	T	0.88	-0.39	0.07	1.41E-08	isolate	RadialDistribution
Cells_RadialDistribution_MeanFrac_Brightfield_4of4	19	rs35676049	54034360	C	T	0.88	0.36	0.06	2.02E-08	intcol	RadialDistribution

Cytoplasm_Intensity_IntegratedIntensity_DNA	19	rs35676049	54034360	C	T	0.88	-0.30	0.05	6.81E-10	intcol	Intensity
Nuclei_RadialDistribution_FracAtD_Brightfield_4of4	19	rs34509494	54034589	A	G	0.88	-0.38	0.07	1.97E-08	isolate	RadialDistribution
Cells_RadialDistribution_MeanFrac_Brightfield_4of4	19	rs34509494	54034589	A	G	0.88	0.35	0.06	3.13E-08	intcol	RadialDistribution
Cytoplasm_Intensity_IntegratedIntensity_DNA	19	rs34509494	54034589	A	G	0.88	-0.29	0.05	1.37E-09	intcol	Intensity
Nuclei_RadialDistribution_FracAtD_Brightfield_4of4	19	rs36065332	54034603	G	A	0.88	-0.38	0.07	1.97E-08	isolate	RadialDistribution
Cells_RadialDistribution_MeanFrac_Brightfield_4of4	19	rs36065332	54034603	G	A	0.88	0.35	0.06	3.13E-08	intcol	RadialDistribution
Cytoplasm_Intensity_IntegratedIntensity_DNA	19	rs36065332	54034603	G	A	0.88	-0.29	0.05	1.37E-09	intcol	Intensity
Cells_RadialDistribution_MeanFrac_Brightfield_4of4	19	rs2438043	54034753	G	A	0.86	0.34	0.06	1.54E-08	intcol	RadialDistribution
Cytoplasm_Intensity_IntegratedIntensity_DNA	19	rs2438043	54034753	G	A	0.86	-0.26	0.05	1.82E-08	intcol	Intensity
Cytoplasm_Intensity_IntegratedIntensity_DNA	19	rs12610534	54035373	T	C	0.87	-0.27	0.05	4.06E-09	intcol	Intensity
Cells_RadialDistribution_MeanFrac_Mito_1of4	20	rs6110496	15016609	C	T	0.05	-0.47	0.08	2.46E-08	isolate	RadialDistribution
Nuclei_Intensity_MedianIntensity_Mito	20	20:30921787_G_GA	30921787	GA	G	0.11	-0.37	0.07	1.69E-08	intcol	Intensity
Nuclei_Intensity_MedianIntensity_Mito	20	20:30921800_C_T	30921800	C	T	0.12	-0.35	0.06	3.06E-08	intcol	Intensity
Cytoplasm_RadialDistribution_MeanFrac_ER_2of4	22	22:22279641_C_CCT	22279641	CCT	C	0.90	-0.40	0.07	3.20E-08	isolate	RadialDistribution
Cytoplasm_RadialDistribution_MeanFrac_Mito_2of4	22	22:22279641_C_CCT	22279641	CCT	C	0.90	-0.37	0.06	8.89E-09	isolate	RadialDistribution
