# Supplementary material for: High-dimensional phenotyping to define the genetic basis of cellular morphology
Source: Nat Commun. 2024 Jan 6;15:347. doi: 10.1038/s41467-023-44045-w (PMC10771466; doi:10.1038/s41467-023-44045-w)
Supplement: Supplementary file 3 — Reporting Summary [file 41467_2023_44045_MOESM3_ESM.pdf]

nature portfolio

Corresponding author(s): Shantanu Singh, Ralda Nehme, Soumya Raychaudhuri

Last updated by author(s): Oct 2, 2023

Reporting Summary

Nature Portfolio wishes to improve the reproducibility of the work that we publish. This form provides structure for consistency and transparency in reporting. For further information on Nature Portfolio policies, see our Editorial Policies and the Editorial Policy Checklist.

Statistics

For all statistical analyses, confirm that the following items are present in the figure legend, table legend, main text, or Methods section.

n/a	Confirmed	
☐	☒	The exact sample size (n) for each experimental group/condition, given as a discrete number and unit of measurement
☐	☒	A statement on whether measurements were taken from distinct samples or whether the same sample was measured repeatedly
☐	☒	The statistical test(s) used AND whether they are one- or two-sided<br>*Only common tests should be described solely by name; describe more complex techniques in the Methods section.*
☐	☒	A description of all covariates tested
☐	☒	A description of any assumptions or corrections, such as tests of normality and adjustment for multiple comparisons
☐	☒	A full description of the statistical parameters including central tendency (e.g. means) or other basic estimates (e.g. regression coefficient) AND variation (e.g. standard deviation) or associated estimates of uncertainty (e.g. confidence intervals)
☐	☒	For null hypothesis testing, the test statistic (e.g. F, t, r) with confidence intervals, effect sizes, degrees of freedom and P value noted<br>*Give P values as exact values whenever suitable.*
☒	☐	For Bayesian analysis, information on the choice of priors and Markov chain Monte Carlo settings
☒	☐	For hierarchical and complex designs, identification of the appropriate level for tests and full reporting of outcomes
☐	☒	Estimates of effect sizes (e.g. Cohen's d, Pearson's r), indicating how they were calculated

Our web collection on statistics for biologists contains articles on many of the points above.

Software and code

Policy information about availability of computer code

Data collection	CellProfiler versions 3.2, 4.1
Data analysis	Source code to reproduce and build upon the presented results is available at https://github.com/broadinstitute/cmQTL

For manuscripts utilizing custom algorithms or software that are central to the research but not yet described in published literature, software must be made available to editors and reviewers. We strongly encourage code deposition in a community repository (e.g. GitHub). See the Nature Portfolio guidelines for submitting code & software for further information.

Data

Policy information about availability of data

All manuscripts must include a data availability statement. This statement should provide the following information, where applicable:

- Accession codes, unique identifiers, or web links for publicly available datasets
- A description of any restrictions on data availability
- For clinical datasets or third party data, please ensure that the statement adheres to our policy

The WGS data generated in this study have been deposited in a publicly available Terra workspace https://app.terra.bio/#workspaces/anvil-datastorage/AnVIL_NIMH_Broad_ConvergentNeuro_McCarroll_Eggan_CIRM_GRU_WGS. The raw image data are available in the Cell Painting Gallery on the Registry of Open Data on AWS (https://registry.opendata.aws/cellpainting-gallery/) as dataset `cpg0022-cmqtl` at no cost and no need for registration.

Research involving human participants, their data, or biological material

Policy information about studies with human participants or human data. See also policy information about sex, gender (identity/presentation), and sexual orientation and race, ethnicity and racism.

Reporting on sex and gender	These findings do not apply to one sex or gender specifically. We designed our study such that we could have as equal distributions between male and female samples based on genetic sex. Of our 297 cell lines incorporated in this work, 153 were male and 144 were female.
Reporting on race, ethnicity, or other socially relevant groupings	We include self-reported ancestry when describing our iPSC cohort, which can be obtained from the CIRM iPSC repository website. In our downstream analyses, we used genetic PCs in order to account for confounding variables which may be related to ancestry.
Population characteristics	The population in our study averaged 21 years of age +/- 10, 70% reported they were of European ancestry while 30% indicated other. 62% of individuals had a clinical diagnosis. 52% were male and 48% were female based on sex imputation from genotyping data.
Recruitment	N/A
Ethics oversight	N/A

Note that full information on the approval of the study protocol must also be provided in the manuscript.

Field-specific reporting

Please select the one below that is the best fit for your research. If you are not sure, read the appropriate sections before making your selection.

☒ Life sciences ☐ Behavioural & social sciences ☐ Ecological, evolutionary & environmental sciences

For a reference copy of the document with all sections, see nature.com/documents/nr-reporting-summary-flat.pdf

Life sciences study design

All studies must disclose on these points even when the disclosure is negative.

Sample size	We chose this sample size due to the availability of cell lines within our collection which were consented for this work.
Data exclusions	NA- no data generated in our study was later excluded.
Replication	Given this was a GWAS/QTL study, there were no replicated experiments for our associations. Experiments related to image data generated were performed independently a few months apart.
Randomization	Samples were randomly grouped into batches of 48 cell lines. There was no process by which samples were introduced into any given batch and were allocated based on availability at the time of the experiment.
Blinding	Given this was a GWAS/QTL study, there were no treatment or control groups.

Reporting for specific materials, systems and methods

We require information from authors about some types of materials, experimental systems and methods used in many studies. Here, indicate whether each material, system or method listed is relevant to your study. If you are not sure if a list item applies to your research, read the appropriate section before selecting a response.

Materials & experimental systems

n/a	Involved in the study	
☒	☐	Antibodies
☐	☒	Eukaryotic cell lines
☒	☐	Palaeontology and archaeology
☒	☐	Animals and other organisms
☒	☐	Clinical data
☒	☐	Dual use research of concern
☒	☐	Plants

Methods

n/a	Involved in the study	
☒	☐	ChIP-seq
☒	☐	Flow cytometry
☒	☐	MRI-based neuroimaging

Eukaryotic cell lines

Policy information about cell lines and Sex and Gender in Research

Cell line source(s)	All cell lines were retrieved from the Stanley Center iPSC collection. The lines utilized within this work originated from the CIRM iPSC Repository. Cell line IDs (linked to the IDs for commercially available cells from CIRM) are listed here, as well as in Table S1 in the Supplementary Information.<br>CW10152<br>CW20002<br>CW60085<br>CW20058<br>CW20063<br>CW20069<br>CW20104<br>CW20245<br>CW20249<br>CW20311<br>CW20320<br>CW20324<br>CW30053<br>CW30095<br>CW30171<br>CW30438<br>CW30453<br>CW30463<br>CW60039<br>CW60051<br>CW60053<br>CW60054<br>CW60057<br>CW60084<br>CW60086<br>CW60087<br>CW60094<br>CW60095<br>CW60134<br>CW60135<br>CW60144<br>CW60163<br>CW60164<br>CW60166<br>CW60167<br>CW60181<br>CW60182<br>CW60184<br>CW60186<br>CW60188<br>CW60193<br>CW60195<br>CW60207<br>CW60218<br>CW60228<br>CW60234<br>CW60236<br>CW60297<br>CW60298<br>CW60308<br>CW60309<br>CW60333<br>CW60334<br>CW60335<br>CW60353<br>CW60354<br>CW60362<br>CW60365<br>CW60389<br>CW60391<br>CW60405

CW60417
CW60418
CW60427
CW60428
CW60469
CW60470
CW60477
CW60509
CW60510
CW70190
CW70219
CW60403
CW20012
CW20025
CW20047
CW20051
CW20073
CW20031
CW20050
CW20074
CW60092
CW60138
CW60139
CW60303
CW60331
CW60383
CW60390
CW20041
CW20075
CW20077
CW20081
CW20084
CW20087
CW20091
CW20094
CW20103
CW20105
CW20107
CW20108
CW20115
CW20132
CW20144
CW20146
CW20149
CW20166
CW20167
CW20183
CW20192
CW20193
CW20196
CW20200
CW20203
CW20210
CW20213
CW20214
CW20225
CW20239
CW20242
CW20243
CW50036
CW50037
CW60014
CW60015
CW60018
CW60026
CW60027
CW60048
CW60049

CW60055
CW60060
CW60070
CW60089
CW60097
CW60098
CW60108
CW60109
CW60122
CW60123
CW60124
CW60132
CW60133
CW60141
CW60142
CW60143
CW60145
CW60171
CW60172
CW60174
CW60175
CW60208
CW60209
CW60219
CW60220
CW60221
CW60222
CW60223
CW60242
CW60242
CW60277
CW60278
CW60279
CW60280
CW60289
CW60290
CW60302
CW60304
CW60330
CW60384
CW60404
CW60408
CW60410
CW60412
CW60456
CW60457
CW60503
CW60504
CW60520
CW60521
CW70347
CW70348
CW20053
CW60079
CW10054
CW10058
CW10077
CW10086
CW10100
CW10101
CW10162
CW10192
CW11072
CW20055
CW20178
CW20182
CW20252
CW30324

CW30369
CW30382
CW30420
CW30428
CW30429
CW30483
CW30485
CW40011
CW40106
CW40144
CW40214
CW40219
CW50147
CW60056
CW60131
CW60441
CW70117
CW70255
CW70261
CW70283
CW70303
CW80027
CW90034
CW70031
CW70140
CW20020
CW20042
CW20106
CW20133
CW30212
CW60040
CW60045
CW60058
CW60243
CW70356
CW40001
CW20184
CW20195
CW30108
CW30154
CW30190
CW30265
CW30274
CW30350
CW30421
CW30454
CW30484
CW30525
CW40067
CW40187
CW40201
CW50101
CW50106
CW60130
CW70004
CW70016
CW70142
CW70151
CW70164
CW70191
CW70280
CW70372
CW50094
CW70179
CW50032
CW70001
CW60242
CW70196

CW70211
CW40121
CW10064
CW10163
CW10202
CW20009
CW20026
CW20032
CW20033
CW20049
CW30178
CW30196
CW30280
CW30291
CW30292
CW30293
CW30299
CW30306
CW30307
CW30358
CW30383
CW30390
CW30391
CW40013
CW40197
CW40220
CW40228
CW50058
CW60029
CW60286
CW60288
CW60291
CW60305
CW60359
CW60420
CW60421
CW60480
CW60481
CW70235
CW70272
CW20140
CW10095
CW60026

Authentication	Cell lines were authenticated using genotyping data and we performed a concordance analyses to ensure donor identity.
Mycoplasma contamination	All cell lines tested negative for mycoplasma
Commonly misidentified lines (See ICLAC register)	We did not use any commonly misidentified cell lines in this study.
